# Discovery of a Series of Theophylline Derivatives Containing 1,2,3-Triazole for Treatment of Non-Small Cell Lung Cancer

**DOI:** 10.3389/fphar.2021.753676

**Published:** 2021-10-26

**Authors:** Jiahui Ye, Longfei Mao, Luoyijun Xie, Rongjun Zhang, Yulin Liu, Lizeng Peng, Jianxue Yang, Qingjiao Li, Miaomiao Yuan

**Affiliations:** ^1^ The Eighth Affiliated Hospital, Sun Yat-sen University, Shenzhen, China; ^2^ School of Chemistry and Chemical Engineering, Henan Engineering Research Center of Chiral Hydroxyl Pharmaceutical, Henan Normal University, Xinxiang, China; ^3^ Institute of Agro-Food Science and Technology Shandong Academy of Agricultural Sciences, Key Laboratory of Agro-Products Processing Technology of Shandong Province, Key Laboratory of Novel Food Resources Processing Ministry of Agriculture, Jinan, China; ^4^ Department of Neurology, The First Affiliated Hospital of Henan University of Science and Technology, Luoyang, China

**Keywords:** theophylline, 1,2,3-triazole, apoptosis, NSCLC, antitumor

## Abstract

Chemotherapy is the most common clinical treatment for non-small cell lung cancer (NSCLC), but low efficiency and high toxicity of current chemotherapy drugs limit their clinical application. Therefore, it is urgent to develop hypotoxic and efficient chemotherapy drugs. Theophylline, a natural compound, is safe and easy to get, and it can be used as a modified scaffold structure and hold huge potential for developing safe and efficient antitumor drugs. Herein, we linked theophylline with different azide compounds to synthesize a new type of 1,2,3-triazole ring-containing theophylline derivatives. We found that some theophylline1,2,3-triazole compounds showed a good tumor-suppressive efficacy. Especially, derivative d17 showed strong antiproliferative activity against a variety of cancer cells *in vitro,* including H460, A549, A2780, LOVO, MB-231, MCF-7, OVCAR3, SW480, and PC-9. It is worth noting that the two NSCLC cell lines H460 H and A549 are sensitive to compound d17 particularly, with IC50 of 5.929 ± 0.97 μM and 6.76 ± 0.25 μM, respectively. Compound d17 can significantly induce cell apoptosis by increasing the ratio of apoptotic protein Bax/Bcl-2 by downregulating the expression of phosphorylated Akt protein, and it has little toxicity to normal hepatocyte cells LO2 at therapeutic concentrations. These data indicate that these theophylline acetic acid-1,2,3-triazole derivatives may be potential drug candidates for anti-NSCLC and are worthy of further study.

## Introduction

It is reported that lung cancer is the deadliest cancer in men in developed countries (26.2%) and developing countries (22.3%) (Bray et al., 2018; Siegel et al., 2019). In 2020, there were 2.2 million new lung cancer cases worldwide, accounting for 11.4% of the total global new cases; the death toll from lung cancer was 1.782 million, accounting for 18.0% of the total global cancer deaths (Sung et al., 2021). Lung cancer falls into two categories, non-small cell lung cancer (NSCLC) and small cell lung cancer (SCLC). NSCLC is the most common type of lung cancer, further divided into squamous cell carcinoma (SCC), large cell carcinoma (LCC), and adenocarcinoma (AC) (Goldstraw et al., 2011; Travis et al., 2011). AC (accounting for 50% of total NSCLC cases) and SCC (accounting for 30% of total NSCLC cases) are the most common types of NSCLC (Lee and Cheah, 2019). Chemotherapy is the most commonly used treatment of NSCLC, but both single-agent chemotherapy and combination chemotherapy will bring a series of serious side effects, such as hair loss, anemia, nausea, and vomiting (Miller et al., 2016). Therefore, it is extremely urgent to design a safe, efficient, and less side-effect chemotherapy drug.

**FIGURE 1:**
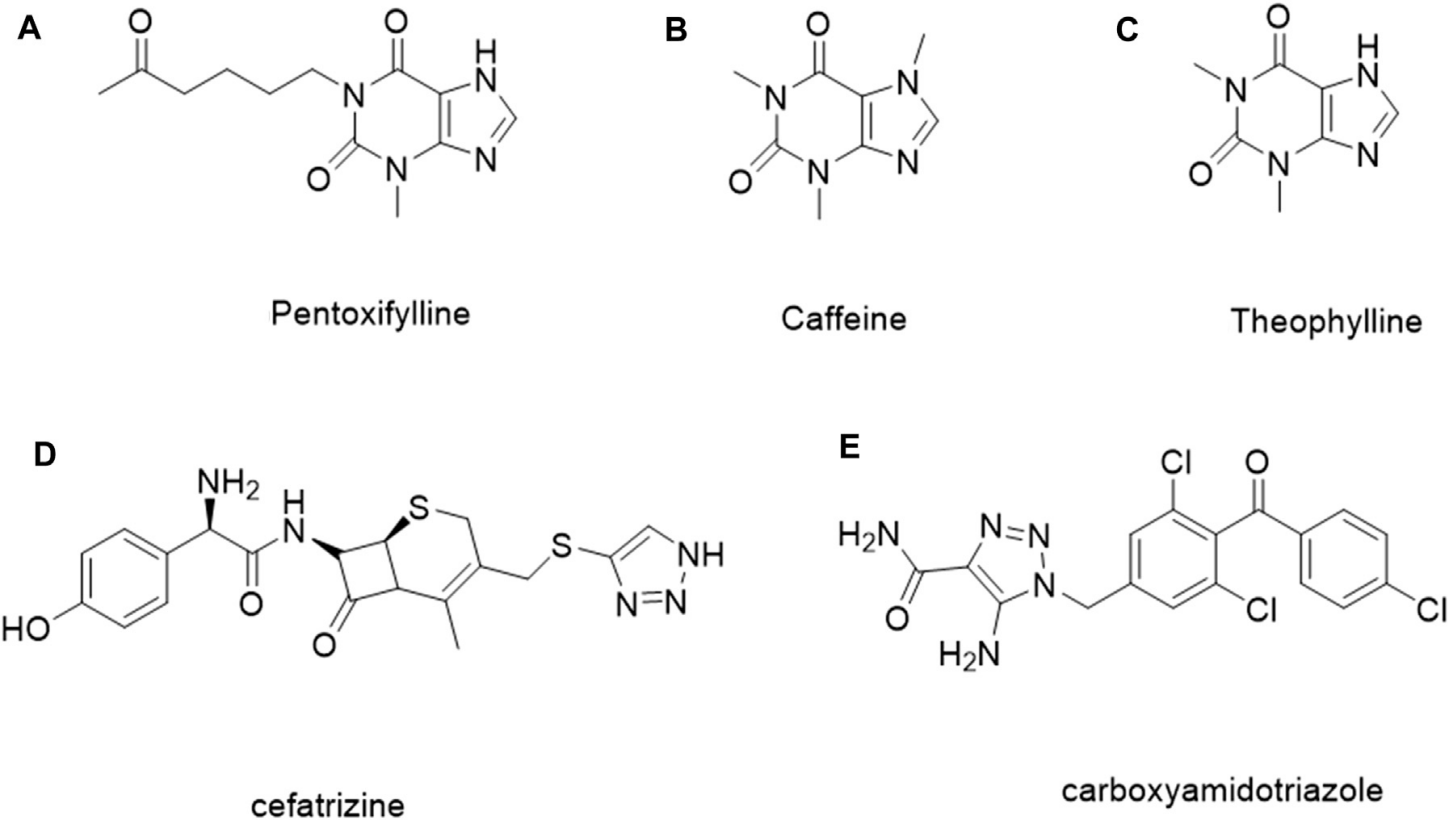
Examples of the methylxanthine-containing compounds and the reported 1, 2, 3-triazole derivatives for treating tumors.

It is estimated that methylxanthine-containing compounds, such as pentoxifylline (Figure 1A), can improve the efficacy of radiotherapy and chemotherapy and are used as chemotherapy sensitivity modifiers (Misirlioglu et al., 2007); caffeine (Figure 1B) and theophylline (Figure 1C) can enhance the toxicity of doxorubicin to tumor cells (Motegi et al., 2013; Yung-Lung Chang et al., 2017; David Osarieme et al., 2019; Liu et al., 2019). When theophylline is used in combination with gemcitabine or cisplatin, it has been found that theophylline can induce apoptosis in a variety of tumor cells (Hirsh et al., 2004). As a natural medicine, theophylline has a wide range of sources and low biological toxicity. Therefore, theophylline as a basic modified scaffold structure provides hope for developing safe and efficient antitumor drugs (Abou-Zied et al., 2019).

1, 2, 3-Triazole, as an important nitrogen heterocyclic structure, plays an important role in compound design and synthesis (Majeed et al., 2013). Compounds with the 1, 2, 3-triazole ring generally show good inhibitory activity against cancer, inflammation, and microorganisms (Rohrig et al., 2012; Zhao et al., 2012; Chen et al., 2017; Al-Blewi et al., 2018; Sakly et al., 2018). In addition, the 1, 2, 3-triazole ring can be easily constructed by the copper-catalyzed azide and alkyne cycloaddition reaction, which reduces the difficulty of synthesis and further improves the application potential. In addition, some compounds containing 1, 2, 3-triazole, such as ceftriaxone (Figure 1D) and carboxamide triazole (Figure 1E), have been used in clinics or are undergoing clinical trials for cancer treatment (Xu et al., 2019; Vanaparthi et al., 2020). Tazobactam is also used as an antibacterial agent (Karlowsky et al., 2020; Lob et al., 2020; Los-Arcos et al., 2020). 1, 2, 3-Triazole can hybridize with other anticancer pharmacophores or act as a linker connecting two anticancer pharmacophores, which make it in the design and synthesis of antitumor compounds widely (Bozorov et al., 2019; Aouad et al., 2021; Liang et al., 2021).

Based on the above, we combined the advantages of theophylline and 1, 2, 3-triazole, hoping to develop a novel series of safe and efficient theophylline-containing 1, 2, 3-triazole ring derivatives for the treatment of NSCLC. We expect that this combination will improve the antitumor activity of such compounds and solve safety issues. For example, recent studies demonstrate that a novel series of benzimidazole derivatives have cell-cycle inhibition and apoptotic effects against a panel of selected human cancer cell lines (Atmaca et al., 2020; Atmaca et al., 2021). The structural modification of this series of compounds holds great potential that leads to the discovery of a series of novel antitumor chemical compounds which combine the advantages of the original molecule with the introduced additional functional groups.

## Results and Discussion

### Chemistry

**SCHEME 1:**
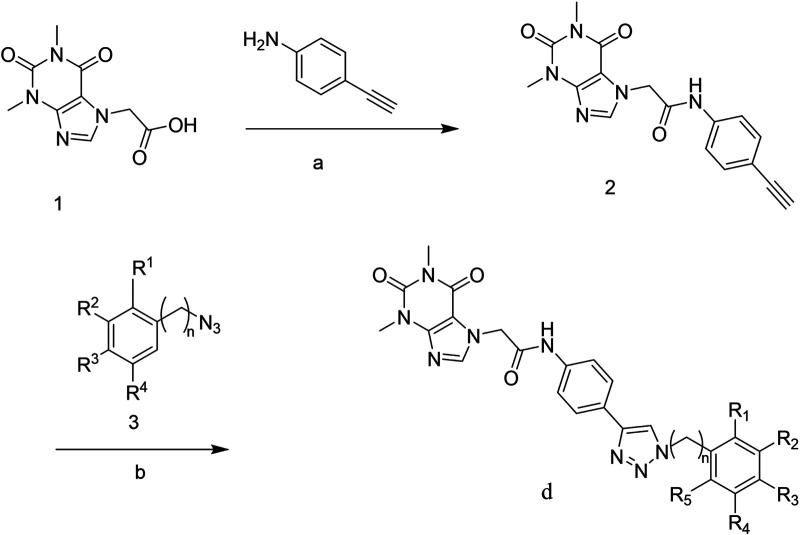
Reagents and conditions: **(A)** Theophylline acetic acid, 4-aminophenylacetylene, AHTU, and DIPEA were stirred in the DMF solvent 24 h at room temperature; **(B)** click reaction of copper sulfate water and sodium ascorbate in a solvent (tert-butanol: tetrahydrofuran: water = 1:1:1 at 85°C).

The strategy for preparing target compound d is shown in Scheme 1. Compound **2** was obtained after reaction of theophylline acetic acid (compound **1**) and 4-aminophenylacetylene. The target compounds **d1–d29** were gained through click reaction of compound **2** with different azido compounds. The reaction conditions of these operations were gentle and easy to control. The structures of the key intermediates and all target compounds were confirmed by nuclear magnetic resonance (1H NMR and 13C NMR) and high-resolution mass spectrometry (HRMS) (in Supplementary Material).

### *In Vitro* Antitumor Activity Study

IC_50_ values were obtained from three independent experiments. These results are reported as the average ± SD.

#### Proliferative Activity of Nine Human Cancer Cell Lines Was Inhibited by Theophylline-1,2,3-Triazole Derivatives

In order to screen out compounds with excellent antitumor activity from 31 theophylline acetic acid derivatives, we selected two tumor cells lines, A549 and MCF-7, as the treatment objects. The CCK8 assay was used to evaluate the effect of this series of theophylline acetic acid derivatives on A549 and MCF-7 proliferative activity. As shown in Table 1, both A549 and MCF-7 are not sensitive to theophylline acetic acid [half-maximal inhibitory concentration (IC_50_) >100 μM]. A549 is only sensitive to **d17** (IC50 = 6.76 ± 0.25) but not sensitive to theophylline acetic acid and other theophylline-1, 2, 3-triazole derivatives. For MCF−7, **d1** (IC50 = 60.97 ± 9.74), **d6** (IC50 = 45.24 ± 3.23), **d17** (IC50 = 12.61 ± 3.48), **d19** (IC50 = 59.01 ± 2.68), and **d28** (IC50 = 80.69 ± 17.77) are sensitive. Although the number of compounds sensitive to MCF-7 is more than A549, A549 has the best sensitivity to compound **d17** (IC50 = 6.76 ± 0.25), and MCF−7 also shows moderate sensitivity to compound **d17**, so we chose compound **d17** to carry out the study.

**TABLE 1:** Antitumor activities of the designed compounds against two cancer cells lines *in vitro*.

Compound no	n	R^1^	R^2^	R^3^	R^4^	R^5^	IC_50(_μM)	
							A549	MCF-7
d-1	0	CH_3_	NO_2_	H	H	H	>100	60.97 ± 9.74
d-2	1	Cl	H	H	H	H	>100	>100
d-3	0	H	H	H	H	H	>100	>100
d-4	1	H	OCH_3_	H	H	H	>100	>100
d-5	0	F	H	H	H	H	>100	>100
d-6	1	H	H	Cl	H	H	>100	45.24 ± 3.23
d-7	1	CF_3_	H	H	H	H	>100	>100
d-8	1	H	H	H	H	H	>100	>100
d-9	1	Br	H	H	H	H	>100	>100
d-10	1	H	H	CF_3_	H	H	>100	>100
d-11	0	OCF_3_	H	H	H	H	>100	>100
d-12	0	H	CF_3_	CF_3_	H	H	>100	>100
d-13	0	H	CH_3_	CH_3_	H	H	>100	>100
d-14	0	CF_3_	H	H	H	H	>100	>100
d-15	0	CH_2_CH_3_	H	H	H	H	>100	>100
d-16	0	CH_3_	H	CH_3_	H	CH_3_	>100	>100
**d-17**	**0**	**CF**_**3**_	**H**	**H**	**CF**_**3**_	**H**	**6.76 ± 0.25**	**12.61 ± 3.48**
d-18	0	H	F	H	H	H	>100	>100
d-19	0	Cl	H	H	H	H	>100	59.01 ± 2.68
d-20	0	H	Br	H	H	H	>100	>100
d-21	0	H	CF_3_	H	CF_3_	H	>100	>100
d-22	0	I	H	H	H	H	>100	>100
d-23	0	H	Cl	H	H	H	>100	>100
d-24	0	H	H	Cl	H	H	>100	>100
d-25	0	H	OCH_3_	H	H	H	>100	>100
d-26	0	OCH_3_	H	H	H	H	>100	>100
d-27	0	H	H	F	H	H	>100	>100
d-28	0	Br	H	H	H	H	>100	80.69 ± 17.77
d-29	0	H	H	CF_3_	H	H	>100	>100
Theophylline acetic acid	**—**	**—**	**—**	**—**	**—**	**—**	**>100**	**>100**

**FIGURE 2:**
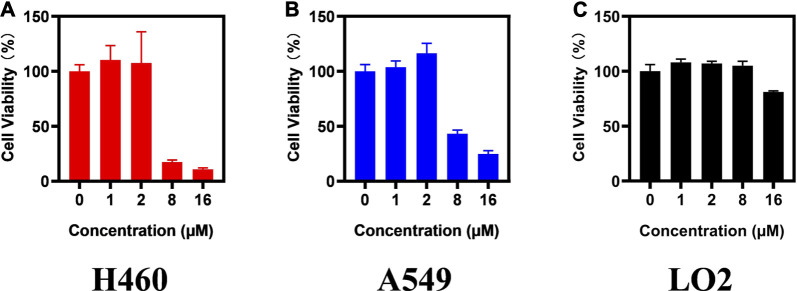
Compound d17 supresses H460 and A549 cancer cells. H460 **(A)**, A549 **(B),** and LO2 **(C)** cells were exposed to compound d17 with indicated concentrations for 72 h, and cell viability was assessed by the CCK-8 assay, *n* = 3. *p-value < 0.05, **p-value < 0.01, and ***p-value < 0.001 (one-way ANOVA, followed by Tukey’s post-test).

To confirm the antitumor activity of compound **d17** and screen out the most sensitive cell line to compound **d17**, we added seven cell lines, H460, A2780, LOVO, MB-231, OVCAR3, SW480, and PC9, as treatment objects. As shown in Table 2, compound **d17** showed strong antiproliferative and cytotoxicity to these nine cancer cell lines, H460 (IC50 = 5.93 ± 0.97 μM), A549 (IC50 = 6.76 ± 0.25 μM), A2780 (IC50 = 26.84 ± 6.96 μM), LOVO (IC50 = 37.42 ± 0.82 μM), MB-231 (IC50 = 18.78 ± 3.84 μM), MCF-7 (IC50 = 12.61 ± 1.76 μM), OVCAR3 (IC50 = 29.33 ± 6.20 μM), SW480 (IC50 = 15.66 ± 2.37 μM), and PC9 (IC50 = 18.20 ± 14.15 μM). Among these nine cell lines, H460 and A549 are the most sensitive cell lines to compound **d17**, with IC50 of 5.93 ± 0.97 μM Figure 2A and 8.926 μM (Figure 2B), respectively. In addition, we also measured the cytotoxicity of compound **d17** to normal liver cells LO2 (Figure 2C), and the results showed that at an effective therapeutic concentration (8 μM), the cytotoxicity of **d17** to normal liver cells was almost 0; when the compound concentration reached 16 μM, it had a little inhibitory effect on LO2.

**TABLE 2:** Antiproliferative activities of compounds d17 against nine human cancer cell lines and normal liver cell lines.

Compound no	IC_50_ (μM)									
	H460	A549	A2780	LOVO	MB-231	MCF-7	OVCAR3	SW480	PC-9	LO2
**d17**	**5.93 ± 0.97**	**6.76 ± 0.25**	26.84 ± 6.96	37.42 ± 0.82	18.78 ± 3.84	12.61 ± 1.76	29.33 ± 6.20	15.66 ± 2.37	18.20 ± 14.15	29.24 ± 3.74

IC_50_ values were obtained from three independent experiments. These results are reported as the average ± SD.

**FIGURE 3:**
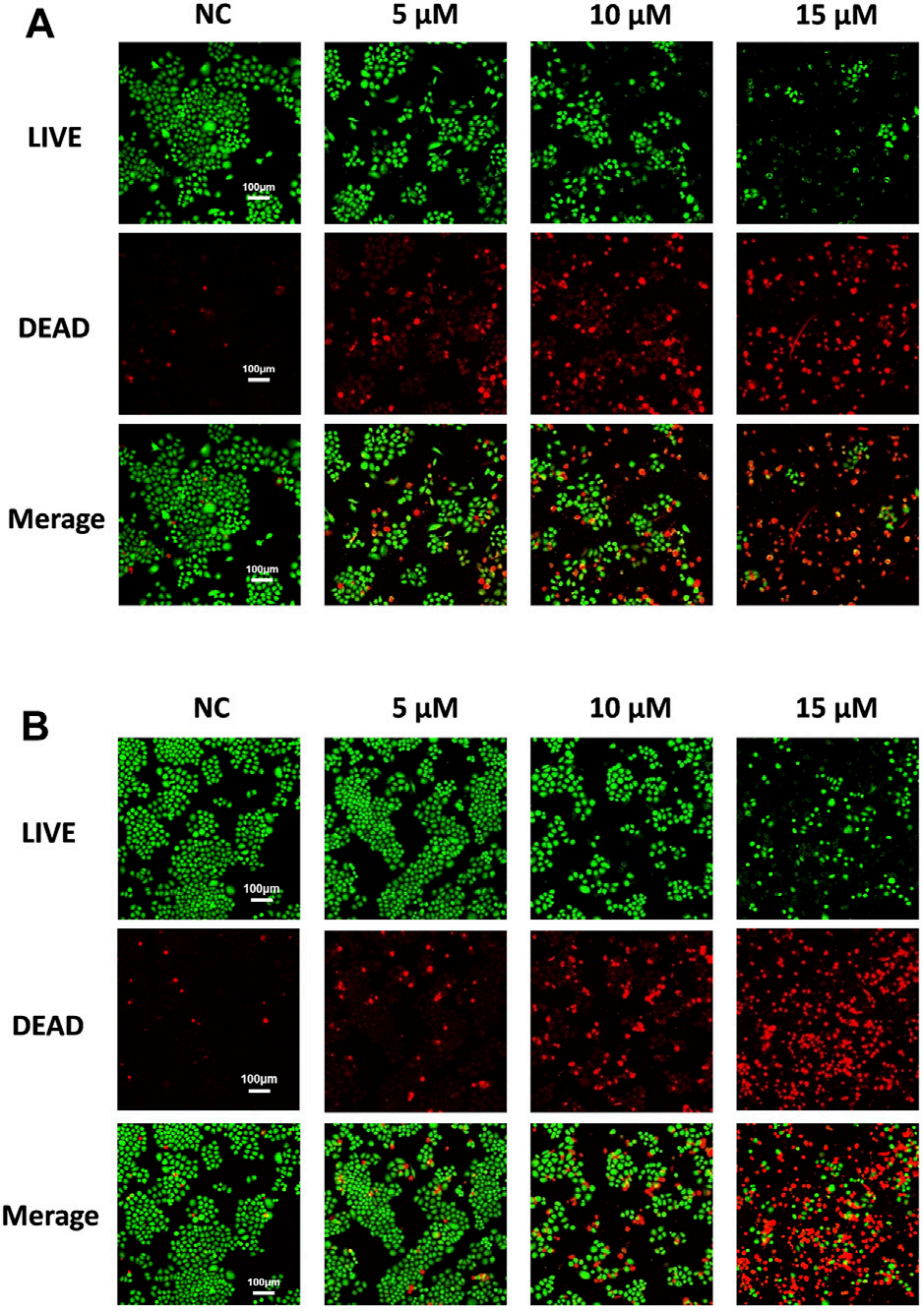
Compound d17 suppresses H460 and A549 cancer cells. Fluorescence images of **(A)** H460 and **(B)** A549 cells exposed to compound d17 with indicated concentrations for 48 h and then stained with the red/green kit; green indicates live cells, and red indicates dead cells.

To further evaluate the anti-NSCLC activity of compound **d17**, we used LIVE/DEAD staining. As shown in Figure 3, the number of dead cells increased as the concentration of compound **d17** increased, which was consistent with the results of CCK8 determination. In short, these results indicate that compound **d17** can effectively inhibit the proliferative activity of NSCLC and has little cytotoxicity to normal hepatocytes at effective therapeutic concentrations.

#### Theophylline1, 2, 3-Triazole Derivatives Suppress NSCLC Cell Lines by Inducing Apoptosis

**FIGURE 4:**
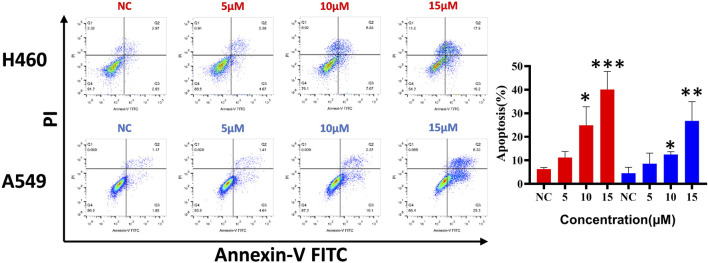
Compound d17 induced apoptosis of H460 and A549. Flow cytometry analysis data from three independent experiments were summarized and shown. NC, negative control. *p-value < 0.05, **p-value < 0.01, and ***p-value < 0.001 (one-way ANOVA, followed by Tukey’s post-test).

To clarify whether the antiproliferative effect is related to cell apoptosis, H460 and A549 cells were treated with different concentrations (5, 10, and 15 μM) of compound **d17** for 48 h and then detected by flow cytometry. As shown in Figure 4, we observed significant apoptosis in H460 and A549 cells exposed to different concentrations of **d17**. The proportions of H460 apoptotic cells treated with compound d17 were 11.19% (5 μM), 24.89% (10 μM), and 40.09% (15 μM), while the proportions of A549 apoptotic cells treated with compound d17 were 8.55% (5 μM), 12.47% (10 μM), and 26.76% (15 μM). These results suggested that compound d17 considerably promoted the apoptosis of lung cancer cell lines H460 and A549 in a concentration-dependent manner.

#### Theophylline1, 2, 3-Triazole Derivatives Trigger Apoptosis by Suppressing Phosphorylation of Akt Protein

**FIGURE 5:**
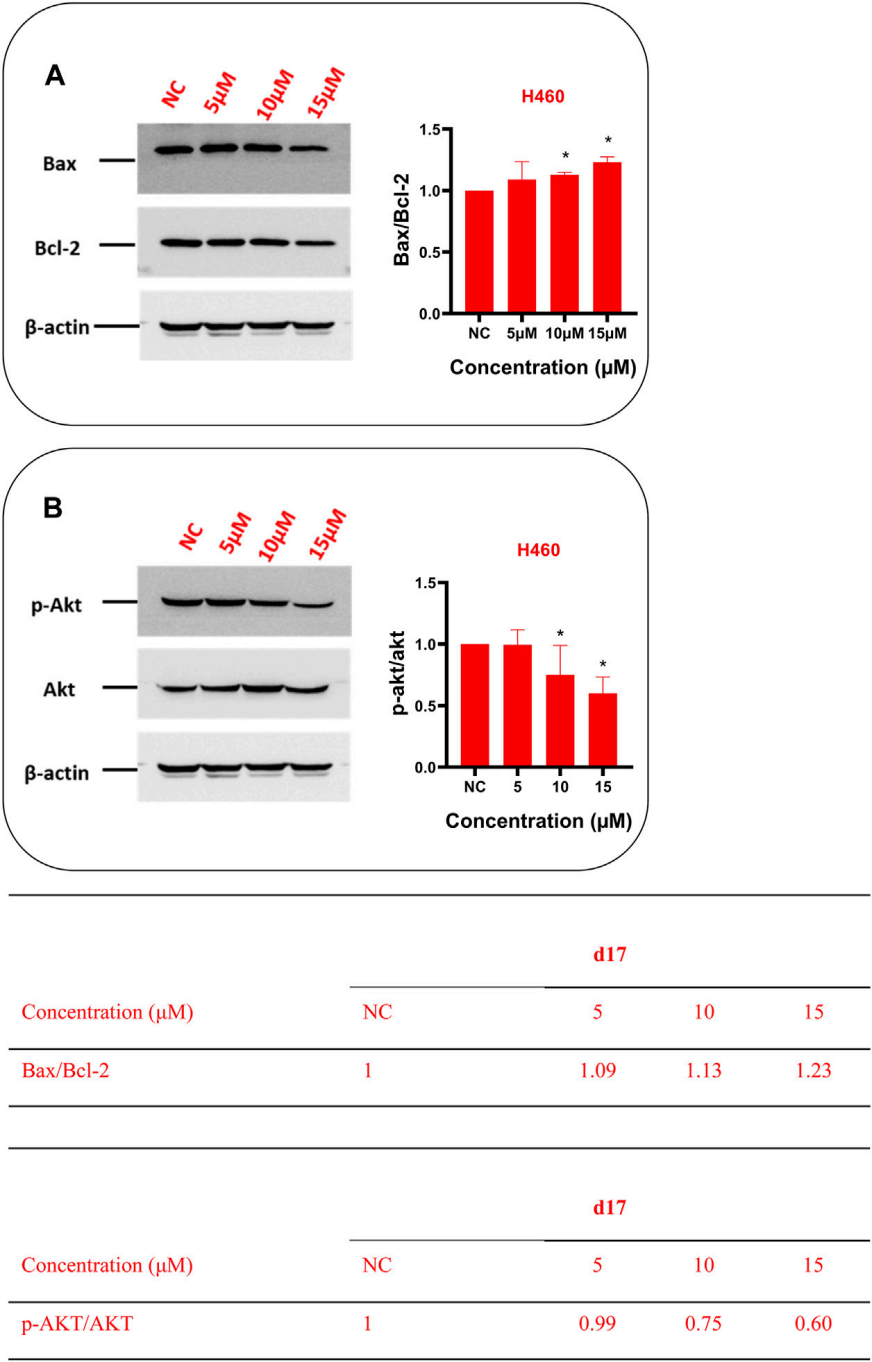
Compound d17 suppressed Akt phosphorylation and its transduction of downstream signaling Bax and Bcl-2 in NSCLC cells. Western blot was used to detect apoptosis-related markers Bax, Bcl-2 **(A)**, and Akt **(B)**. Protein bands (left images) and quantification (right images and tables below) are presented. NC, negative control. *p-value < 0.05, **p-value < 0.01, and ***p-value < 0.001 (one-way ANOVA, followed by Tukey’s post-test).

In order to further explore the mechanism of **d17**-induced apoptosis in NSCLC, western blot was used to detect apoptosis-related markers Bax, Bcl-2 (Figure 5A), and Akt (Figure 5B). As shown in Figure 5, after H460 cells were treated with 0.1% DMSO as control or different concentrations of compound **d17** for 24 h, total cell protein analysis showed that the p-Akt protein level in H460 cells was lower than that in the control group, and the ratio of p-Akt/Akt is also lower than that in the control group, and as the drug concentration increases, the ratio of p-Akt/Akt decreases. The levels of apoptosis inhibitor protein Bcl-2 and apoptosis marker protein Bax both decreased with the increase of drug concentration, but the ratio of Bax/Bcl-2 increased with the increase of drug concentration. Phosphorylated Akt protein can inhibit apoptosis by inhibiting the function of Bax protein, and various studies have reported that the overexpression of phosphorylated AKT (*p*-AKT) is a key defect in many types of solid tumors (Atmaca et al., 2017; Brown and Banerji, 2017; Shariati and Meric-Bernstam, 2019; Song et al., 2019; Iida et al., 2020). Compound **d17** can inhibit the phosphorylation of Akt protein, which indicates that compound **d17** can increase the ratio of apoptotic protein Bax/Bcl-2 and promotes NSCLC cell apoptosis by inhibiting the phosphorylation of Akt protein.

## Conclusion

In a word, we designed and synthesized a series of theophylline derivatives containing the 1, 2, 3-triazole ring and evaluated their antiproliferative activity on nine kinds of cancer cells. Some of these compounds showed significant antitumor activity compared to theophylline acetic acid against one or more cancer cell lines used in this study. Among them, compound **d17** showed strong antiproliferation and cytotoxicity to all nine kinds of cancer cells, and the two NSCLC, H460 and A549, show the most sensitivity to compound **d17** particularly. We revealed the potential mechanism of d17-induce NSCLC cell death is that compound d17 through inhibiting Akt protein phosphorylation to induce mitochorylation appotosis. Current research shows that when appropriate substituents are introduced into the original molecule, the structural diversity of drugs can be expanded. Future research will focus on improving the anticancer activity and pharmacokinetic properties of these compounds.

## Experimental Section

### General Experimental Procedures

The theophylline acetic acid, 4-aminophenylacetylene, and azido compounds were purchased from Aladdin (CHINA). The RPMI-1640 medium, Dulbecco’s modified Eagle’s medium (DMEM), fetal bovine serum (FBS), trypsin, and phosphate-buffered saline (PBS) were purchased from Gibco (United States). The cell Counting Kit-8 (CCK-8) was purchased from Abmole (United States). An Annexin V/propidium iodide (PI) staining kit was purchased from BD Biosciences (United States). Akt, AKT1 (phospho S473), and the secondary antibodies of antirabbit and antimouse were purchased from Cell Signaling Technology, Inc. (United States). NSCLC cell lines PC-9, H460, and A549 and other cancer cell lines A2780, LOVO, MB-231, MCF-7, OVCAR3, and SW480 were obtained from ATCC.

### Chemistry

The general procedures of preparation for erlotinib and compounds d1–d29 were described in the section of results. The structures of all target compounds were confirmed by nuclear magnetic resonance (^1^H NMR and 13C NMR) and high-resolution mass spectrometry (HRMS) as below.

Theophylline acetic acid [compound 1 (5 g, 0.02 mol)], 4-aminophenylacetylene (3.69 g, 0.0 3 mol), HATU (12.96 g, 0.03 mol), and DIPEA (8.13 g, 0.06 mol) were added together into a 500 ml reaction flask in DMF, stirring for 24 h at room temperature under nitrogen protection. The reaction process was monitored by thin-layer chromatography (TLC). After the reaction was completed, DMF was removed with an oil pump; dichloromethane was added and washed with saturated salt water; the organic phase was combined, dried with anhydrous sodium sulfate, and concentrated in vacuum to obtain solid compound 2.

Benzyl bromide and sodium azide were stirred in a solvent (acetone: water = 4:1) for 24 h at room temperature to produce benzyl azide 3 (*n* = 1). Aniline is added to the solvent (water:hydrochloric acid = 1:1) and stirred (below 5°), and then, sodium nitrite is dissolved in water, slowly dripping in the solvent (water:hydrochloric acid = 1:1). Finally, sodium azide is dissolved in water, slowly dripping in the solvent (water:hydrochloric acid = 1:1) too, reacting for 24 h to obtain phenyl azide 3 (n = 0).

The azide compound (1.2 mmol) and compound 2 (1.0 mmol) were added to 15 ml of a mixed solvent (tetrahydrofuran:water:tert-butanol = 1:1:1). Anhydrous copper sulfate (0.1 mmol) and sodium ascorbate (0.2 mmol) were added, and the mixture was stirred at 80°C for 8 h. Upon completion of the reaction (monitored by TLC), the mixture was extracted with dichloromethane (15 ml × 3). All the organic phases were continuously washed with water and brine, dried with anhydrous sodium sulfate, and concentrated in vacuum. The residue was purified by column chromatography (dichloromethane:methanol = 20∶1) to obtain the target compounds d1–d29 in the white powder form.

2-(1,3-dimethyl-2,6-dioxo-1,2,3,6-tetrahydro-7H-purin-7-yl)-N-(4-(1-(2-methyl-3-nitrophenyl)-1H-1,2,3-triazol-4-yl)phenyl)acetamide (d1). ^1^H NMR (400 MHz, DMSO-*d*_6_): δ 10.58 (s, 1H), 8.97 (s, 1H), 8.19 (d, *J* = 7.3, 1H), 8.09 (s, 1H), 7.94–7.89 (m, 3H), 7.74–7.69 (m, 3H), 5.24 (s, 2H), 3.47 (s, 3H), 3.21 (s, 3H), 2.24 (s, 3H). ^13^C NMR (100 MHz, DMSO-d6) δ 165.50, 155.00, 151.49, 151.26, 148.44, 147.00, 144.26, 139.11, 138.17, 131.46, 128.74, 128.50, 126.51, 126.09, 125.80, 123.66, 119.89, 106.95, 49.25, 29.95, 27.94, 14.45. HR MS (ESI) *m/z*: calcd for C_24_H_22_ N_9_O_5_ [M + H]^+^ 516.1744, found 516.1741.

N-(4-(1-(2-chlorobenzyl)-1H-1,2,3-triazol-4-yl)phenyl)**-**2-(1,3-dimethyl-2,6-dioxo-1,2,3,6-tetrahydro-7H-purin-7-yl)acetamide (d2). ^1^H NMR (400 MHz, DMSO) δ 10.53 (s, 1H), 8.53 (s, 1H), 8.08 (s, 1H), 7.82 (d, *J* = 8.3, 2H), 7.65 (d, *J* = 8.3, 2H), 7.54 (d, *J* = 7.6, 1H), 7.43–7.37 (m, 2H), 7.28 (d, *J* = 7.0, 1H), 5.75 (d, *J* = 7.5, 2H), 5.22 (s, 2H), 3.46 (s, 3H), 3.20 (s, 3H). ^13^C NMR (100 MHz, DMSO-*d*_6_) δ 165.41, 154.98, 151.49, 148.43, 146.69, 144.25, 138.75, 133.64, 133.09, 130.99, 130.73, 130.10, 128.25, 126.36, 126.29, 121.85, 119.80, 106.93, 51.23, 49.22, 29.94, 27.92. HR MS (ESI) *m/z*: calcd for C_24_H_22_ ClN_8_O_3_ [M + H]^+^ 505.1503, found 505.1501.

2-(1,3-dimethyl-2,6-dioxo-1,2,3,6-tetrahydro-7H-purin-7-yl)-N-(4-(1-phenyl-1H-1,2,3**-**triazol-**4-**yl)phenyl)acetamide (d3). ^1^H NMR (400 MHz, DMSO-*d*_6_) δ 10.58 (s, 1H), 9.23 (s, 1H), 8.09 (s, 1H), 7.93 (dd, *J*_*1*_ = 14.5, *J*_*2*_ = 8.2, 4H), 7.71 (d, *J* = 8.4, 2H), 7.64 (t, *J* = 7.7, 2H), 7.52 (t, *J* = 7.3, 1H), 5.24 (s, 2H), 3.47 (s, 3H), 3.21 (s, 3H). ^13^C NMR (100 MHz, DMSO-*d*_6_) δ 165.50, 155.00, 151.50, 148.45, 147.54, 144.27, 139.05, 137.14, 129.16, 126.46, 126.01, 120.44, 119.90, 106.96, 49.26, 29.96, 27.95. HR MS (ESI) *m/z*: calcd for C_23_H_21_N_8_O_3_ [M + H]^+^ 457.1737, found 457.1737.

2-(1,3-dimethyl-2,6-dioxo-1,2,3,6-tetrahydro-7H-purin-7-yl)-N-(4-(1-(3-methoxybenzyl)-1H-1,2,3-triazol-4-yl)phenyl)acetamide (d4). ^1^H NMR (400 MHz, DMSO-*d*_6_) δ 10.53 (s, 1H), 8.55 (s, 1H), 8.08 (s, 1H), 7.81 (d, *J* = 8.3, 2H), 7.65 (d, *J* = 8.3, 2H), 7.30 (t, *J* = 7.8, 1H), 6.91 (dd, *J*_*1*_ = 16.6, *J*_*2*_ = 8.0, 3H), 5.60 (s, 2H), 5.22 (s, 2H), 3.75 (s, 3H), 3.46 (s, 3H), 3.20 (s, 3H). ^13^C NMR (100 MHz, DMSO-*d*_6_) δ 165.40, 159.94, 154.98, 151.49, 148.43, 146.86, 144.25, 138.71, 137.89, 130.43, 126.46, 126.24, 121.51, 120.45, 119.81, 114.20, 113.95, 106.94, 55.59, 53.40, 29.94, 27.93. HR MS (ESI) *m/z*: calcd for C_25_H_25_N_8_O_4_ [M + H]^+^ 501.1999, found 501.2004.

2-(1,3-dimethyl-2,6-dioxo-1,2,3,6-tetrahydro-7H-purin-7-yl)-N-(4-(1-(2-fluorophenyl)-1H-1,2,3-triazol-4-yl)phenyl)acetamide (d5). ^1^H NMR (400 MHz, DMSO-*d*_6_) δ 10.59 (s, 1H), 9.01 (s, 1H), 8.10 (s, 1H), 7.92 (s, 3H), 7.67 (d, *J* = 31.5, 4H), 7.48 (s, 1H), 5.24 (s, 2H), 3.46 (d, *J* = 4.5, 3H), 3.21 (s, 3H). ^13^C NMR (100 MHz, DMSO-*d*_6_) δ 144.26, 139.10, 126.55, 126.46, 126.10, 119.88, 49.25, 40.40, 40.19, 29.95, 27.94. HR MS (ESI) *m/z*: calcd for C_23_H_20_FN_8_O_3_ [M + H]^+^ 475.1642, found 475.1651.

N-(4-(1-(4-chlorobenzyl)-1H-1,2,3-triazol-4-yl)phenyl)**-**2-(1,3-dimethyl-2,6-dioxo-1,2,3,6-tetrahydro-7H-purin-7-yl)acetamide (d6). ^1^H NMR (400 MHz, DMSO-*d*_6_) δ 10.51 (s, 1H), 8.54 (s, 1H), 8.08 (s, 1H), 7.80 (d, *J* = 8.7, 2H), 7.64 (d, *J* = 8.7, 2H), 7.48–7.43 (m, 2H), 7.37 (d, *J* = 8.5, 2H), 5.64 (s, 2H), 5.22 (s, 2H), 3.46 (s, 3H), 3.20 (s, 3H). ^13^C NMR (100 MHz, DMSO-*d*_6_) δ 165.40, 154.99, 151.50, 148.45, 146.92, 144.26, 138.74, 135.45, 133.34, 129.26, 126.26, 121.56, 119.84, 106.95, 52.67, 49.22, 29.94, 27.93. HR MS (ESI) *m/z*: calcd for C_24_H_22_ClN_8_O_3_ [M + H]^+^ 505.1503, found 505.1504.

2-(1,3-dimethyl-2-oxo-1,2,3,6-tetrahydro-7H-purin-7-yl)-N-(4-(1-(2-(trifluoromethyl)benzyl)**-**1H-1,2,3-triazol-4-yl)phenyl)acetamide (d7). ^1^H NMR (400 MHz, DMSO-*d*_6_) δ 10.55 (s, 1H), 8.55 (s, 1H), 8.08 (s, 1H), 7.82 (s, 3H), 7.65 (d, J = 44.0, 4H), 7.24 (d, J = 4.4, 1H), 5.83 (s, 2H), 5.23 (s, 2H), 3.46 (s, 3H), 3.20 (s, 3H). ^13^C NMR (100 MHz, DMSO-*d*_6_) δ 165.42, 154.98, 148.43, 146.83, 144.26, 138.81, 133.73, 130.70, 129.39, 126.73, 126.67, 126.30, 122.09, 119.80, 106.94, 52.47, 50.16, 49.23, 39.99.29.95, 27.93, 7.64. HR MS (ESI) *m/z*: calcd for C_25_H_22_F_3_N_8_O_3_ [M + H]^+^ 539.1767, found 539.1766.

N-(4-(1-benzyl-1H-1,2,3-triazol-4-yl)phenyl)-2-(1,3-dimethyl-2,6-dioxo-1,2,3,6-tetrahydro-7H-purin-7-yl)acetamide (d8). ^1^H NMR (400 MHz, DMSO-*d*_6_) δ 10.53 (s, 1H), 8.56 (s, 1H), 8.09 (s, 1H), 7.80 (s, 2H), 7.68–7.62 (m, 2H), 7.42–7.31 (m, 5H), 5.64 (s, 2H), 5.22 (s, 2H), 3.46 (s, 3H), 3.20 (s, 3H). ^13^C NMR (100 MHz, DMSO-*d*_6_) δ 165.41, 146.87, 144.25, 136.47, 129.26, 128.62, 128.36, 126.24, 121.52, 119.81, 53.48, 49.23, 40.16, 29.94, 27.93. HR MS (ESI) *m/z*: calcd for C_24_H_23_N_8_O_3_ [M + H]^+^ 471.1893, found 471.1903.

N-(4-(1-(2-bromobenzyl)-1H-1,2,3-triazol-4-yl)phenyl)-2-(1,3-dimethyl-2,6-dioxo-1,2,3,6-tetrahydro-7H-purin-7-yl)acetamide (d9). ^1^H NMR (400 MHz, DMSO-*d*_6_) δ 10.51 (s, 1H), 8.51 (s, 1H), 8.08 (s, 1H), 7.82 (d, *J* = 8.6, 2H), 7.68 (dd, *J*_*1*_ = 21.2, *J*_*2*_ = 8.3, 3H), 7.45–7.40 (m, 1H), 7.33 (d, *J* = 16.8, 1H), 7.22 (d, *J* = 8.9, 1H), 5.72 (s, 2H), 5.22 (s, 2H), 3.46 (s, 3H), 3.20 (s, 3H). ^13^C NMR (100 MHz, DMSO-*d*_6_) δ 165.40, 154.99, 151.50, 148.44, 146.70, 144.26, 138.76, 135.26, 133.38, 128.80, 126.39, 126.30, 123.34, 121.88, 119.84, 106.95, 53.57, 49.23, 29.94, 27.92. HR MS (ESI) *m/z*: calcd for C_24_H_22_BrN_8_O_3_ [M + H]^+^ 549.0998, found 549.1008.

2-(1,3-dimethyl-2,6-dioxo-1,2,3,6-tetrahydro-7H-purin-7-yl)-N-(4-(1-(4-(trifluoromethyl)benzyl)**-**1H-1,2,3-triazol-4-yl)phenyl)acetamide (d10). ^1^H NMR (400 MHz, DMSO-*d*_6_) δ 10.53 (s, 1H), 8.60 (s, 1H), 8.08 (s, 1H), 7.79 (dd, *J*_*1*_ = 16.5, *J*_*1*_ = 8.4, 4H), 7.66 (d, *J* = 8.7, 2H), 7.54 (d, *J* = 8.1, 2H), 5.77 (s, 2H), 5.23 (s, 2H), 3.46 (s, 3H), 3.20 (s, 3H). ^13^C NMR (100 MHz, DMSO-*d*_6_) δ 165.42, 154.99, 151.49, 148.44, 146.99, 144.26, 141.15, 138.79, 129.07, 126.35, 126.28, 126.22, 126.18, 121.81, 119.82, 106.94, 52.81, 49.23, 29.95, 27.93. HR MS (ESI) *m/z*: calcd for C_25_H_22_F_3_N_8_O_3_ [M + H]^+^ 539.1767, found 539.1776.

2-(1,3-dimethyl-2,6-dioxo-1,2,3,6-tetrahydro-7H-purin-7-yl)-N-(4-(1-(2-(trifluoromethoxy)phenyl)**-**1H-1,2,3-triazol-4-yl)phenyl)acetamide(d11). ^1^H NMR (400 MHz, DMSO-*d*_6_) δ 10.58 (s, 1H), 8.98 (s, 1H), 8.10 (s, 1H), 7.91 (t, *J* = 7.5, 3H), 7.76– 8 (m, 5H), 5.25 (s, 2H), 3.47 (s, 3H), 3.21 (s, 3H). ^13^C NMR (100 MHz, DMSO-*d*_6_) δ 165.49, 155.00, 151.50, 148.45, 146.97, 144.26, 141.61, 139.11, 132.10, 130.20, 129.30, 128.02, 126.50, 125.76, 123.04, 119.94, 106.95, 49.25, 40.23, 29.94, 27.92. HR MS (ESI) *m/z*: calcd for C_24_H_20_F_3_N_8_O_4_ [M + H]^+^ 541.1560, found 541.1568.

2-(1,3-dimethyl-2,6-dioxo-1,2,3,6-tetrahydro-7H-purin-7-yl)-N-(4-(1-(3-(trifluoromethyl)phenyl)**-**1H-1,2,3-triazol-4-yl)phenyl)acetamide (d12). ^1^H NMR (400 MHz, DMSO-*d*_6_) δ 10.60 (s, 1H), 9.41 (s, 1H), 8.32 (s, 2H), 8.09 (s, 1H), 7.91 (d, *J* = 9.1, 4H), 7.73 (d, *J* = 8.4, 2H), 5.25 (s, 2H), 3.47 (s, 3H), 3.21 (s, 3H). ^13^C NMR (100 MHz, DMSO-*d*_6_) δ 165.52, 155.00, 151.50, 148.45, 147.79, 144.26, 139.18, 137.58, 131.88, 126.48, 125.74, 124.28, 119.94, 116.98, 106.96, 49.26, 29.96, 27.94. HR MS (ESI) *m/z*: calcd for C_24_H_20_F_3_N_8_O_3_ [M + H]^+^ 525.1610, found 525.1623.

2-(1,3-dimethyl-2,6-dioxo-1,2,3,6-tetrahydro-7H-purin-7-yl)-N-(4-(1-(m**-**tolyl)-1H-1,2,3-triazol-4-yl)phenyl)acetamide (d13). ^1^H NMR (400 MHz, DMSO-*d*_6_) δ 10.58 (s, 1H), 9.20 (s, 1H), 8.09 (s, 1H), 7.91 (d, *J* = 6.8, 2H), 7.79 (s, 1H), 7.72 (s, 3H), 7.50 (t, *J* = 6.5, 1H), 7.33 (s, 1H), 5.76 (s, 2H), 5.24 (s, 2H), 3.47 (s, 3H), 3.21 (s, 3H). ^13^C NMR (100 MHz, DMSO-*d*_6_) δ 165.48, 154.99, 151.49, 148.44, 147.45, 144.25, 140.13, 139.01, 137.09, 130.18, 129.70, 126.41, 126.05, 120.82, 119.89, 117.49, 106.95, 55.37, 49.25, 29.94, 27.92, 21.42. HR MS (ESI) *m/z*: calcd for C_24_H_23_N_8_O_3_ [M + H]^+^ 471.1893, found 471.1906.

2-(1,3-dimethyl-2,6-dioxo-1,2,3,6-tetrahydro-7H-purin-7-yl)-N-(4-(1-(2-(trifluoromethyl)phenyl)**-**1H-1,2,3-triazol-4-yl)phenyl)acetamide (d14). ^1^H NMR (400 MHz, DMSO-*d*_6_) δ 10.59 (s, 1H), 9.28 (s, 1H), 8.09 (s, 1H), 7.87 (dd, *J*_*1*_ = 19.7, *J*_*2*_ = 7.2, 4H), 7.78–7.64 (m, 3H), 7.37 (s, 1H), 5.25 (s, 2H), 3.47 (s, 3H), 3.21 (s, 3H). ^13^C NMR (100 MHz, DMSO-*d*_6_) δ 154.99, 151.50, 144.26, 132.41, 132.32, 126.47, 119.93, 119.72, 116.31, 55.36, 49.25, 40.21, 29.94, 27.92. HR MS (ESI) *m/z*: calcd for C_24_H_20_F_3_N_8_O_3_ [M + H]^+^ 525.1610, found 525.1619.

2-(1,3-dimethyl-2,6-dioxo-1,2,3,6-tetrahydro-7H-purin-7-yl)-N-(4-(1-(2-ethylphenyl)-1H-1,2,3-triazol-4-yl)phenyl)acetamide (d15). ^1^H NMR (400 MHz, DMSO-*d*_6_) δ 10.58 (s, 1H), 8.87 (s, 1H), 8.10 (s, 1H), 7.92 (d, *J* = 8.5, 2H), 7.71 (d, *J* = 8.6, 2H), 7.58 (s, 2H), 7.42 (s, 2H), 5.25 (s, 2H), 3.47 (s, 3H), 3.21 (s, 3H), 2.52 (s, 2H), 1.06 (t, *J* = 7.5, 3H). ^13^C NMR (100 MHz, DMSO-*d*_6_) δ 144.25, 130.69, 130.35, 127.45, 126.87, 126.40, 123.28, 119.83, 49.24, 29.94, 27.93, 24.27, 15.36. HR MS (ESI) *m/z*: calcd for C_25_H_25_N_8_O_3_ [M + H]^+^ 485.2050, found 485.2060.

2-(1,3-dimethyl-2,6-dioxo-1,2,3,6-tetrahydro-7H-purin-7-yl)-N-(4-(1-mesityl-1H-1,2,3-triazol-4-yl)phenyl)acetamide (d16). ^1^H NMR (400 MHz, DMSO-*d*_6_) δ 10.57 (s, 1H), 8.72 (s, 1H), 8.09 (s, 1H), 7.90 (d, *J* = 8.5, 2H), 7.70 (d, *J* = 8.5, 2H), 7.12 (s, 2H), 5.24 (s, 2H), 3.47 (s, 3H), 3.21 (s, 3H), 2.34 (s, 3H), 1.94 (s, 6H). ^13^C NMR (100 MHz, DMSO-*d*_6_) δ 165.45, 155.01, 151.50, 148.44, 144.28, 140.03, 138.88, 134.95, 126.37, 126.29, 123.33, 119.82, 49.23, 40.41, 29.95, 27.94, 17.36. HR MS (ESI) *m/z*: calcd for C_26_H_27_N_8_O_3_ [M + H]^+^ 499.2206, found 499.2216.

N-(4-(1-(2,5-bis(trifluoromethyl)phenyl)**-**1H-1,2,3-triazol-4-yl)phenyl)**-**2-(1,3-dimethyl-2,6-dioxo-1,2,3,6-tetrahydro-7H-purin-7-yl)acetamide (d17). ^1^H NMR (400 MHz, DMSO-*d*_6_) δ 10.57 (s, 1H), 9.02 (s, 1H), 8.40 (s, 1H), 8.30 (q, *J* = 8.4, 2H), 8.10–8.07 (m, 1H), 7.91 (d, *J* = 7.4, 2H), 7.72 (d, *J* = 7.5, 2H), 5.24 (s, 2H), 3.49–3.46 (m, 3H), 3.23–3.20 (m, 3H). ^13^C NMR (100 MHz, DMSO-*d*_6_) *δ* 165.52, 155.01, 151.51, 148.45, 146.87, 144.27, 139.18, 129.79, 127.10, 126.52, 125.55, 119.93, 106.95, 49.25, 29.96, 27.94. HR MS (ESI) *m/z*: calcd for C_25_H_19_F_6_N_8_O_3_ [M + H]^+^ 593.1484, found 593.1491.

2-(1,3-dimethyl-2,6-dioxo-1,2,3,6-tetrahydro-7H-purin-7-yl)-N-(4-(1-(3-fluorophenyl)-1H-1,2,3-triazol-4-yl)phenyl)acetamide (d18). ^1^H NMR (400 MHz, DMSO-*d*_6_) δ 10.59 (s, 1H), 9.28 (s, 1H), 8.09 (s, 1H), 7.87 (d, *J* = 12.5, 4H), 7.72 (s, 3H), 7.37 (s, 1H), 5.25 (s, 2H), 3.47 (s, 3H), 3.21 (s, 3H). ^13^C NMR (100 MHz, DMSO-*d*_6_) δ 165.52, 155.00, 151.50, 148.45, 147.65, 144.27, 139.15, 132.43, 132.34, 126.48, 125.78, 119.94, 119.74, 116.33, 115.75, 108.03, 107.76, 106.95, 49.25, 29.95, 27.93. HR MS (ESI) *m/z*: calcd for C_23_H_20_FN_8_O_3_ [M + H]^+^ 475.1642, found 475.1641.

N-(4-(1-(2-chlorophenyl)-1H-1,2,3-triazol-4-yl)phenyl)**-**2-(1,3-dimethyl-2,6-dioxo-1,2,3,6-tetrahydro-7H-purin-7-yl)acetamide (d19). ^1^H NMR (400 MHz, DMSO-*d*_6_) δ 10.58 (s, 1H), 8.97 (s, 1H), 8.09 (s, 1H), 7.91 (d, *J* = 8.4, 2H), 7.79 (dd, *J* = 15.4, 7.6, 2H), 7.71 (d, *J* = 8.4, 2H), 7.67–7.59 (m, 2H), 5.24 (s, 2H), 3.47 (s, 3H), 3.21 (s, 3H). ^13^C NMR (100 MHz, DMSO-*d*_6_) δ 151.52, 144.27, 126.45, 122.85, 122.77, 119.94, 119.86, 117.37, 117.14, 49.25, 40.44, 29.95, 27.94. HR MS (ESI) *m/z*: calcd for C_23_H_20_ClN_8_O_3_ [M + H]^+^ 491.1347, found 491.1354.

N-(4-(1-(3-bromophenyl)-1H-1,2,3-triazol-4-yl)phenyl)**-**2-(1,3-dimethyl-2,6-dioxo-1,2,3,6-tetrahydro-7H-purin-7-yl)acetamide (d20). ^1^H NMR (400 MHz, DMSO-*d*_6_) δ 10.59 (s, 1H), 9.31 (s, 1H), 8.20 (s, 1H), 8.09 (d, *J* = 4.1, 1H), 8.00 (t, *J* = 5.5, 1H), 7.89 (t, *J* = 6.1, 2H), 7.72 (dd, *J*_*1*_ = 8.3, *J*_*2*_ = 3.8, 3H), 7.59 (s, 1H), 5.24 (s, 2H), 3.47 (s, 3H), 3.21 (s, 3H). ^13^C NMR (100 MHz, DMSO-*d*_6_) δ 165.51, 154.99, 151.49, 148.44, 147.65, 144.25, 139.14, 138.23, 132.36, 131.83, 126.45, 125.79, 122.95, 119.93, 119.72, 119.34, 106.95, 49.25, 40.21, 29.95, 27.93. HR MS (ESI) *m/z*: calcd for C_23_H_20_BrN_8_O_3_ [M + H]^+^ 535.0842, found 535.0840.

N-(4-(1-(3,5-bis(trifluoromethyl)phenyl)**-**1H-1,2,3-triazol-4-yl)phenyl)**-**2-(1,3-dimethyl-2,6-dioxo-1,2,3,6-tetrahydro-7H-purin-7-yl)acetamide (d21). ^1^H NMR (400 MHz, DMSO-*d*_6_) δ 10.60 (s, 1H), 9.55 (s, 1H), 8.67 (s, 2H), 8.28 (s, 1H), 8.09 (s, 1H), 7.90 (d, *J* = 7.4, 2H), 7.74 (d, *J* = 7.6, 2H), 5.25 (s, 2H), 3.47 (s, 3H), 3.21 (s, 3H). ^13^C NMR (100 MHz, DMSO-*d*_6_) δ 165.54, 155.00, 151.50, 148.46, 147.99, 144.25, 139.31, 132.55, 132.21, 126.49, 125.48, 124.63, 120.95, 120.18, 119.99, 106.96, 55.34, 49.26, 29.93, 27.91. HR MS (ESI) *m/z*: calcd for C_25_H_19_F_6_N_8_O_3_ [M + H]^+^ 593.1484, found 593.1487.

2-(1,3-dimethyl-2,6-dioxo-1,2,3,6-tetrahydro-7H-purin-7-yl)-N-(4-(1-(2-iodophenyl)-1H-1,2,3-triazol-4-yl)phenyl)acetamide (d22). ^1^H NMR (400 MHz, DMSO-*d*_6_) δ 10.58 (s, 1H), 8.90 (s, 1H), 8.13–8.09 (m, 2H), 7.91 (d, *J* = 8.5, 2H), 7.71 (d, *J* = 8.6, 2H), 7.64 (d, *J* = 4.2, 2H), 7.39 (dt, *J*_*1*_ = 8.6, *J*_*2*_ = 4.5, 1H), 5.24 (s, 2H), 3.47 (s, 3H), 3.21 (s, 3H). ^13^C NMR (100 MHz, DMSO-*d*_6_) δ 165.47, 155.00, 151.50, 148.45, 146.68, 144.27, 140.33, 140.23, 138.99, 132.46, 129.92, 128.50, 126.40, 126.03, 123.38, 119.88, 106.95, 96.39, 49.25, 49.07, 40.20, 29.96, 27.95. HR MS (ESI) *m/z*: calcd for C_23_H_20_IN_8_O_3_ [M + H]^+^ 583.0703, found 583.0704.

N-(4-(1-(3-chlorophenyl)-1H-1,2,3-triazol-4-yl)phenyl)**-**2-(1,3-dimethyl-2,6-dioxo-1,2,3,6-tetrahydro-7H-purin-7-yl)acetamide (d23). ^1^H NMR (400 MHz, DMSO-*d*_6_) δ 10.59 (s, 1H), 9.31 (s, 1H), 8.09 (d, *J* = 5.7, 2H), 7.97 (d, *J* = 8.2, 1H), 7.90 (d, *J* = 8.4, 2H), 7.69 (dd, *J*_*1*_ = 24.6, *J*_*2*_ = 8.2, 3H), 7.59 (d, *J* = 8.0, 1H), 5.24 (s, 2H), 3.47 (s, 3H), 3.21 (s, 3H). ^13^C NMR (100 MHz, DMSO-*d*_6_) δ 165.51, 154.99, 151.50, 148.45, 147.66, 144.26, 139.15, 138.16, 134.71, 132.16, 128.93, 126.46, 125.78, 120.19, 119.93, 119.75, 118.98, 106.95, 49.25, 29.95, 27.94. HR MS (ESI) *m/z*: calcd for C_23_H_20_ClN_8_O_3_ [M + H]^+^ 491.1347, found 491.1348.

N-(4-(1-(4-chlorophenyl)-1H-1,2,3-triazol-4-yl)phenyl)**-**2-(1,3-dimethyl-2,6-dioxo-1,2,3,6-tetrahydro-7H-purin-7-yl)acetamide (d24). ^1^H NMR (400 MHz, DMSO-*d*_6_) δ 10.56 (s, 1H), 9.25 (s, 1H), 8.09 (s, 1H), 7.98 (d, *J* = 8.8, 2H), 7.89 (d, *J* = 8.6, 2H), 7.71 (d, *J* = 8.8, 4H), 5.24 (s, 2H), 3.47 (s, 3H), 3.21 (s, 3H). ^13^C NMR (100 MHz, DMSO-*d*_6_) δ 165.51, 155.00, 151.50, 148.45, 147.67, 144.27, 139.12, 135.92, 133.39, 130.40, 126.47, 125.84, 122.09, 119.91, 119.66, 106.95, 49.25, 29.96, 27.94. HR MS (ESI) *m/z*: calcd for C_23_H_20_ClN_8_O_3_ [M + H]^+^ 491.1347, found 491.1351.

2-(1,3-dimethyl-2,6-dioxo-1,2,3,6-tetrahydro-7H-purin-7-yl)-N-(4-(1-(3-methoxyphenyl)-1H-1,2,3-triazol-4-yl)phenyl)acetamide (d25). ^1^H NMR (400 MHz, DMSO-*d*_6_) δ 10.50 (s, 1H), 8.54 (s, 1H), 8.08 (s, 1H), 7.80 (d, *J* = 8.7, 2H), 7.64 (d, *J* = 8.7, 2H), 7.30 (t, *J* = 7.9, 1H), 6.91 (d, *J* = 23.8, 3H), 5.22 (s, 2H), 3.75 (s, 3H), 3.46 (s, 3H), 3.20 (s, 3H). ^13^C NMR (100 MHz, DMSO-*d*_6_) δ 165.40, 154.99, 130.43, 126.25, 121.51, 120.46, 119.84, 114.22, 113.98, 55.60, 53.42, 40.24, 29.94. HR MS (ESI) *m/z*: calcd for C_24_H_23_N_8_O_4_ [M + H]^+^ 487.1842, found 487.1771.

2-(1,3-dimethyl-2,6-dioxo-1,2,3,6-tetrahydro-7H-purin-7-yl)-N-(4-(1-(2-methoxyphenyl)-1H-1,2,3-triazol-4-yl)phenyl)acetamide (d26). ^1^H NMR (400 MHz, DMSO-*d*_6_) δ 10.56 (s, 1H), 8.84 (s, 1H), 8.09 (s, 1H), 7.91 (d, *J* = 7.1, 2H), 7.74–7.64 (m, 3H), 7.55 (s, 1H), 7.34 (d, *J* = 7.8, 1H), 7.17 (s, 1H), 5.24 (s, 2H), 3.88 (s, 3H), 3.47 (s, 3H), 3.21 (s, 3H). ^13^C NMR (100 MHz, DMSO-*d*_6_) δ 131.28, 126.41, 126.33, 126.23, 123.29, 121.33, 119.85, 113.46, 56.60, 49.24, 40.22, 29.93, 27.92. HR MS (ESI) *m/z*: calcd for C_24_H_23_N_8_O_4_ [M + H]^+^ 487.1842, found 487.1854.

2-(1,3-dimethyl-2,6-dioxo-1,2,3,6-tetrahydro-7H-purin-7-yl)-N-(4-(1-(4-fluorophenyl)-1H-1,2,3-triazol-4-yl)phenyl)acetamide (d27). ^1^H NMR (400 MHz, DMSO-*d*_6_) δ 10.56 (s, 1H), 9.20 (s, 1H), 8.09 (s, 1H), 7.99 (d, *J* = 12.4, 2H), 7.89 (d, *J* = 8.1, 2H), 7.71 (d, *J* = 8.2, 2H), 7.49 (t, *J* = 8.5, 2H), 5.24 (s, 2H), 3.47 (s, 3H), 3.21 (s, 3H). ^13^C NMR (100 MHz, DMSO-*d*_6_) δ 151.52, 144.27, 126.45, 122.85, 122.77, 119.94, 119.86, 117.37, 117.14, 49.25, 40.44, 29.95, 27.94. HR MS (ESI) *m/z*: calcd for C_23_H_20_FN_8_O_3_ [M + H]^+^ 475.1642, found 475.1648.

N-(4-(1-(2-bromophenyl)-1H-1,2,3-triazol-4-yl)phenyl)**-**2-(1,3-dimethyl-2,6-dioxo-1,2,3,6-tetrahydro-7H-purin-7-yl)acetamide (d28). ^1^H NMR (400 MHz, DMSO-*d*_6_) δ 10.56 (s, 1H), 8.94 (s, 1H), 8.09 (s, 1H), 7.93 (d, *J* = 20.7, 3H), 7.77–7.56 (m, 5H), 5.24 (s, 2H), 3.47 (d, *J* = 3.0, 3H), 3.21 (s, 3H). ^13^C NMR (100 MHz, DMSO-*d*_6_) δ 165.47, 155.00, 151.50, 148.45, 146.66, 144.26, 139.02, 136.72, 134.13, 132.50, 129.46, 129.17, 126.44, 125.95, 123.52, 119.92, 119.38, 49.25, 40.23, 29.95, 27.93. HR MS (ESI) *m/z*: calcd for C_23_H_20_BrN_8_O_3_ [M + H]^+^ 535.0842, found 535.0848.

2-(1,3-dimethyl-2,6-dioxo-1,2,3,6-tetrahydro-7H-purin-7-yl)-N-(4-(1-(4-(trifluoromethyl)phenyl)**-**1H-1,2,3-triazol-4-yl)phenyl)acetamide (d29). ^1^H NMR (400 MHz, DMSO-*d*_6_) δ 10.57 (s, 1H), 9.38 (s, 1H), 8.21 (d, *J* = 7.7, 2H), 8.10–8.00 (m, 3H), 7.92 (d, *J* = 7.9, 2H), 7.72 (d, *J* = 7.8, 2H), 5.24 (s, 2H), 3.47 (s, 3H), 3.21 (s, 3H). ^13^C NMR (100 MHz, DMSO-*d*_6_) δ 165.53, 155.00, 151.50, 148.45, 144.27, 139.89, 139.22, 127.79, 127.75, 126.53, 125.67,120.80, 119.93, 119.77, 106.96, 49.26, 29.96, 27.94. HR MS (ESI) *m/z*: calcd for C_24_H_20_F_3_N_8_O_3_ [M + H]^+^ 525.1610, found 25.1621.

### Bioexperiment

#### Cell Culture and Treatment

Human non-small cell lung cancer cell lines PC-9, H460, and A549 were cultured with the RIPM-1640 complete medium containing 10% FBS and 1% penicillin–streptomycin at 37°C in a 5% CO_2_ humidification environment. Other tumor cell lines A2780, LOVO, MB-231, MCF-7, OVCAR-3, and SW480 were cultured with the DMEM complete medium containing 10% FBS and 1% penicillin–streptomycin at 37°C in 5% CO_2_ humidification environment too. All compounds were dissolved in DMSO to prepare 100 mM mother liquor and then used complete the medium to prepare different working concentrations.

#### Cell Counting Kit-8 (CCK-8) for Cell Proliferation and Cytotoxicity Assays

Cells in the logarithmic growth phase were seeded into 96-well plates (2000–4,000 cells/well). 24 h after cell implantation, the cells were treated with different concentrations of the compound (1, 2, 8, 16 μM) for 72 h, and 0.1% DMSO was used as a negative control. Finally, the CCK8 reagent was added and incubated for 1–4 h at 37°C. The absorbance of each well was detected at a 450 nm wavelength by a multifunctional microplate reader (Thermo Fisher Varioskan Luk). The cell survival rate of the negative control group was regarded as 100%, and the half-maximal inhibitory concentration (IC50) of the compounds was calculated by Graph Pad Prism 8.0 software.

#### Live/Dead Cell Imaging

LIVE/DEAD cell analysis was carried out using a laser confocal fluorescence microscope using the LIVE/DEAD kit. In brief, H460 and A549 (3 × 10^3^–5 × 10^3^ cells/well) cells were seeded in 96-well plates incubating for 24 h, and then, cells were treated with various concentrations of compound d17 (5, 10, 15 μM) for 48 h and 0.1% DMSO was used as a control. After various concentrations, compound d17 cells were stained with the LIVE/DEAD Cell Imaging Kit for 15–20 min and then observed and photographed using a fluorescence microscope (LSM880 with Fast Airyscan).

#### Flow Cytometry Detection for Cell Apoptosis

The cell apoptosis assay was carried out using the Annexin V/PI apoptosis kit and flow cytometry (BD LSRFortessa^TM^ Flow Cytometer). Briefly, H460 and A549 cells in the logarithmic growth phase were seeded into 6-well plates (4.0×10^5^∼6.0×10^5^ cells/well). 24 h after cell implantation, the cells were treated with different concentrations of compound d17 (5, 10, 15 μM) for 48 h, and 0.1%DMSO was used as a negative control. All cells (including those in the supernatant) were collected after trypsin digestion and washed with PBS; then, the cells were gently resuspended with 100 μL Annexin V-FITC binding solution and then incubated with 2.5 μL Annexin V-FITC and 5 μL of propidium iodide (PI) staining solution in dark at room temperature for 20–30 min. Finally, cell apoptosis of each well was detected by flow cytometry. The percentage of apoptosis was analyzed by Flowjo software.

#### Western Blot Analysis

H460 and A549 cells in the logarithmic growth phase were seeded into 6-well plates (4.0 × 10^5^∼6.0 × 10^5^ cells/well). 24 h after cell implantation, the cells were treated with different concentrations of compound d17 (5, 10, 15 μM) for 24 h, and 0.1% DMSO was used as a negative control. The supernatant was discarded, and the cells were collected by trypsin digestion and washed once with PBS. Then, the cells were lysed on ice with 100 μL of RIPA lysis buffer containing protease and the phosphatase inhibitor for 30 min. Finally, the total protein extract was obtained by centrifugation at 12,000 RPM at 4 degrees for 10 min. The proteins were isolated by electrophoresis with 12.5% sodium dodecyl sulfate polyacrylamide gel. After electrophoresis, the proteins were transferred to the NC membrane and then sealed with 5% skim milk prepared by TBS-T [150 mM NaCl, 10 mM Tris (pH 7.4), and 0.1% Tween20] at room temperature for 1 h. After sealing, 1:1,000 diluted solution of anti-Bax (D2E11), anti-Bcl-2 (124), anti-Akt (PAN) (C67E7), anti-Akt1 (PhosphoS473) (EP2109Y), and the anti-β-actin (8H10D10) primary antibody was incubated overnight at 4°Cand then washed with TBS-T for 5 min (three times). Incubation was carried out with 1:2000 diluted solution of the antirabbit or antimouse secondary antibody for 1 h at room temperature, and finally, washing was carried out with TBS-T for 5 min (three times) to obtain protein strips through chemiluminescence. The protein expression level and proportion were quantitatively analyzed by ImageJ software.

### Statistical Analysis

All values are presented as means ± SD. The significant differences are determined using GraphPad Prism 8 software. The significant differences between the two groups are confirmed using Student’s t-test. All experiments are considered to be statistically significant using one-way ANOVA, followed by Tukey’s post test (significant difference at *p* < 0.05).

## Data Availability

The original contributions presented in the study are included in the article/Supplementary Material, and further inquiries can be directed to the corresponding author/s.
