# Supplementary figures and images for: Discovery of a Series of Theophylline Derivatives Containing 1,2,3-Triazole for Treatment of Non-Small Cell Lung Cancer
Source: Front Pharmacol. 2021 Oct 26;12:753676. doi: 10.3389/fphar.2021.753676 (PMC8576520; doi:10.3389/fphar.2021.753676)

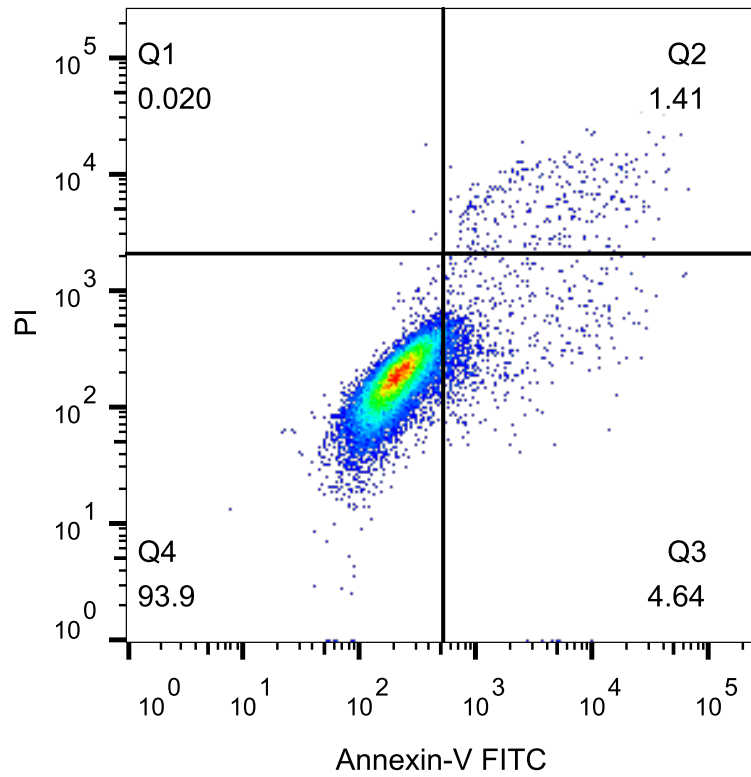

2021 5 13\_d17 5\_002.fcs

Lymphocytes

20000

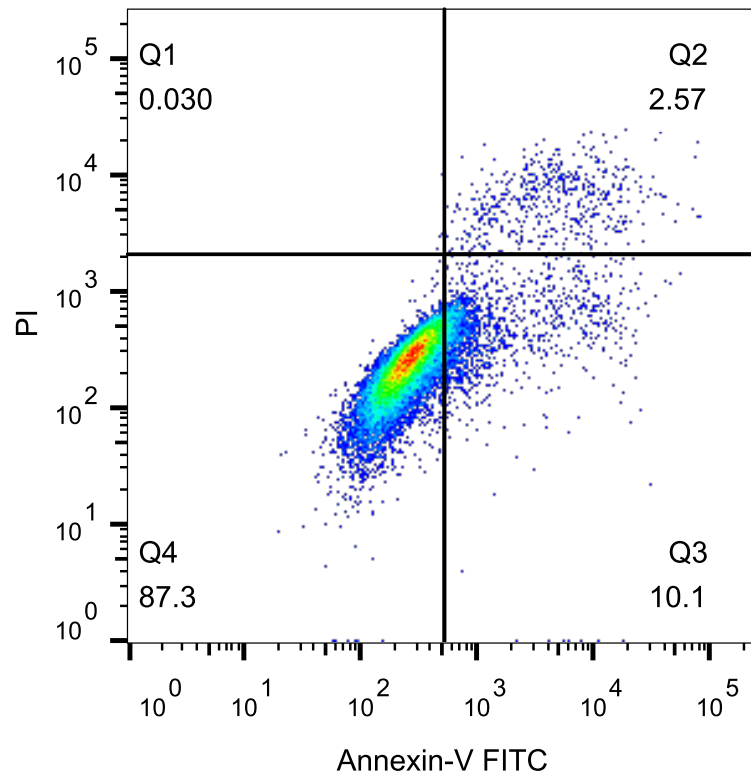

2021 5 13\_d17 10\_003.fcs  
Lymphocytes  
19966

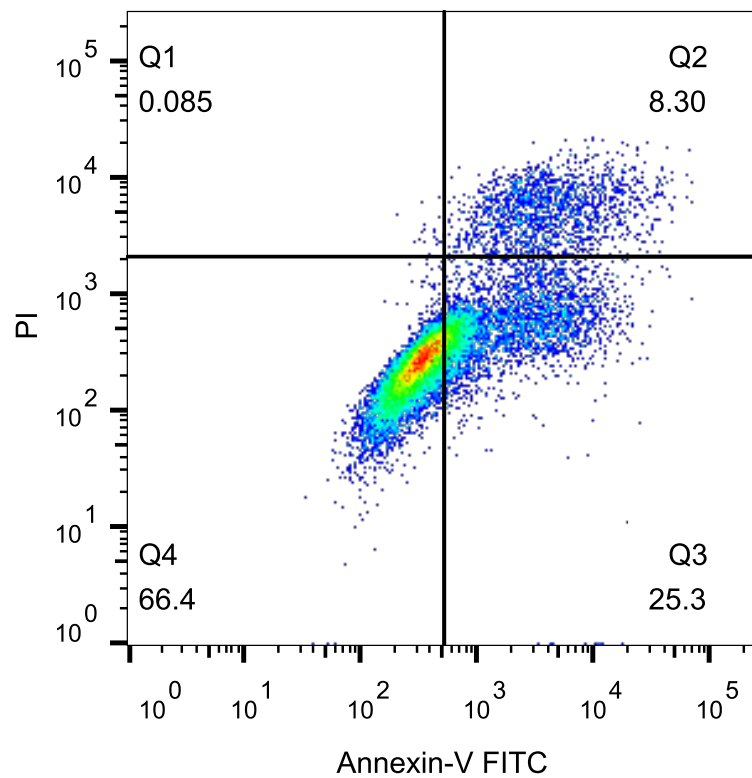

2021 5 13\_d17 15\_004.fcs

Lymphocytes

19918

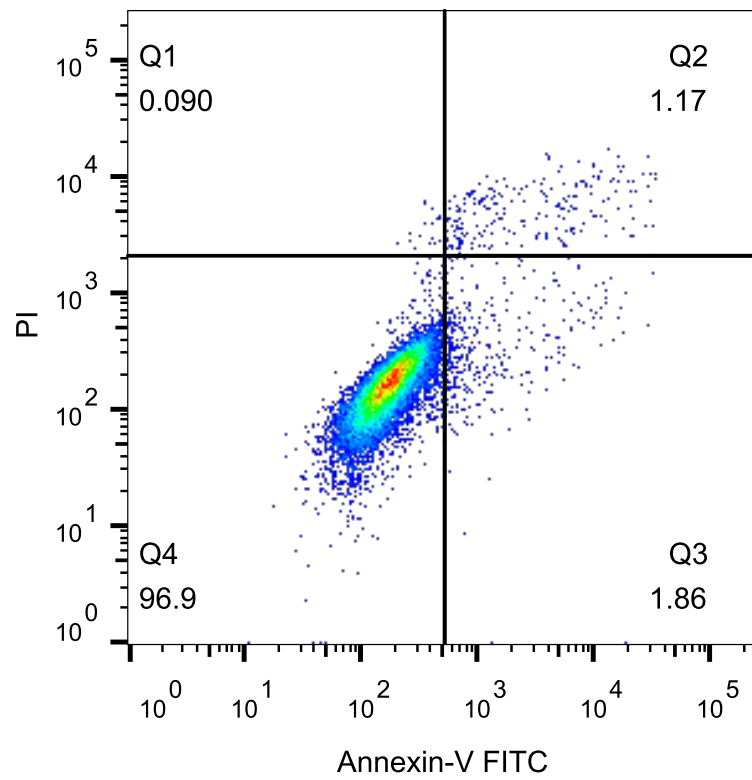

2021 5 13\_d170\_001.fcs  
Lymphocytes  
20000

Supplement: Supplementary file 1 [file DataSheet3.ZIP › D17┴≈╩╜/1/A549/A549 d17 48h-Layout-Batch/A549 d17 48h-Layout-Batch.pdf]

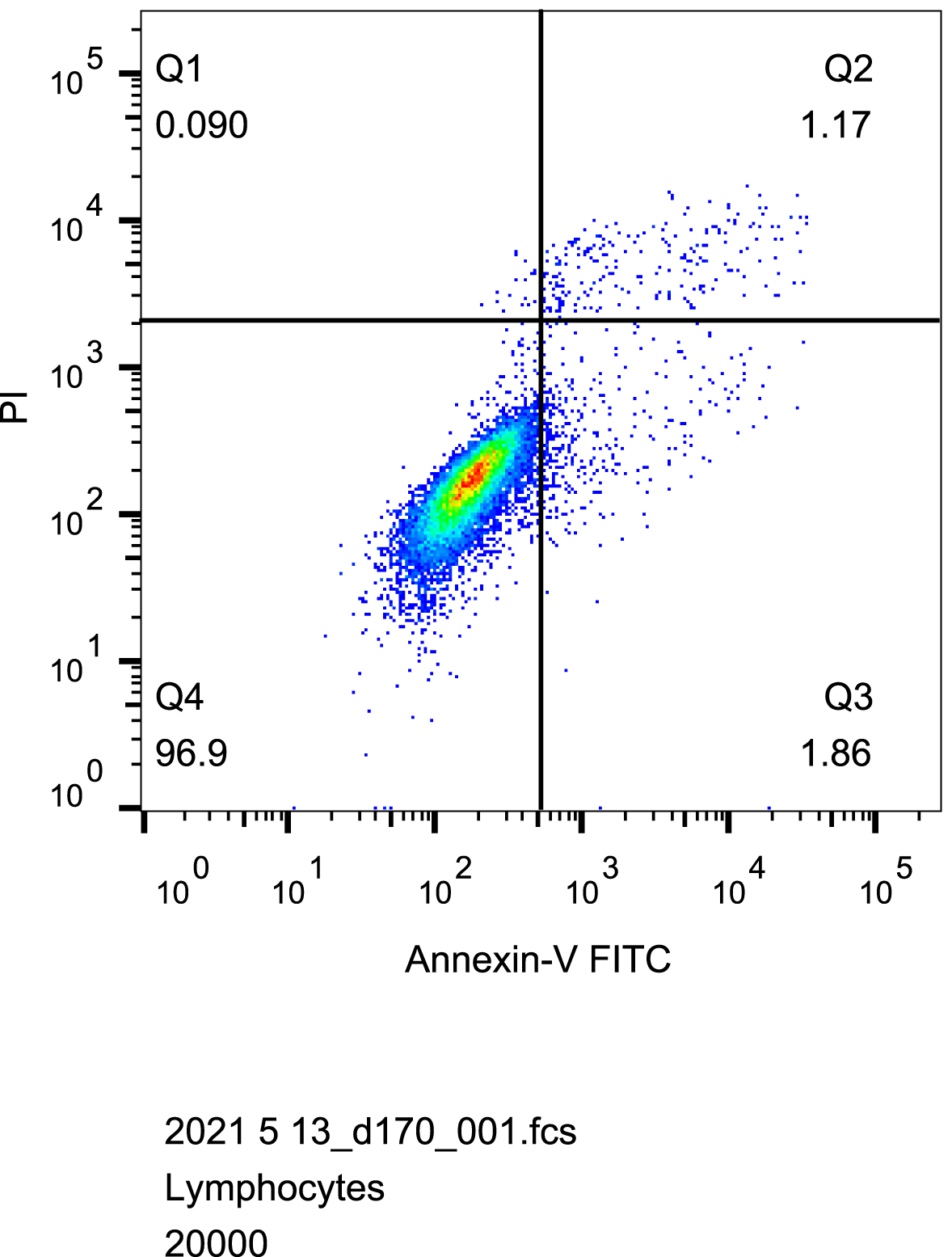

Supplement: Supplementary file 1 [file DataSheet3.ZIP › D17┴≈╩╜/1/A549/A549 d17 48h-Layout-Batch/A549 d17-0a╠M.tif]

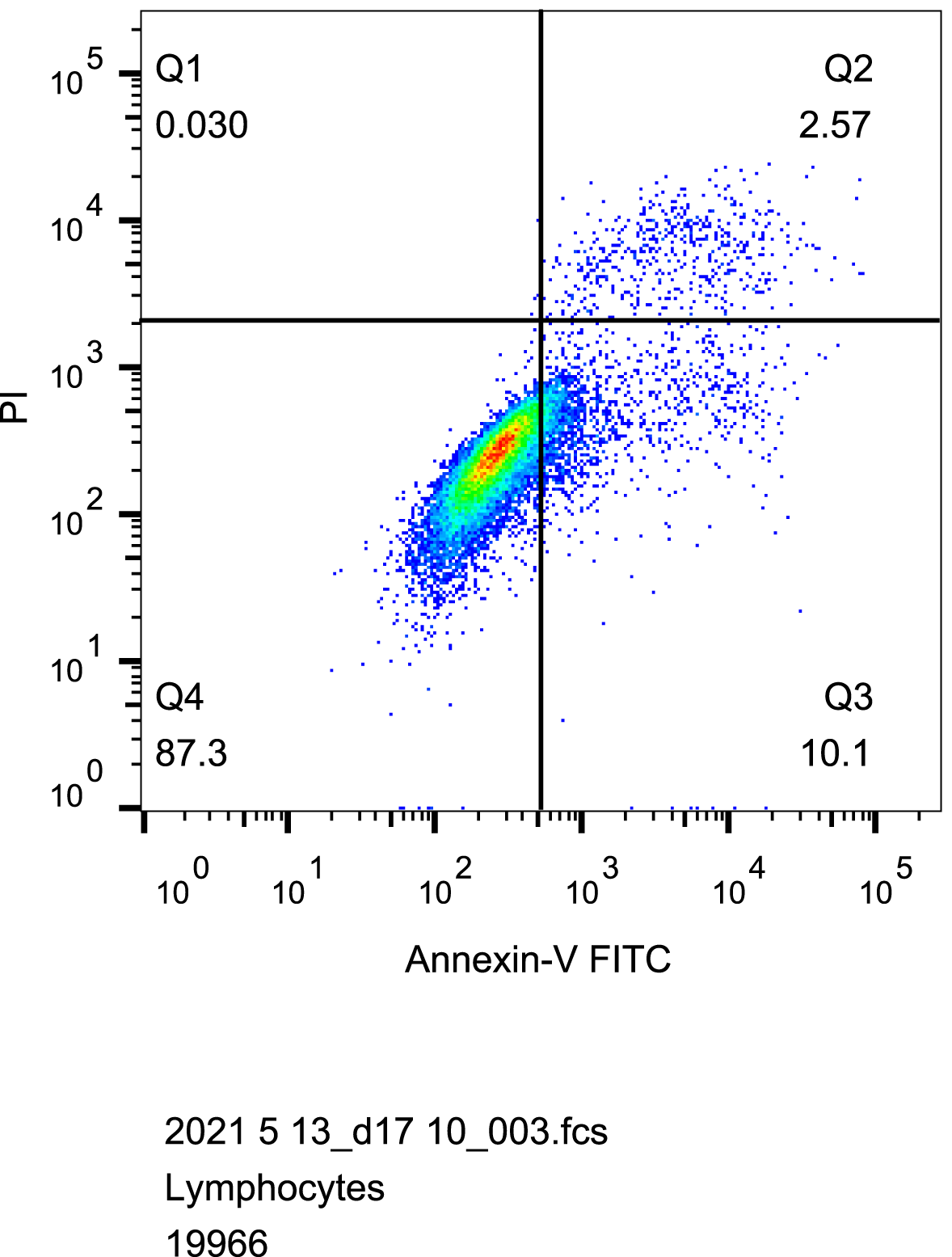

Supplement: Supplementary file 1 [file DataSheet3.ZIP › D17┴≈╩╜/1/A549/A549 d17 48h-Layout-Batch/A549 d17-10a╠M.tif]

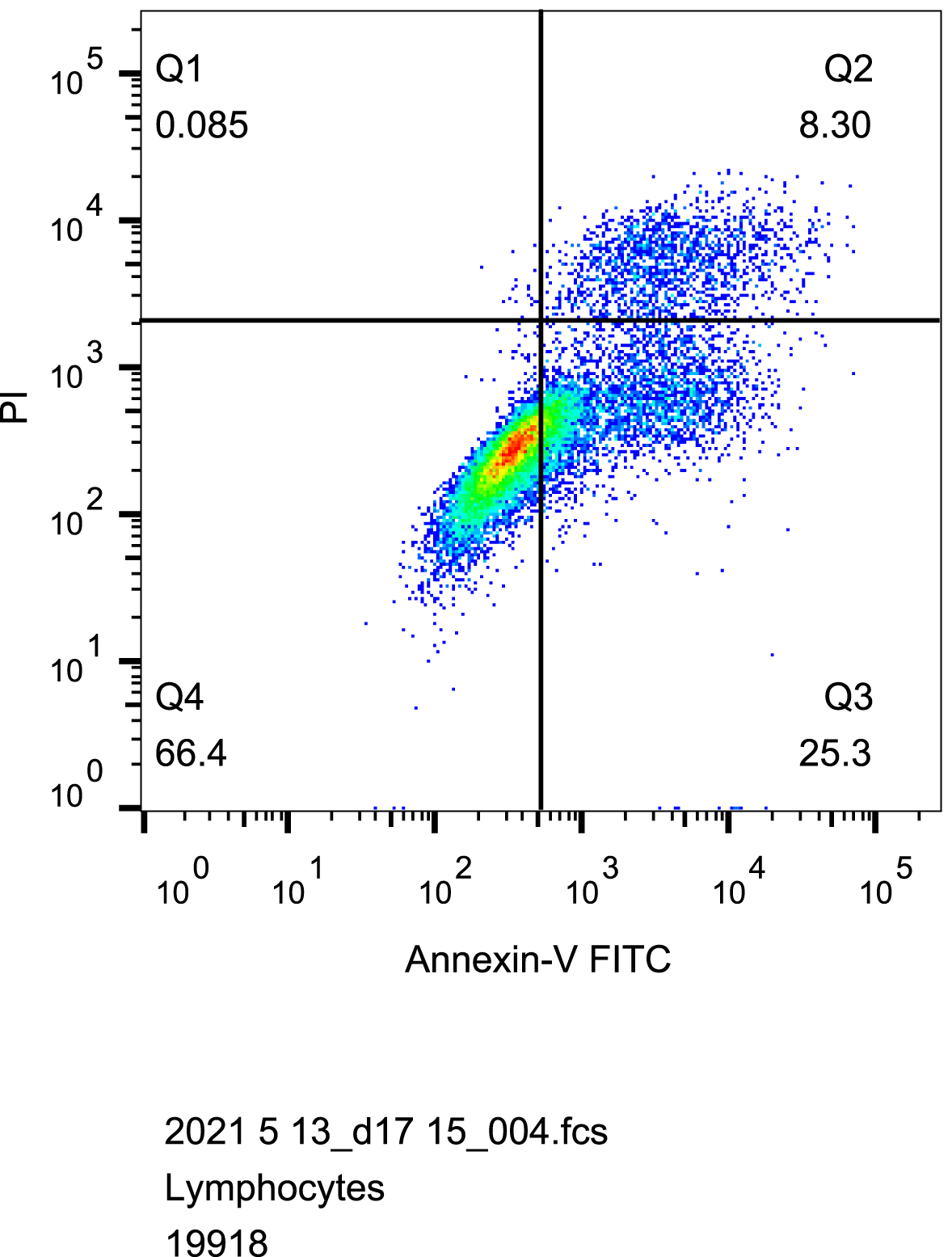

Supplement: Supplementary file 1 [file DataSheet3.ZIP › D17┴≈╩╜/1/A549/A549 d17 48h-Layout-Batch/A549 d17-15a╠M.tif]

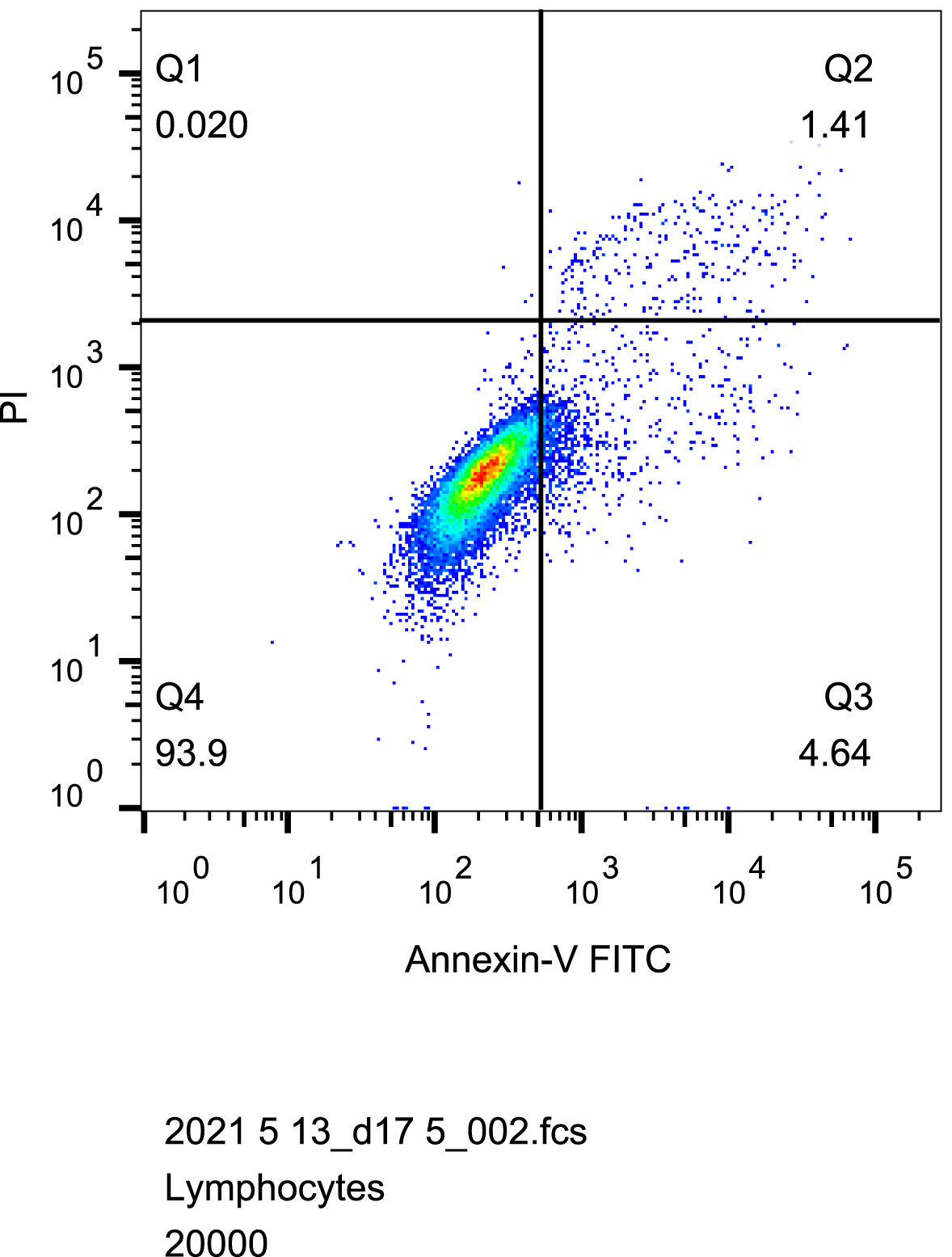

Supplement: Supplementary file 1 [file DataSheet3.ZIP › D17┴≈╩╜/1/A549/A549 d17 48h-Layout-Batch/A549 d17-5a╠M.tif]

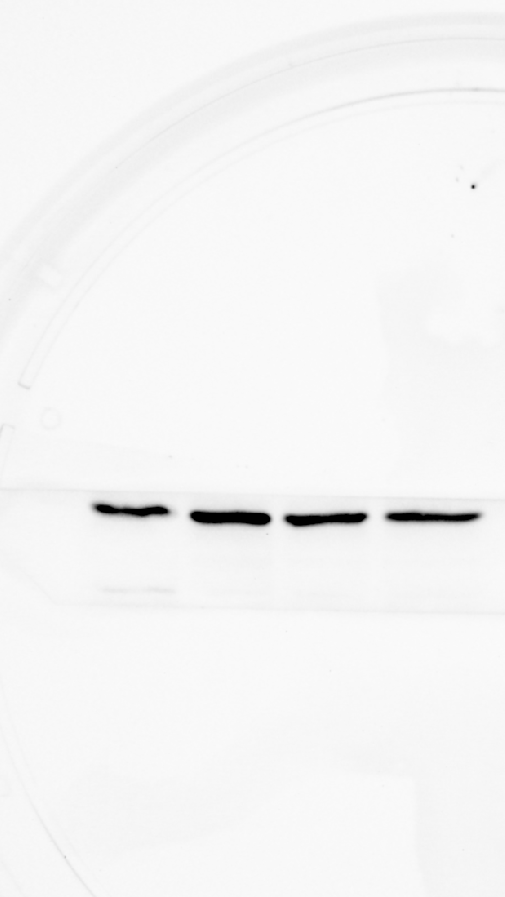

Supplement: Supplementary file 4 [file DataSheet2.ZIP › D17 WB╘¡╩╝╩2╛▌/1/Bax@Bcl-2/Bax.tiff]

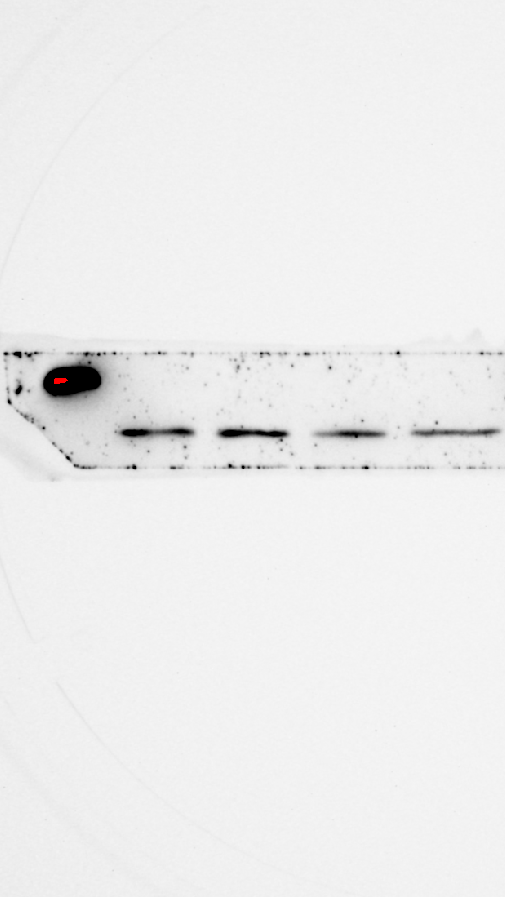

Supplement: Supplementary file 4 [file DataSheet2.ZIP › D17 WB╘¡╩╝╩2╛▌/1/Bax@Bcl-2/Bcl-2.tiff]

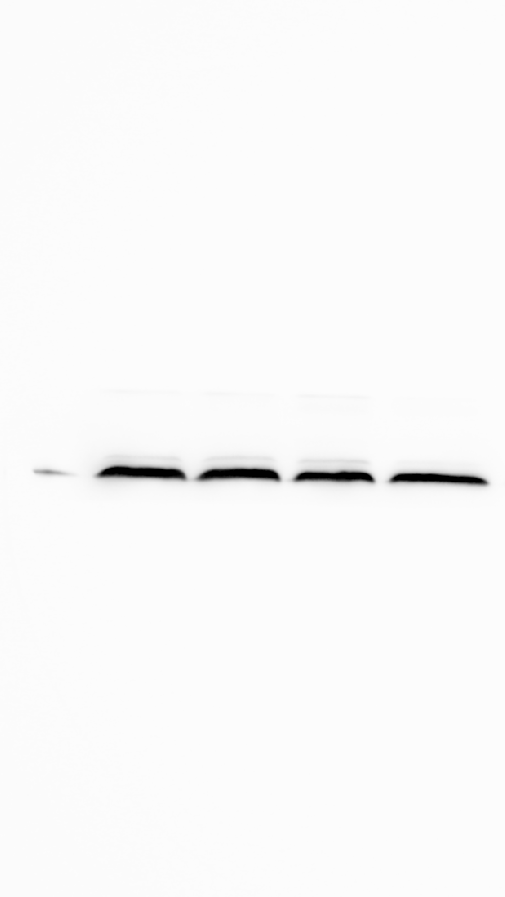

Supplement: Supplementary file 4 [file DataSheet2.ZIP › D17 WB╘¡╩╝╩2╛▌/1/Bax@Bcl-2/a┬-actin.tiff]

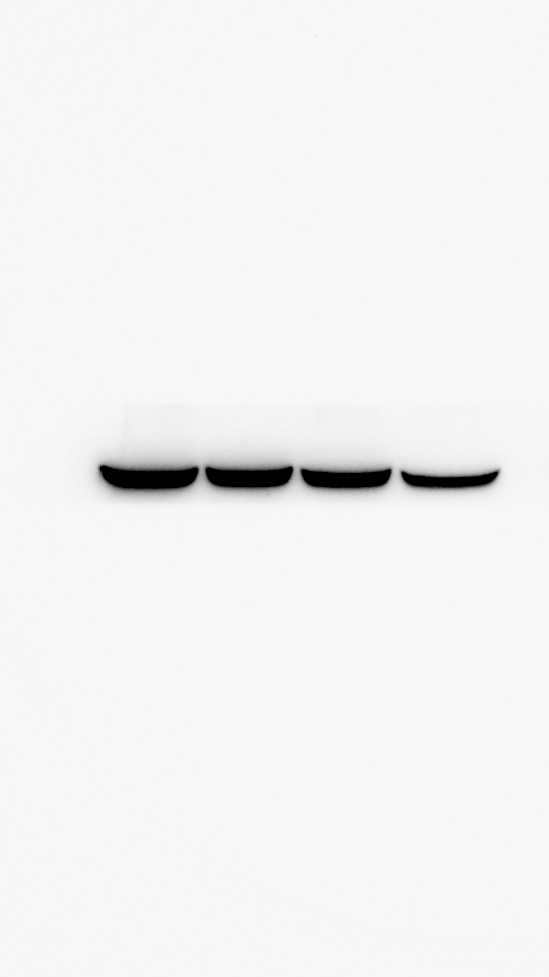

Supplement: Supplementary file 4 [file DataSheet2.ZIP › D17 WB╘¡╩╝╩2╛▌/1/p-akt@akt/akt.tiff]

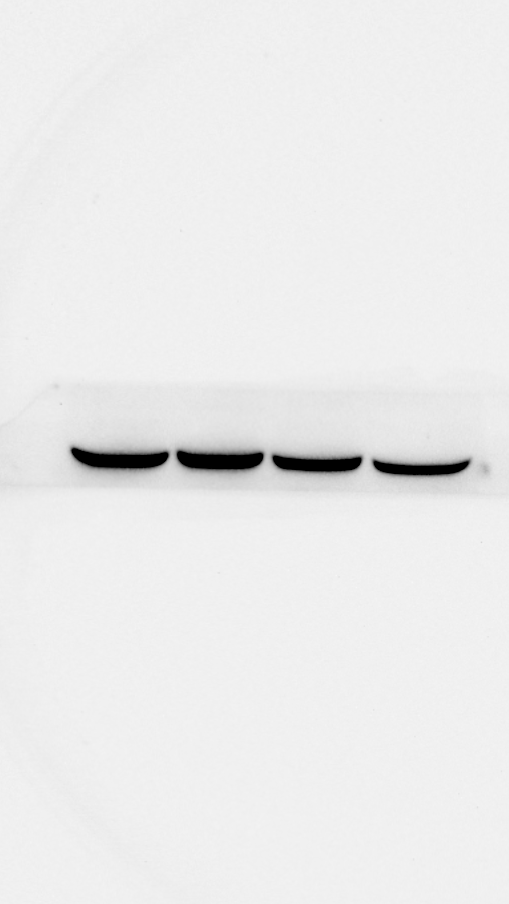

Supplement: Supplementary file 4 [file DataSheet2.ZIP › D17 WB╘¡╩╝╩2╛▌/1/p-akt@akt/p-akt.tiff]

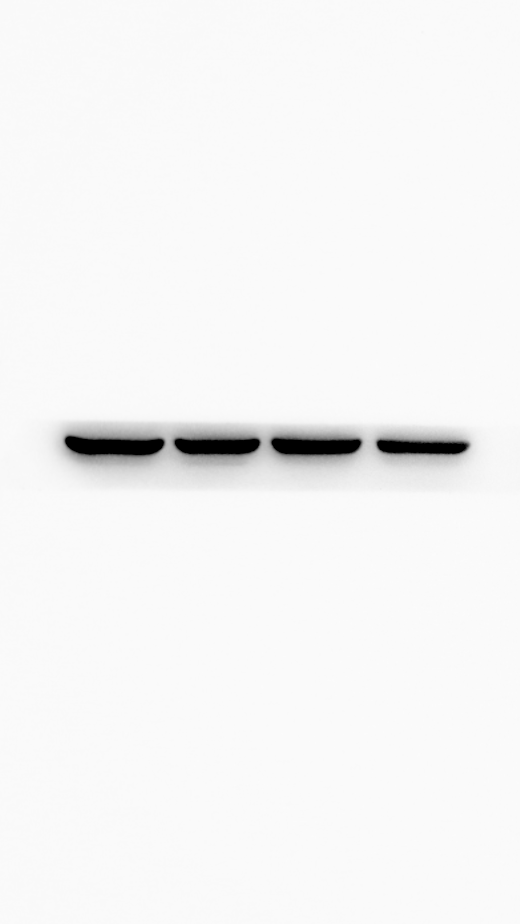

Supplement: Supplementary file 4 [file DataSheet2.ZIP › D17 WB╘¡╩╝╩2╛▌/1/p-akt@akt/a┬-actin.tiff]

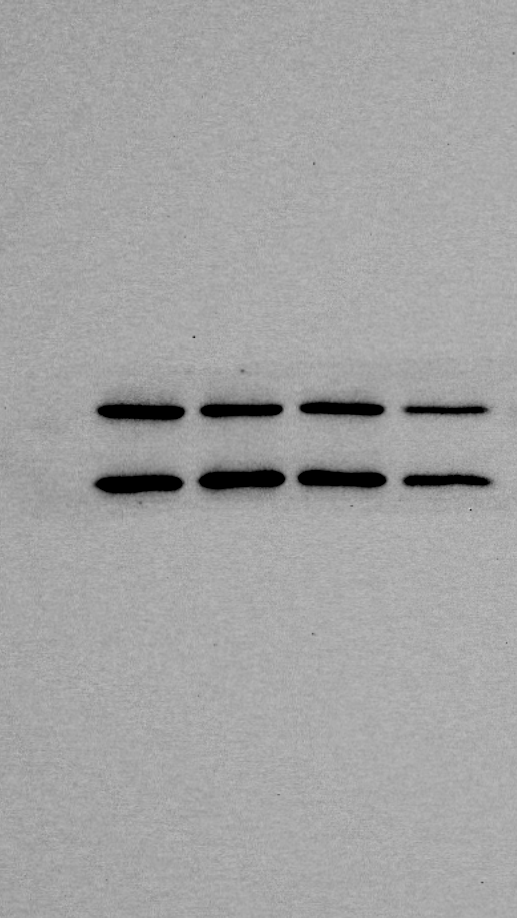

Supplement: Supplementary file 4 [file DataSheet2.ZIP › D17 WB╘¡╩╝╩2╛▌/2/Bax@Bcl-2/Bax.tiff]

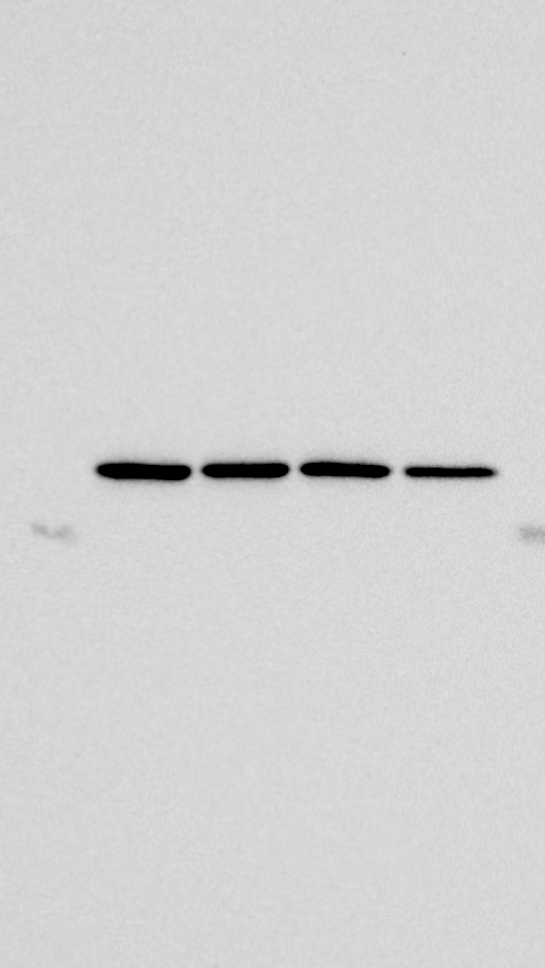

Supplement: Supplementary file 4 [file DataSheet2.ZIP › D17 WB╘¡╩╝╩2╛▌/2/Bax@Bcl-2/Bcl-2.tiff]

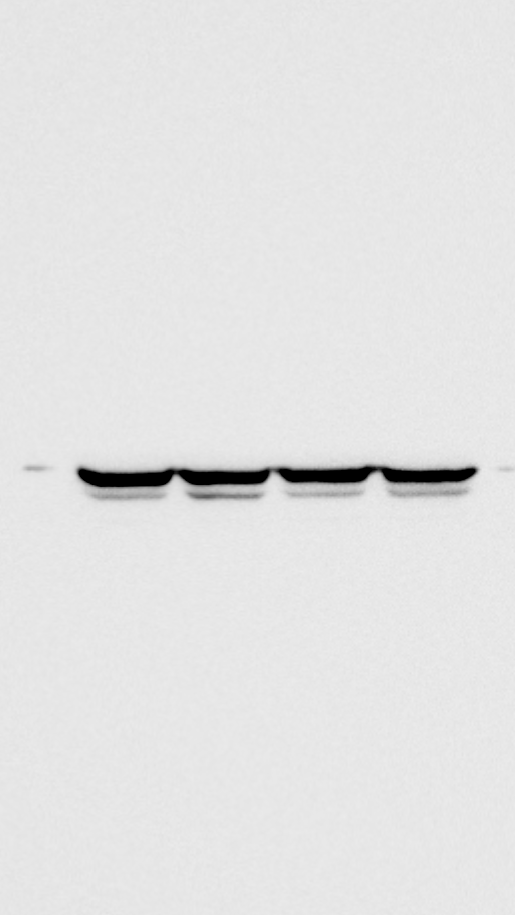

Supplement: Supplementary file 4 [file DataSheet2.ZIP › D17 WB╘¡╩╝╩2╛▌/2/Bax@Bcl-2/a┬-actin.tiff]

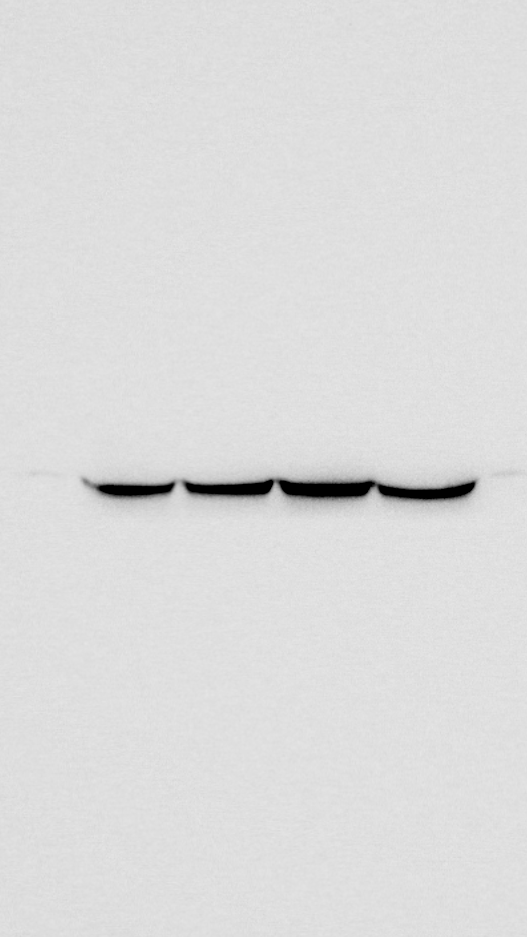

Supplement: Supplementary file 4 [file DataSheet2.ZIP › D17 WB╘¡╩╝╩2╛▌/2/p-akt@akt/Akt.tiff]

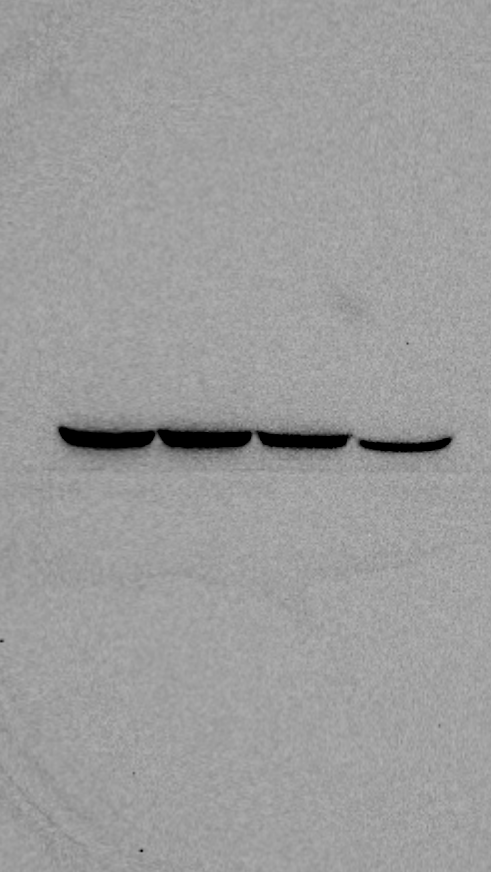

Supplement: Supplementary file 4 [file DataSheet2.ZIP › D17 WB╘¡╩╝╩2╛▌/2/p-akt@akt/p-Akt.tiff]

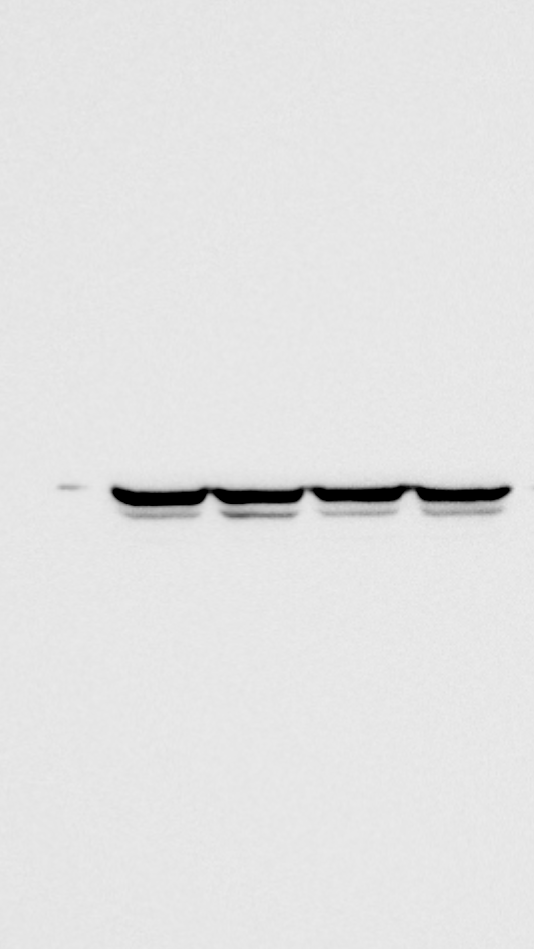

Supplement: Supplementary file 4 [file DataSheet2.ZIP › D17 WB╘¡╩╝╩2╛▌/2/p-akt@akt/a┬-actin.tiff]

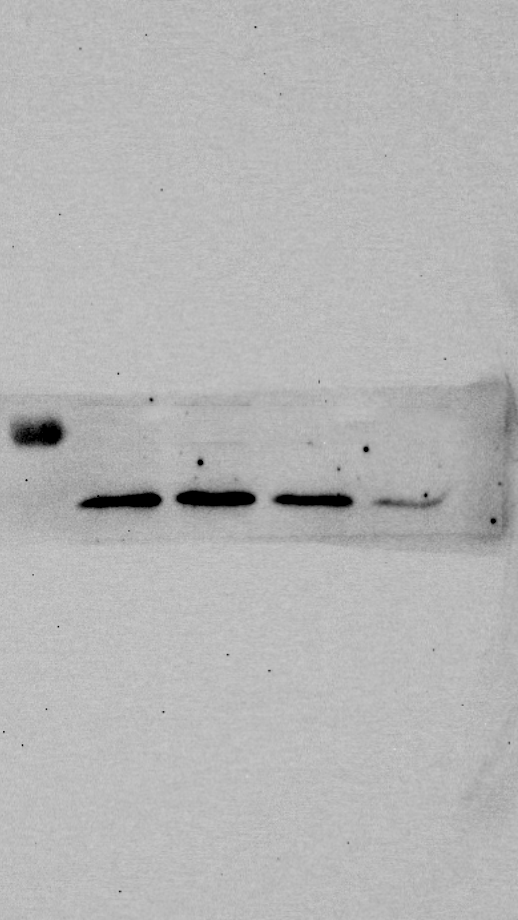

Supplement: Supplementary file 4 [file DataSheet2.ZIP › D17 WB╘¡╩╝╩2╛▌/3/Bax@Bcl-2/Bax.tiff]

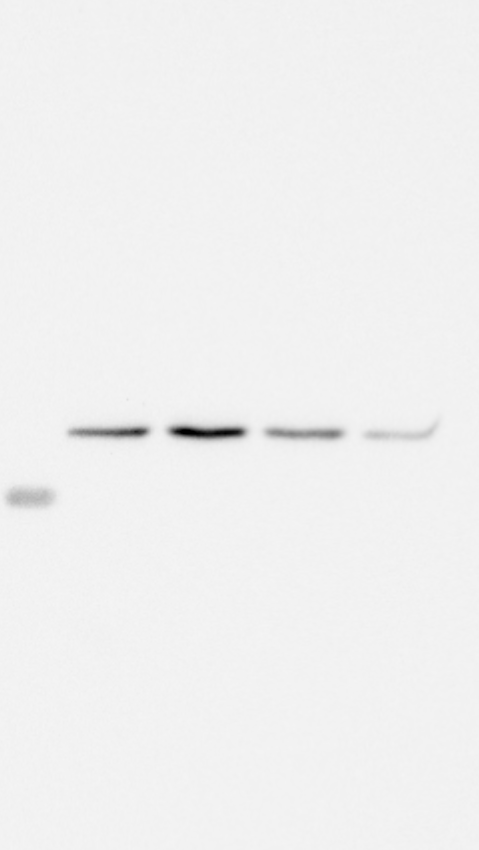

Supplement: Supplementary file 4 [file DataSheet2.ZIP › D17 WB╘¡╩╝╩2╛▌/3/Bax@Bcl-2/Bcl-2.tiff]

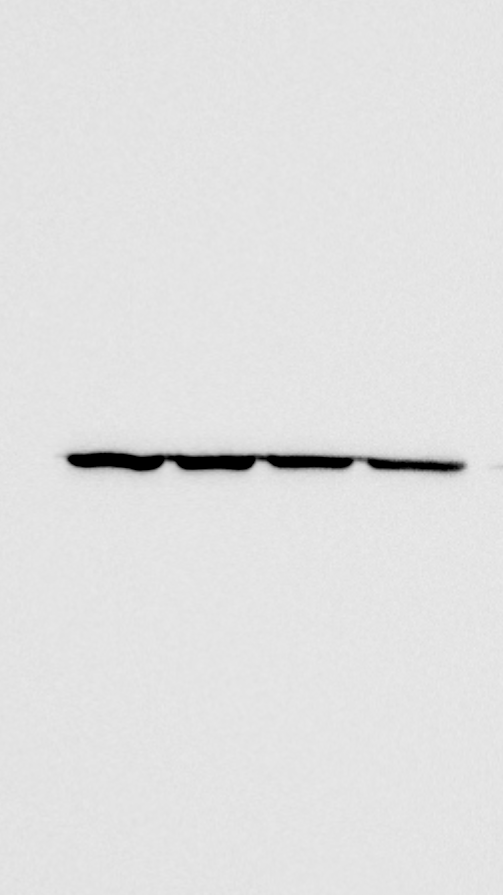

Supplement: Supplementary file 4 [file DataSheet2.ZIP › D17 WB╘¡╩╝╩2╛▌/3/Bax@Bcl-2/a┬-actin.tiff]

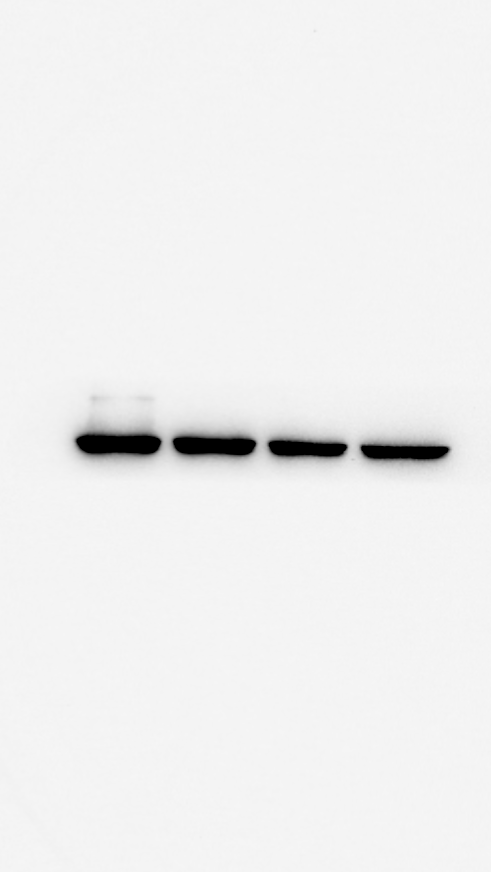

Supplement: Supplementary file 4 [file DataSheet2.ZIP › D17 WB╘¡╩╝╩2╛▌/3/p-akt@akt/Akt.tiff]

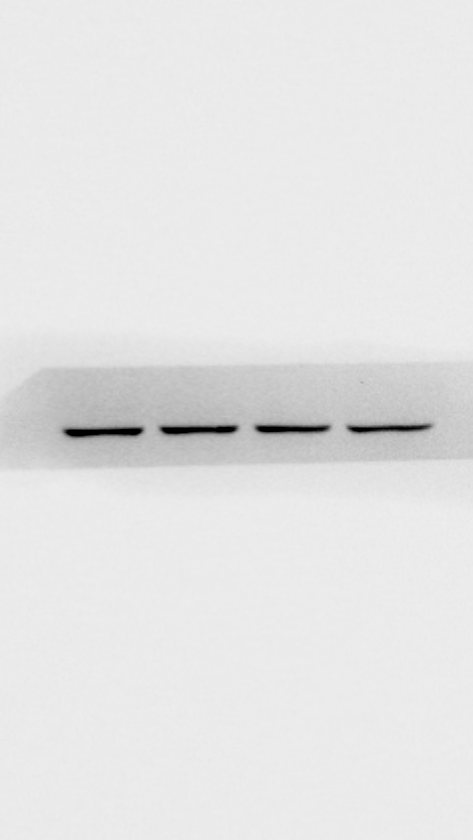

Supplement: Supplementary file 4 [file DataSheet2.ZIP › D17 WB╘¡╩╝╩2╛▌/3/p-akt@akt/p-Akt.tiff]

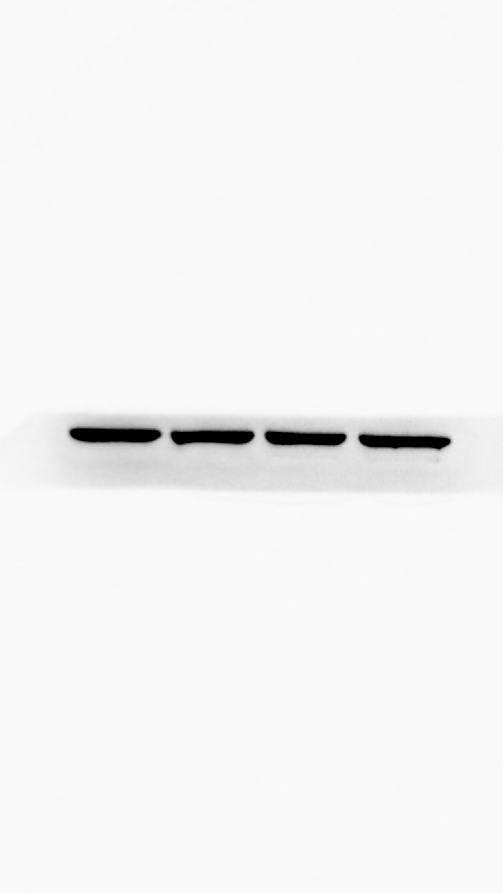

Supplement: Supplementary file 4 [file DataSheet2.ZIP › D17 WB╘¡╩╝╩2╛▌/3/p-akt@akt/a┬-actin.tiff]
