# Supplementary material for: Discovery of a Series of Theophylline Derivatives Containing 1,2,3-Triazole for Treatment of Non-Small Cell Lung Cancer
Source: Front Pharmacol. 2021 Oct 26;12:753676. doi: 10.3389/fphar.2021.753676 (PMC8576520; doi:10.3389/fphar.2021.753676)

Supplementary Information

Discovery of a series of Theophylline derivatives containing1,2,3-triazole for treatment of non-small cell lung cancer (NSCLC)

Jiahui Ye[a,#], LongfeiMao[b,#], LuoyijunXie[a,#], RongjunZhang[a], YulinLiu[a], Jianxue Yang [c,*], Qingjiao Li [a,*], MiaomiaoYuan[a,*]

[a]The Eighth Affiliated Hospital, Sun Yat-sen University, Shenzhen 518033, Guangdong, China.

[b]School of Chemistry and Chemical Engineering, Henan Normal University, Henan Engineering Research Center of Chiral Hydroxyl Pharmaceutical, Xinxiang 453007, China.

[c]Department of Neurology, The First Affiliated Hospital of Henan University of Science and Technology

***Corresponding authors:** Miaomiao Yuan, yuanmm2019@163.com;Qingjiao Li, email:liqj23@mail.sysu.edu.cn;Jianxue Yang, email: Docyjx1969@126.com.

[#]Jiahui Ye, Longfei Mao and LuoyijunXie contributed equally.

Table of Contents

Figure S1-1. ^{1}H NMR spectrum (400 MHz, DMSO-d$_6$) of compound D1 4
Figure S1-2. ^{13}C NMR spectrum (100 MHz, DMSO-d$_6$) of compound D1 5
Figure S2-1. ^{1}H NMR spectrum (400 MHz, DMSO-d$_6$) of compound D2 6
Figure S2-2. ^{13}C NMR spectrum (100 MHz, DMSO-d$_6$) of compound D2 7
Figure S3-1. ^{1}H NMR spectrum (400 MHz, DMSO-d$_6$) of compound D3 8
Figure S3-2. ^{13}C NMR spectrum (100 MHz, DMSO-d$_6$) of compound D3 9
Figure S4-1. ^{1}H NMR spectrum (400 MHz, DMSO-d$_6$) of compound D4 10
Figure S4-2. ^{13}C NMR spectrum (100 MHz, DMSO-d$_6$) of compound D4 11
Figure S5-1. ^{1}H NMR spectrum (400 MHz, DMSO-d$_6$) of compound D5 12
Figure S5-2. ^{13}C NMR spectrum (100 MHz, DMSO-d$_6$) of compound D5 13
Figure S6-1. ^{1}H NMR spectrum (400 MHz, DMSO-d$_6$) of compound D6 14
Figure S6-2. ^{13}C NMR spectrum (100 MHz, DMSO-d$_6$) of compound D6 15
Figure S7-1. ^{1}H NMR spectrum (400 MHz, DMSO-d$_6$) of compound D7 16
Figure S7-2. ^{13}C NMR spectrum (100 MHz, DMSO-d$_6$) of compound D7 17
Figure S8-1. ^{1}H NMR spectrum (400 MHz, DMSO-d$_6$) of compound D8 18
Figure S8-2. ^{13}C NMR spectrum (100 MHz, DMSO-d$_6$) of compound D8 19
Figure S9-1. ^{1}H NMR spectrum (400 MHz, DMSO-d$_6$) of compound D9 20
Figure S9-2. ^{13}C NMR spectrum (100 MHz, DMSO-d$_6$) of compound D9 21
Figure S10-1. ^{1}H NMR spectrum (400 MHz, DMSO-d$_6$) of compound D10 22
Figure S10-2. ^{13}C NMR spectrum (100 MHz, DMSO-d$_6$) of compound D10 23
Figure S11-1. ^{1}H NMR spectrum (400 MHz, DMSO-d$_6$) of compound D11 24
Figure S11-2. ^{13}C NMR spectrum (100 MHz, DMSO-d$_6$) of compound D11 25
Figure S12-1. ^{1}H NMR spectrum (400 MHz, DMSO-d$_6$) of compound D12 26
Figure S12-2. ^{12}C NMR spectrum (100 MHz, DMSO-d$_6$) of compound D12 27
Figure S13-1. ^{1}H NMR spectrum (400 MHz, DMSO-d$_6$) of compound D13 28
Figure S13-2. ^{13}C NMR spectrum (100 MHz, DMSO-d$_6$) of compound D13 29
Figure S14-1. ^{1}H NMR spectrum (400 MHz, DMSO-d$_6$) of compound D14 30
Figure S14-2. ^{13}C NMR spectrum (100 MHz, DMSO-d$_6$) of compound D14 31
Figure S15-1. ^{1}H NMR spectrum (400 MHz, DMSO-d$_6$) of compound D15 32
Figure S15-2. ^{13}C NMR spectrum (100 MHz, DMSO-d$_6$) of compound D15 33
Figure S16-1. ^{1}H NMR spectrum (400 MHz, DMSO-d$_6$) of compound D16 34
Figure S16-2. ^{13}C NMR spectrum (100 MHz, DMSO-d$_6$) of compound D16 35
Figure S17-1. ^{1}H NMR spectrum (400 MHz, DMSO-d$_6$) of compound D17 36
Figure S17-2. ^{13}C NMR spectrum (100 MHz, DMSO-d$_6$) of compound D17 37
Figure S18-1. ^{1}H NMR spectrum (400 MHz, DMSO-d$_6$) of compound D18 38
Figure S18-2. ^{13}C NMR spectrum (100 MHz, DMSO-d$_6$) of compound D18 39
Figure S19-1. ^{1}H NMR spectrum (400 MHz, DMSO-d$_6$) of compound D19 40
Figure S19-2. ^{13}C NMR spectrum (100 MHz, DMSO-d$_6$) of compound D19 41
Figure S20-1. ^{1}H NMR spectrum (400 MHz, DMSO-d$_6$) of compound D20 42
Figure S20-2. ^{13}C NMR spectrum (100 MHz, DMSO-d$_6$) of compound D20 43
Figure S21-1. ^{1}H NMR spectrum (400 MHz, DMSO-d$_6$) of compound D21 44
Figure S21-2. ^{13}C NMR spectrum (100 MHz, DMSO-d$_6$) of compound D21 45

Figure S22-1. ^{1}H NMR spectrum (400 MHz, DMSO-d_6) of compound D22 46
Figure S22-2. ^{13}C NMR spectrum (100 MHz, DMSO-d_6) of compound D22 47
Figure S23-1. ^{1}H NMR spectrum (400 MHz, DMSO-d_6) of compound D23 48
Figure S23-2. ^{13}C NMR spectrum (100 MHz, DMSO-d_6) of compound D23 49
Figure S24-1. ^{1}H NMR spectrum (400 MHz, DMSO-d_6) of compound D24 50
Figure S24-2. ^{13}C NMR spectrum (100 MHz, DMSO-d_6) of compound D24 51
Figure S25-1. ^{1}H NMR spectrum (400 MHz, DMSO-d_6) of compound D25 52
Figure S25-2. ^{13}C NMR spectrum (100 MHz, DMSO-d_6) of compound D25 53
Figure S26-1. ^{1}H NMR spectrum (400 MHz, DMSO-d_6) of compound D26 54
Figure S26-2. ^{13}C NMR spectrum (100 MHz, DMSO-d_6) of compound D26 55
Figure S27-1. ^{1}H NMR spectrum (400 MHz, DMSO-d_6) of compound D27 56
Figure S27-2. ^{13}C NMR spectrum (100 MHz, DMSO-d_6) of compound D27 57
Figure S28-1. ^{1}H NMR spectrum (400 MHz, DMSO-d_6) of compound D28 58
Figure S28-2. ^{13}C NMR spectrum (100 MHz, DMSO-d_6) of compound D28 59
Figure S29-1. ^{1}H NMR spectrum (400 MHz, DMSO-d_6) of compound D29 60
Figure S29-2. ^{13}C NMR spectrum (100 MHz, DMSO-d_6) of compound D29 61
Figure S30-1. ^{1}H NMR spectrum (400 MHz, DMSO-d_6) of compound D30**Error! Bookmark not defined.**
Figure S30-2. ^{13}C NMR spectrum (100 MHz, DMSO-d_6) of compound D30**Error! Bookmark not defined.**
Figure S31-1. ^{1}H NMR spectrum (400 MHz, DMSO-d_6) of compound D31**Error! Bookmark not defined.**
Figure S31-2. ^{13}C NMR spectrum (100 MHz, DMSO-d_6) of compound D31**Error! Bookmark not defined.**
Figure S32-1. ^{1}H NMR spectrum (400 MHz, DMSO-d_6) of compound D32**Error! Bookmark not defined.**
Figure S32-2. ^{13}C NMR spectrum (100 MHz, DMSO-d_6) of compound D32**Error! Bookmark not defined.**

Figure S1-1. ^{1}H NMR spectrum (400MHz, DMSO-d_6) of compound D1

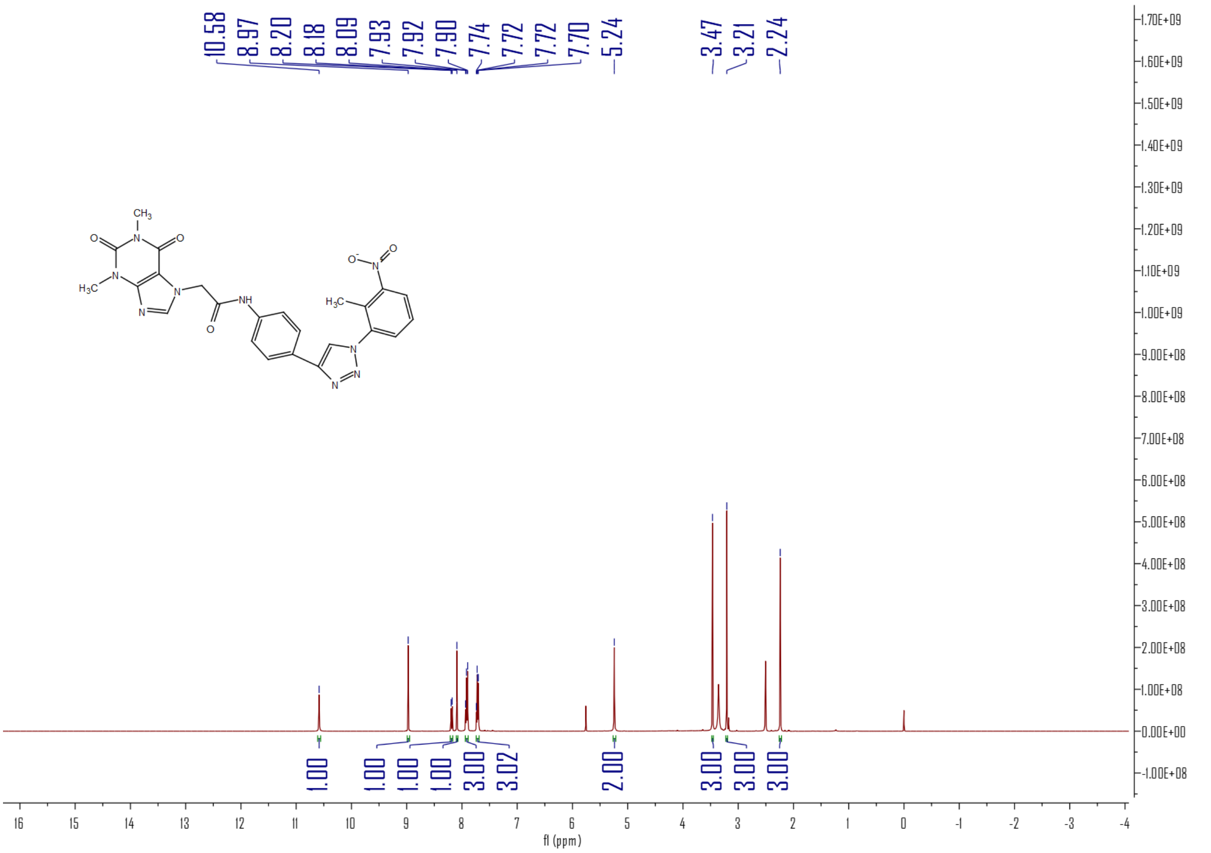

Figure S1-2. ^{13}C NMR spectrum (100MHz, DMSO-d_6) of compound D1

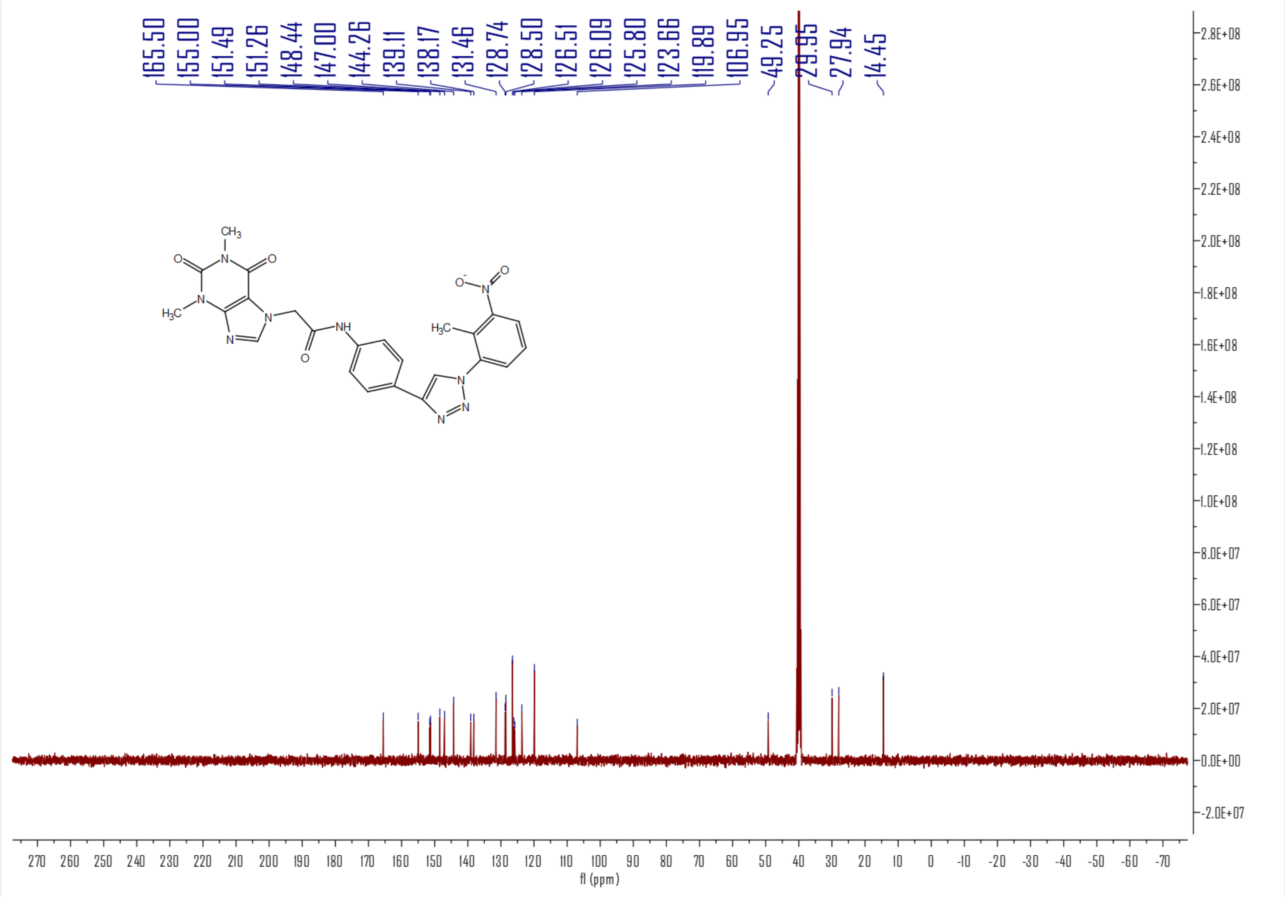

Figure S2-1. ^{1}H NMR spectrum (400MHz, DMSO-d_6) of compound D2

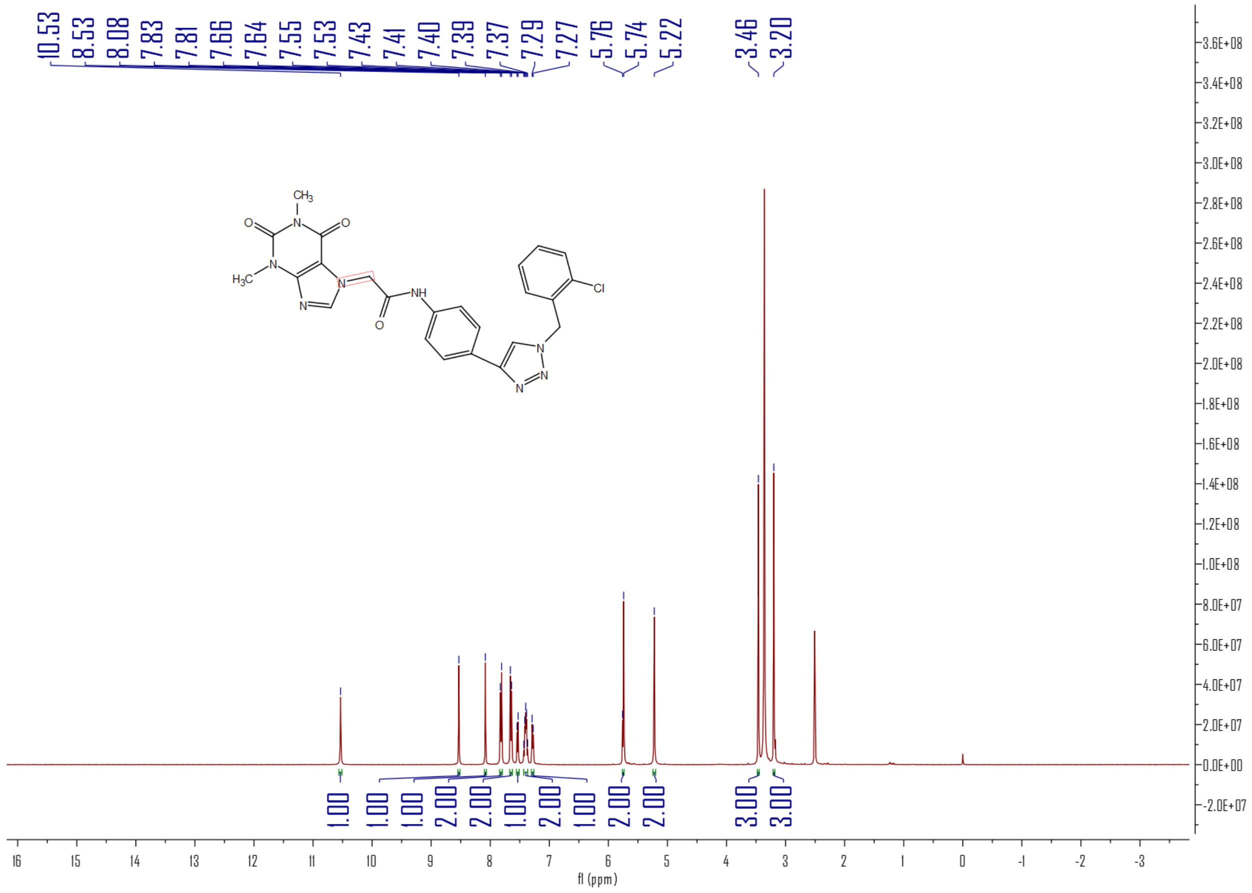

Figure S2-2. ^{13}C NMR spectrum (100MHz, DMSO-d$_6$) of compound D2

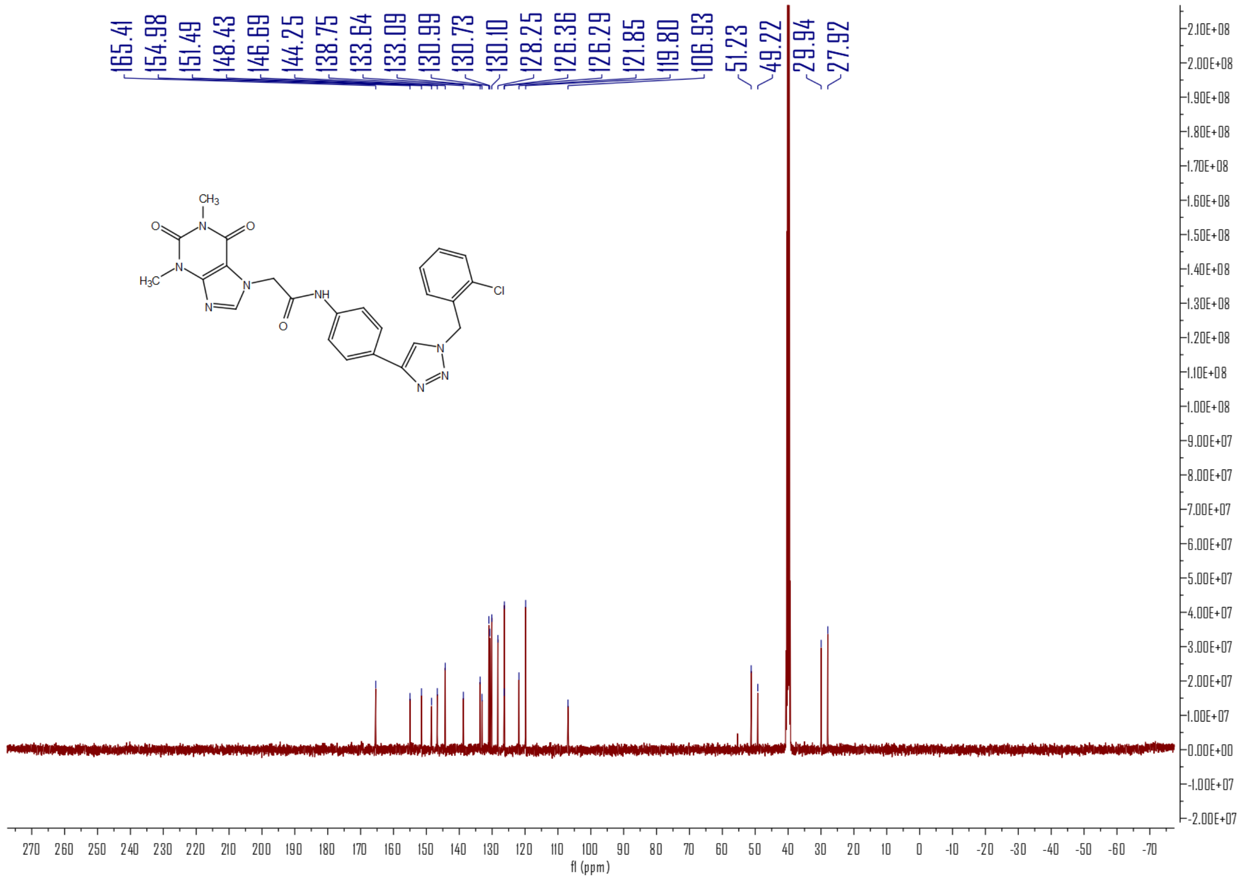

Figure S3-1. ^{1}H NMR spectrum (400MHz, DMSO-d$_6$) of compound D3

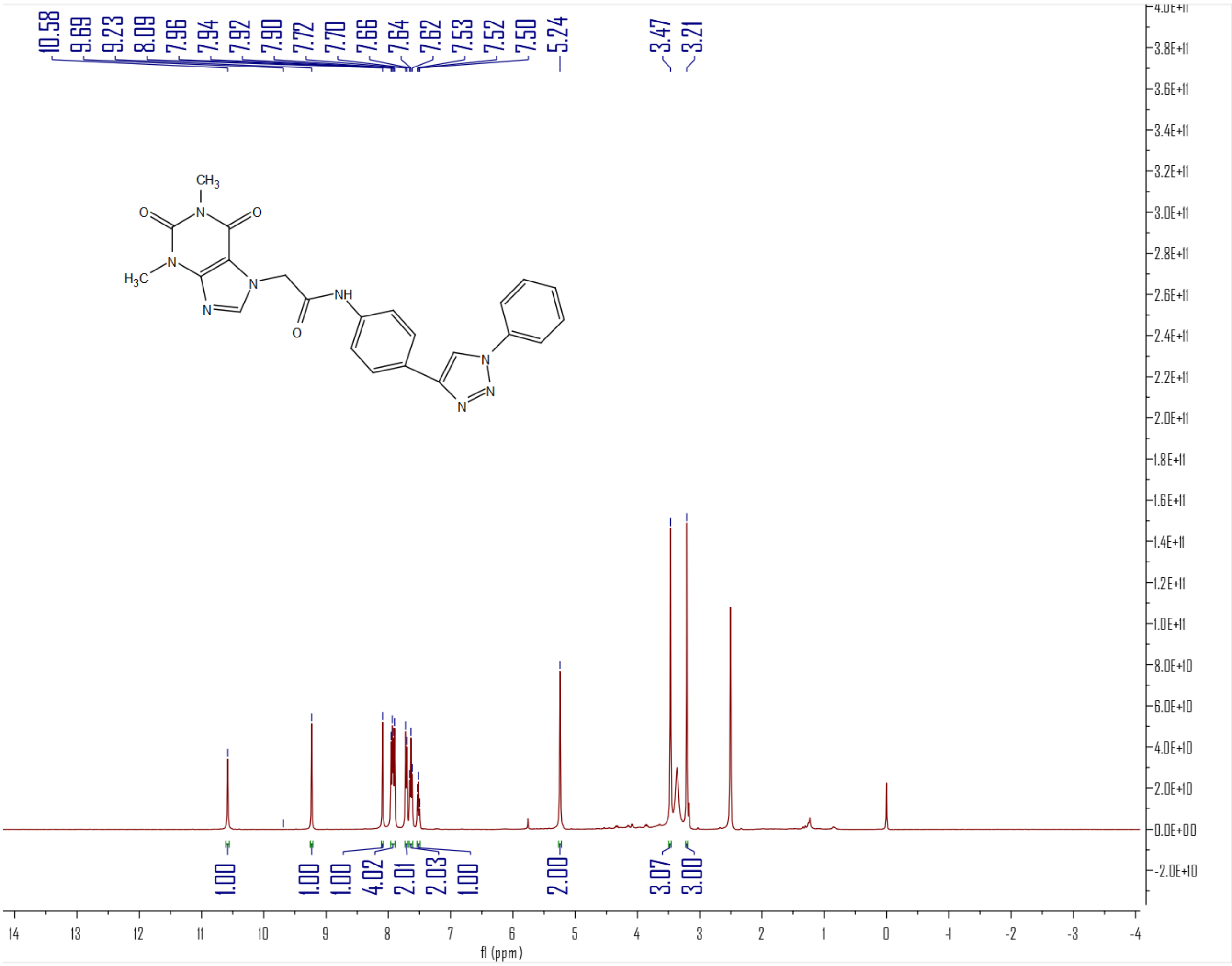

Figure S3-2. ^{13}C **NMR spectrum (100MHz, DMSO-** d_6 **) of compound D3**

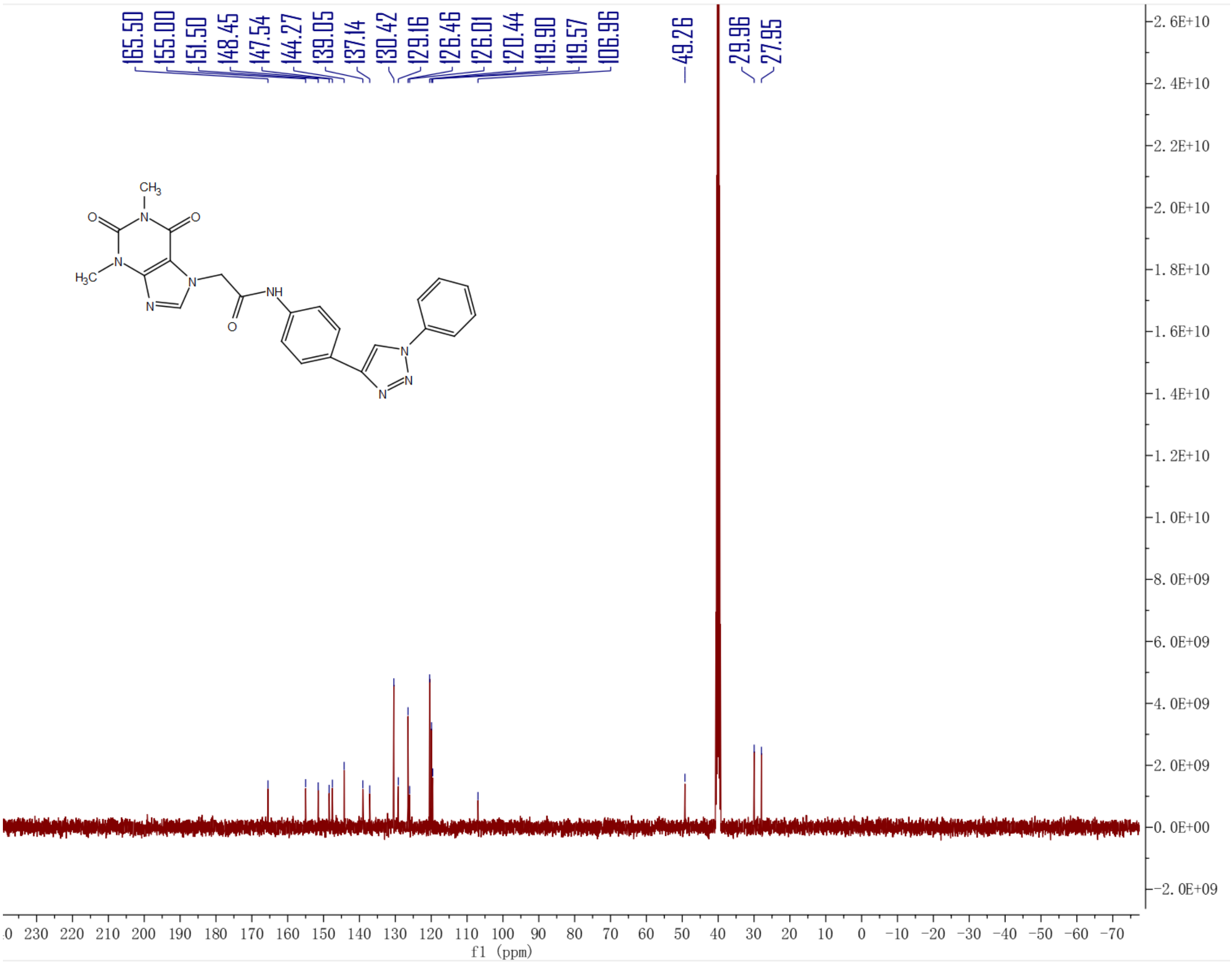

Figure S4-1. ^{1}H NMR spectrum (400MHz, DMSO-d$_6$) of compound D4

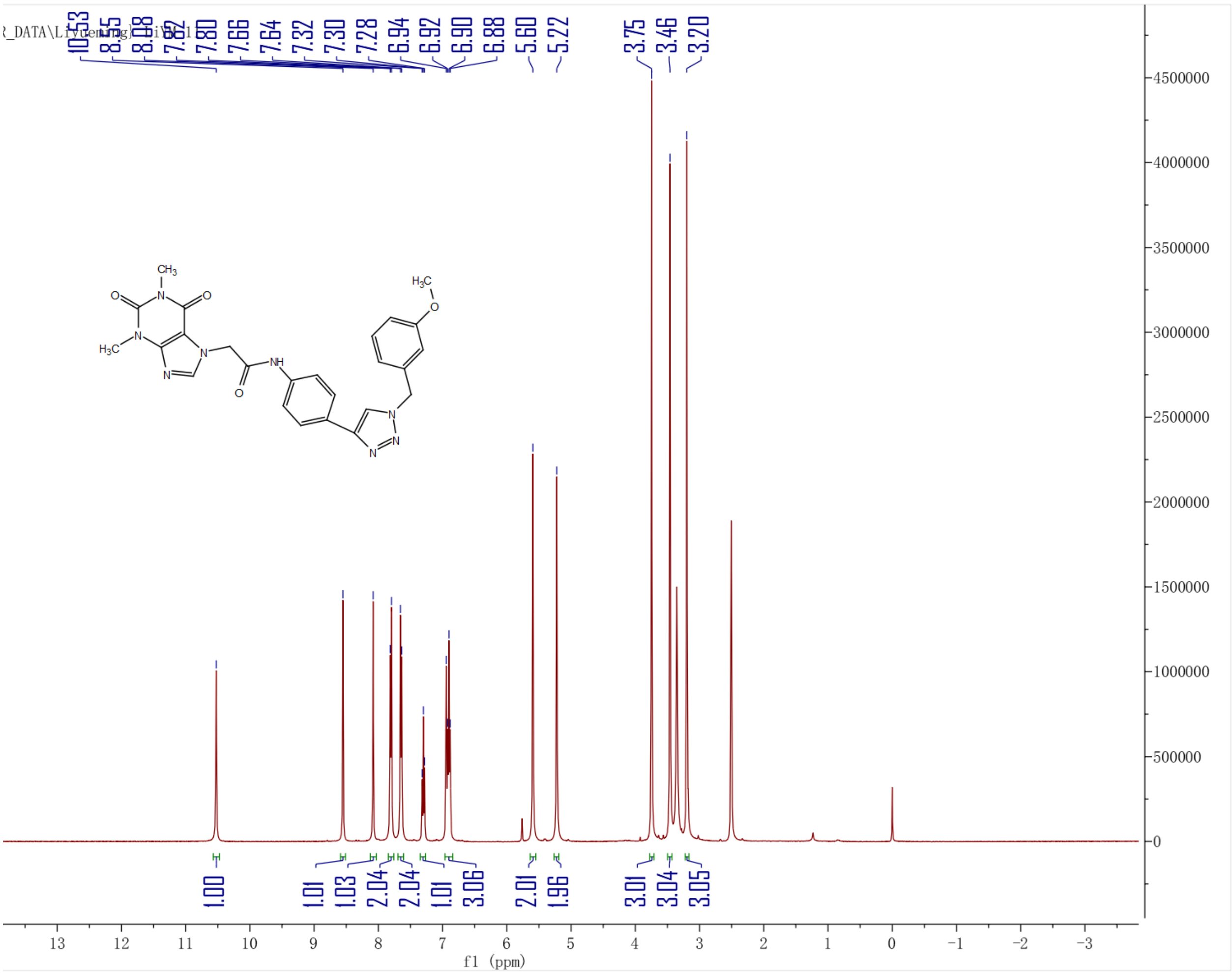

Figure S4-2. ^{13}C NMR spectrum (100MHz, DMSO-d_6) of compound D4

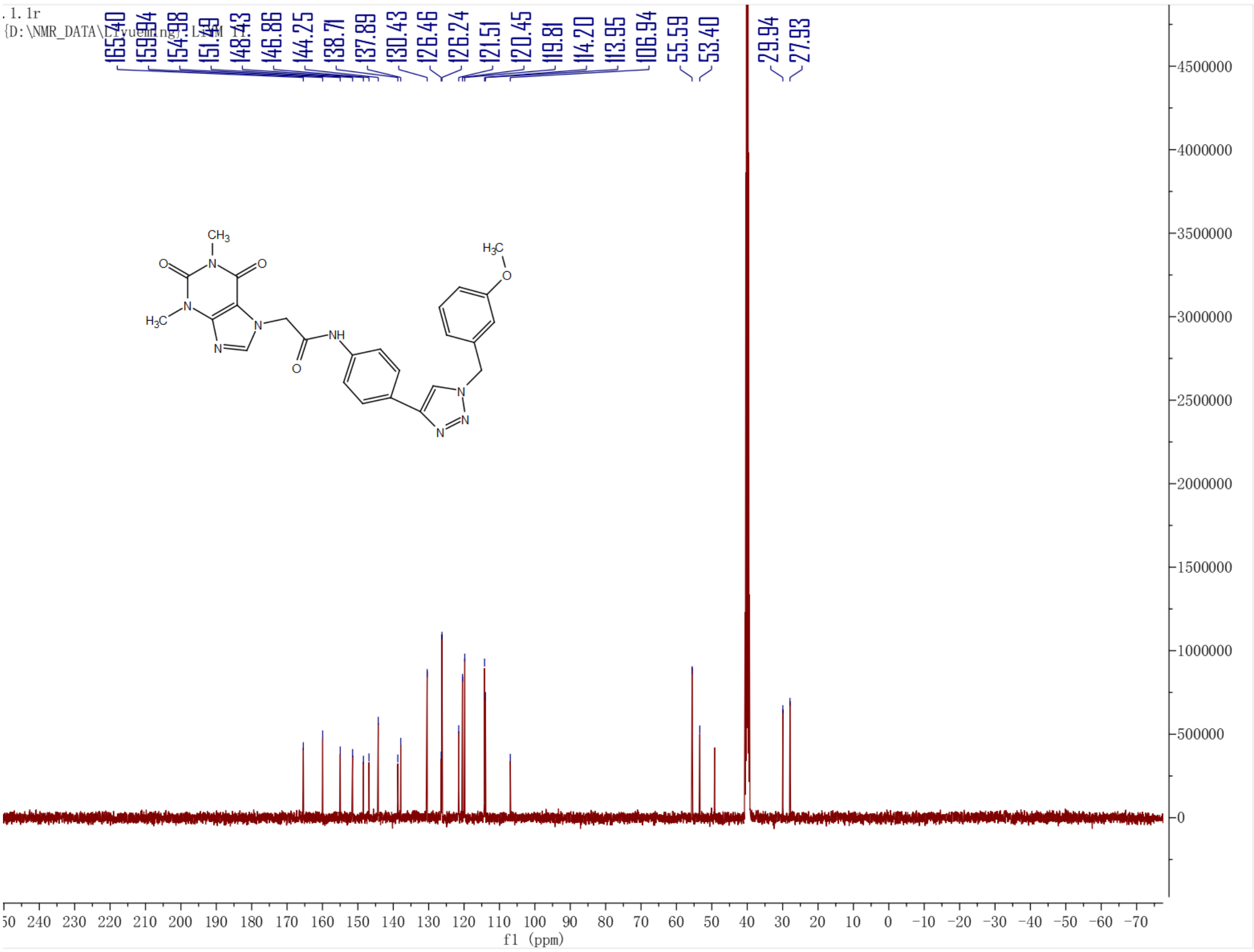

Figure S5-1. ^{1}H NMR spectrum (400MHz, DMSO-d_6) of compound D5

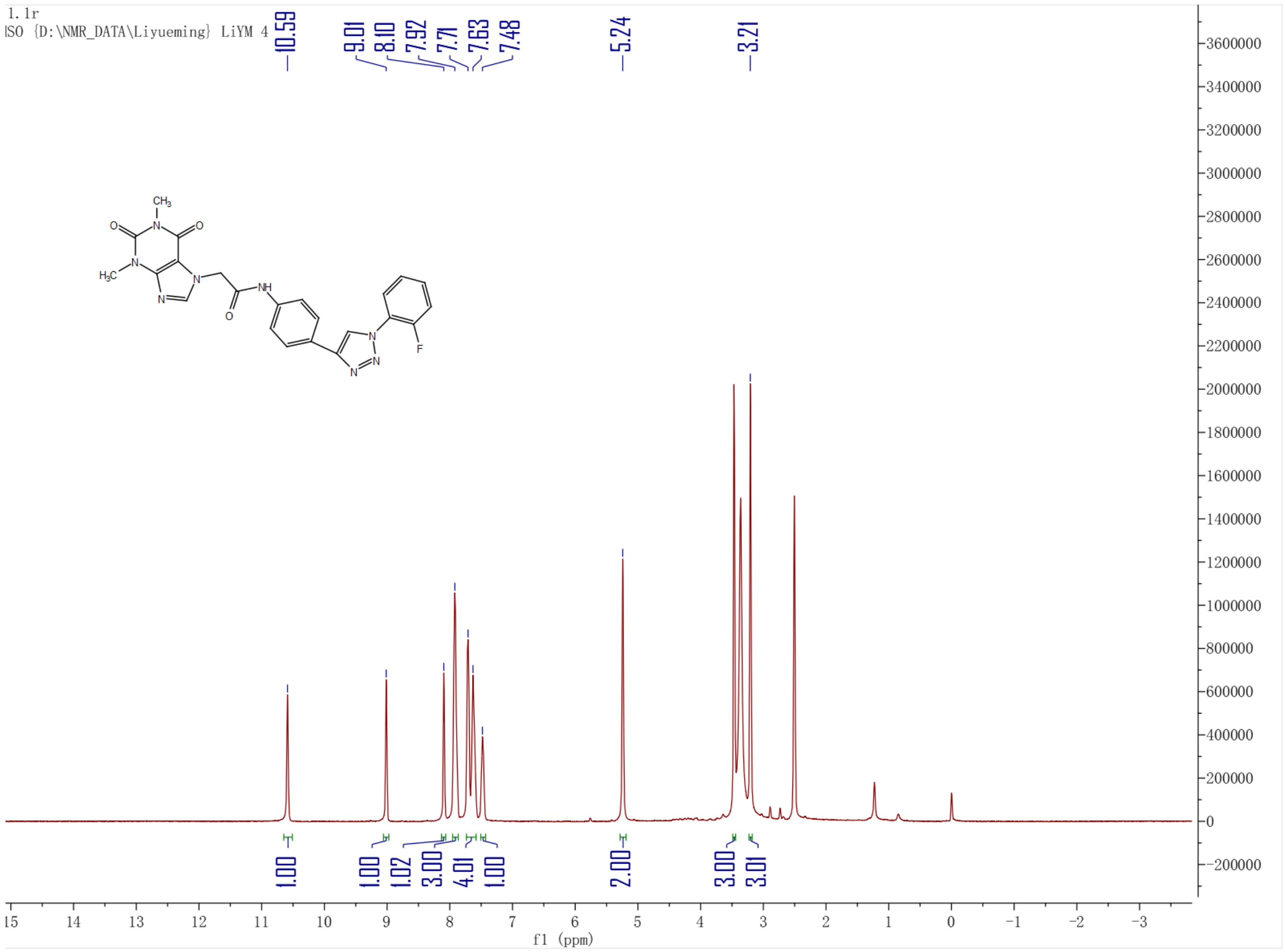

Figure S5-2. ^{13}C NMR spectrum (100MHz, DMSO-d_6) of compound D5

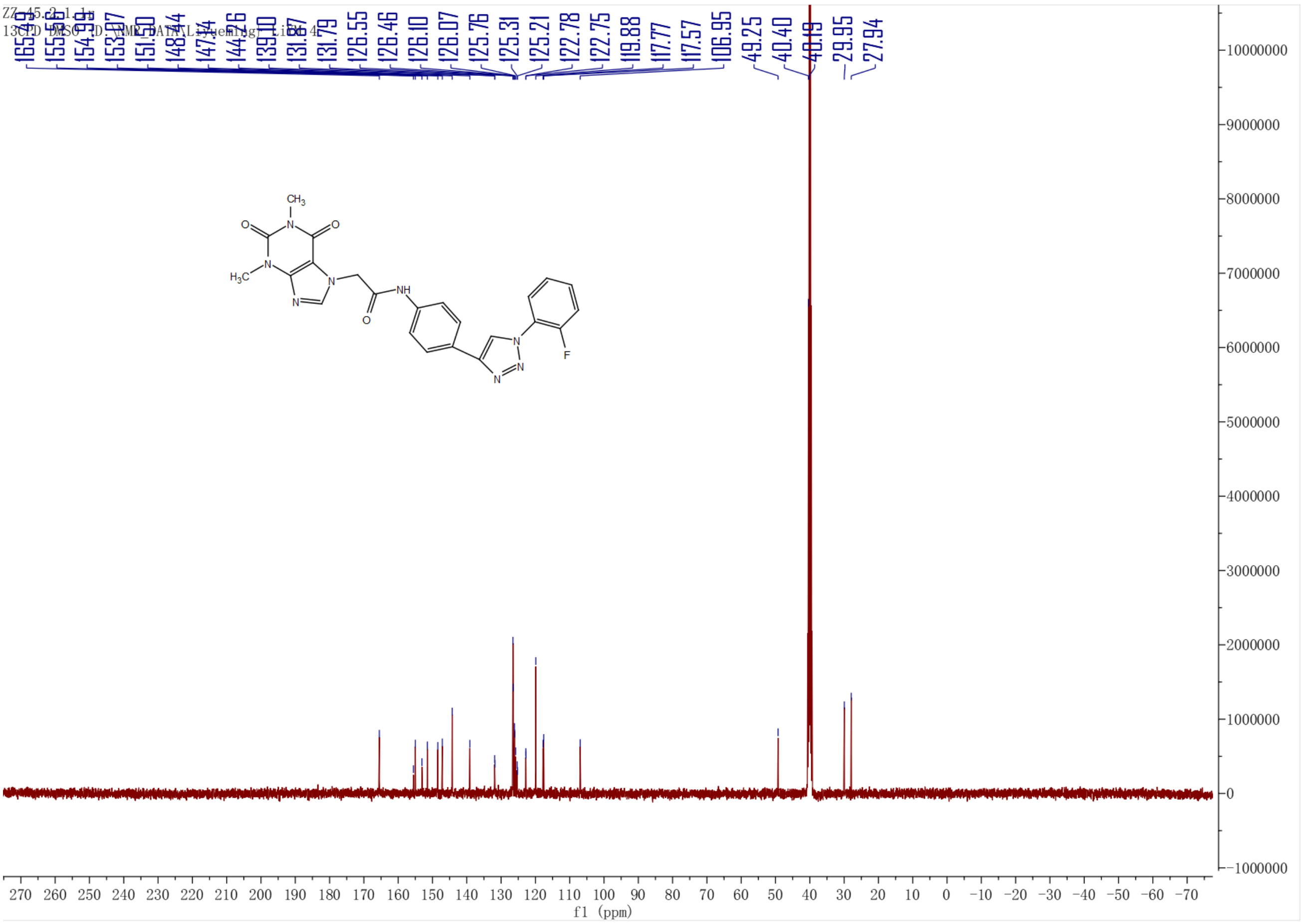

Figure S6-1. ^{1}H NMR spectrum (400MHz, DMSO-d_6) of compound D6

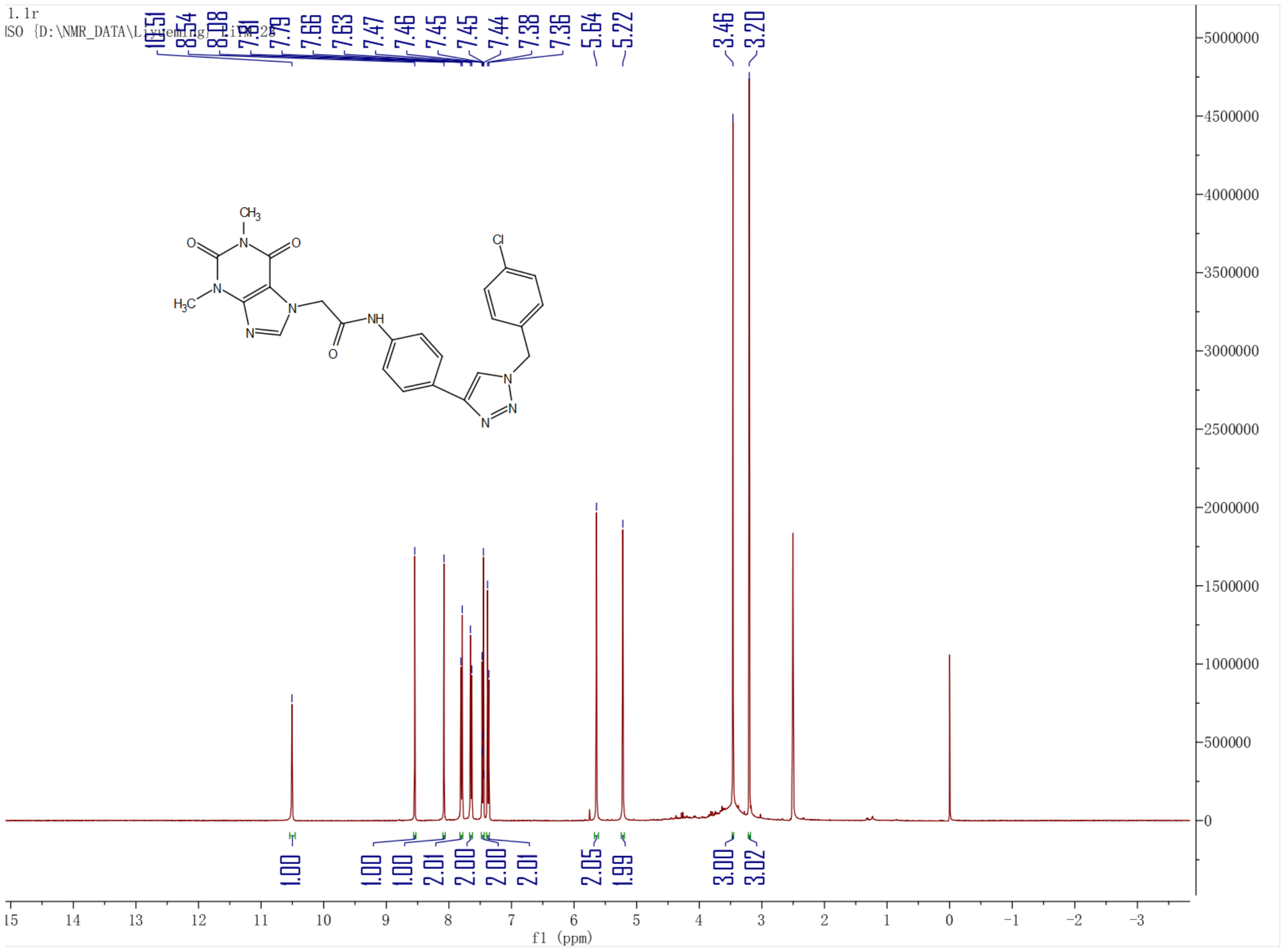

Figure S6-2. ^{13}C NMR spectrum (100MHz, DMSO-d_6) of compound D6

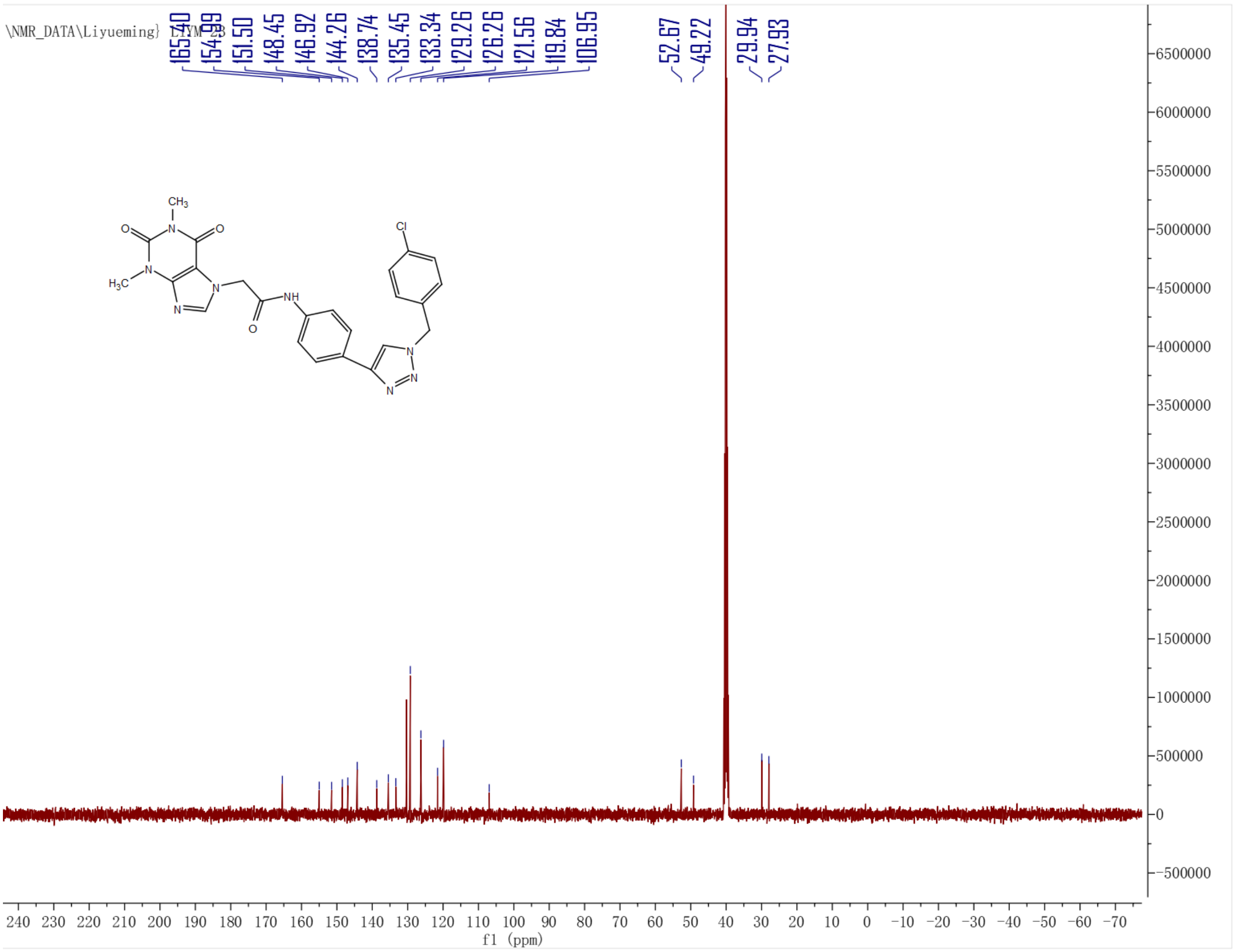

Figure S7-1. ^{1}H NMR spectrum (400MHz, DMSO-d$_6$) of compound D7

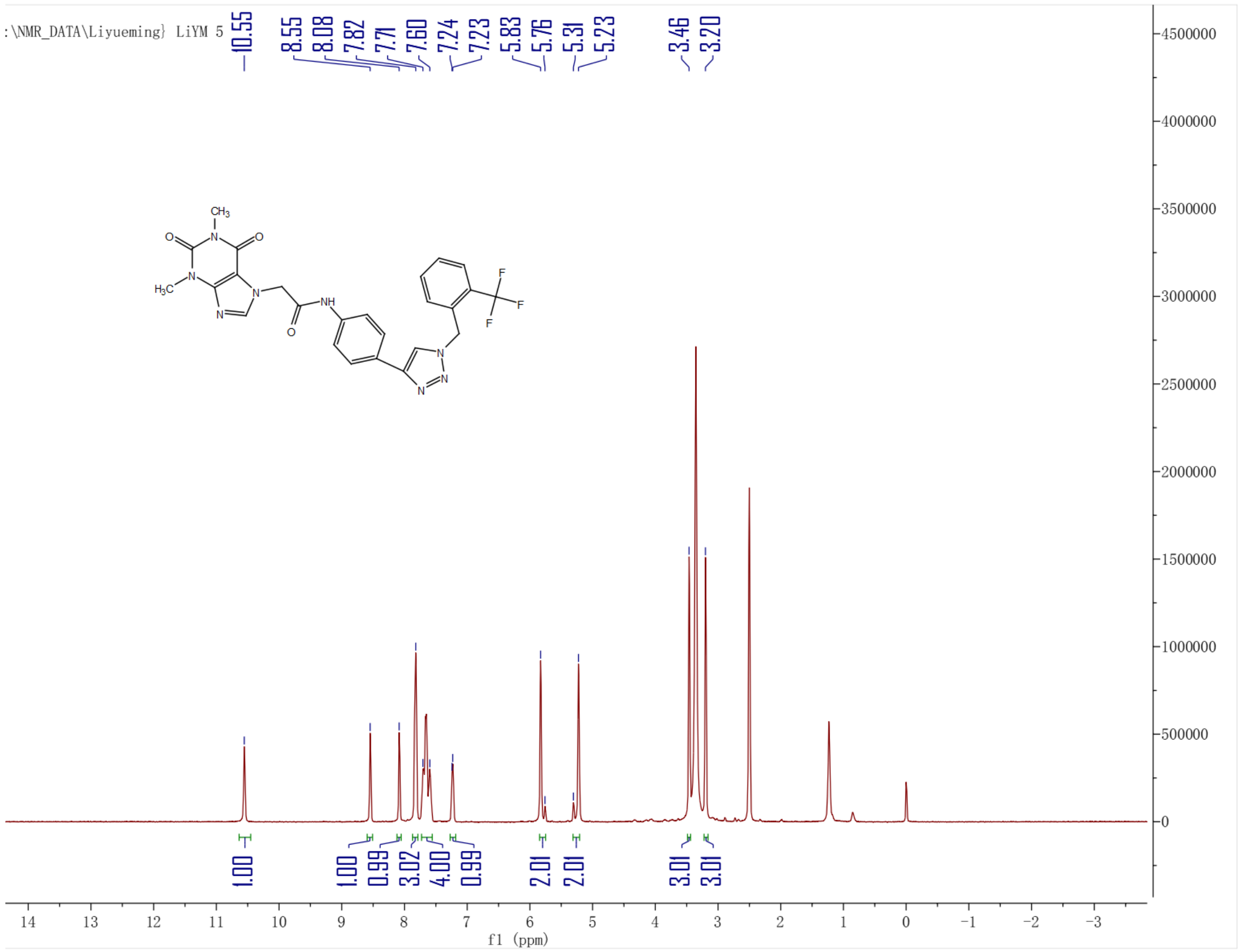

Figure S7-2. ^{13}C NMR spectrum (100MHz, DMSO-d_6) of compound D7

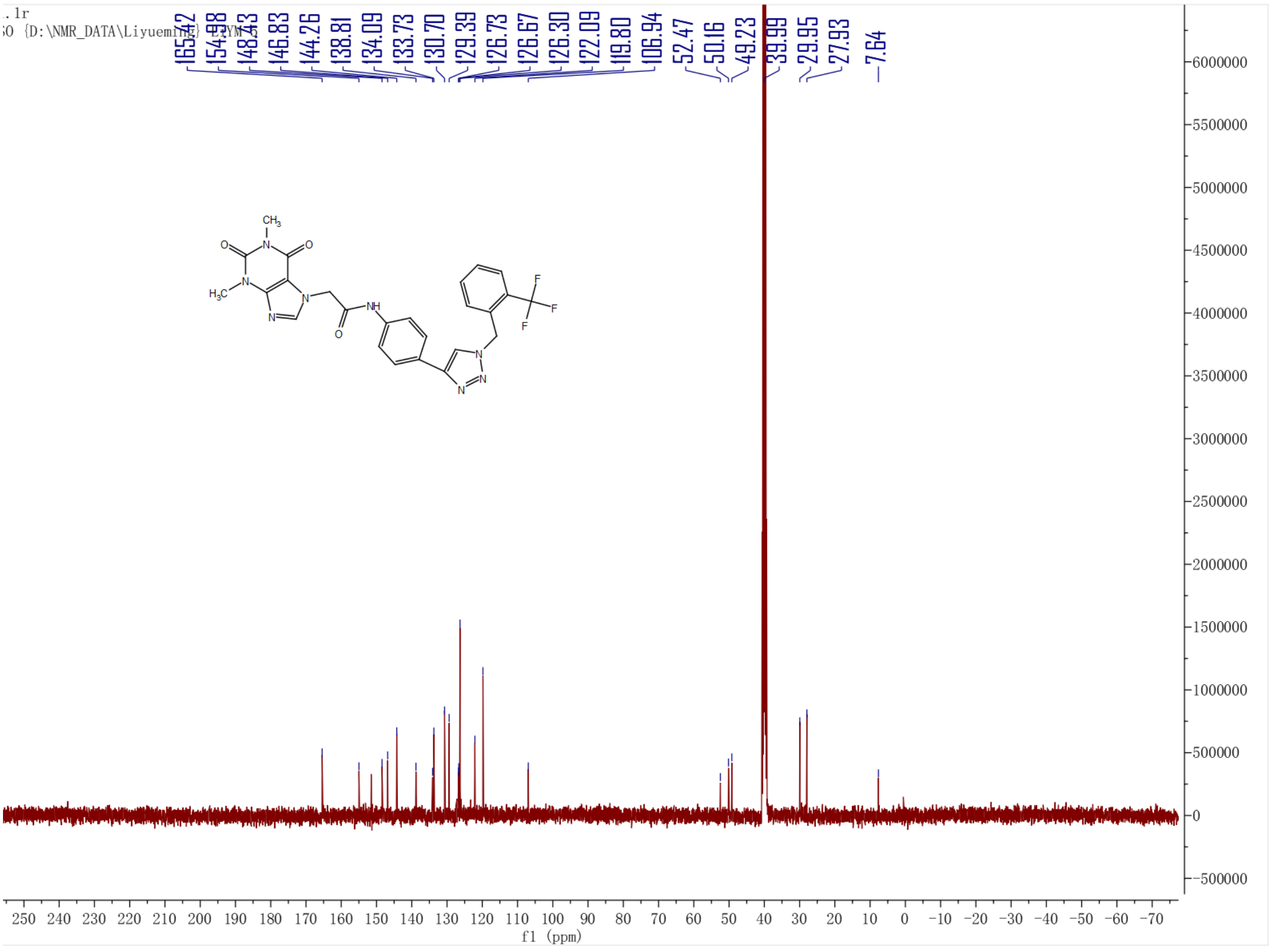

Figure S8-1. ^{1}H NMR spectrum (400MHz, DMSO-d$_6$) of compound D8

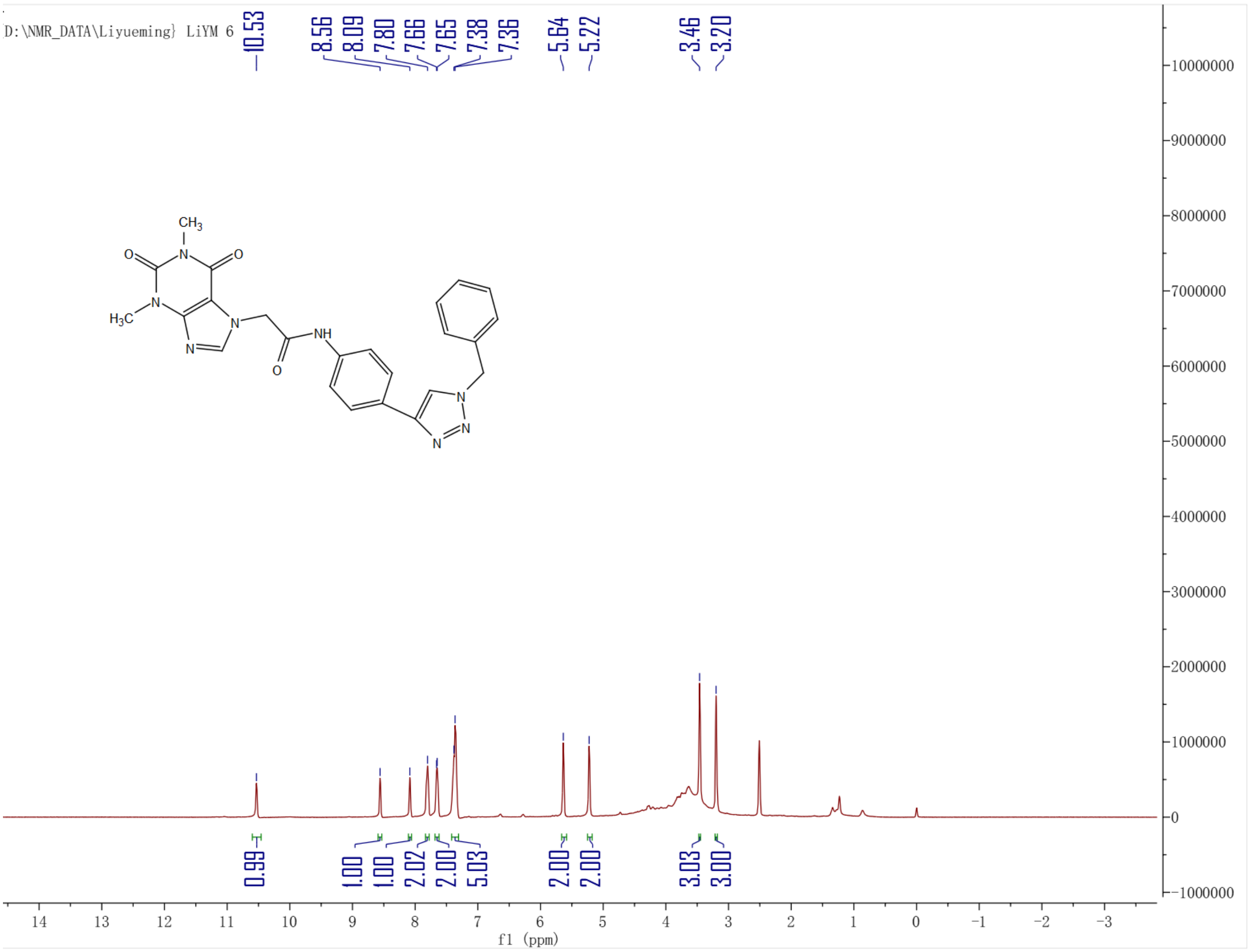

Figure S8-2. ^{13}C NMR spectrum (100MHz, DMSO-d_6) of compound D8

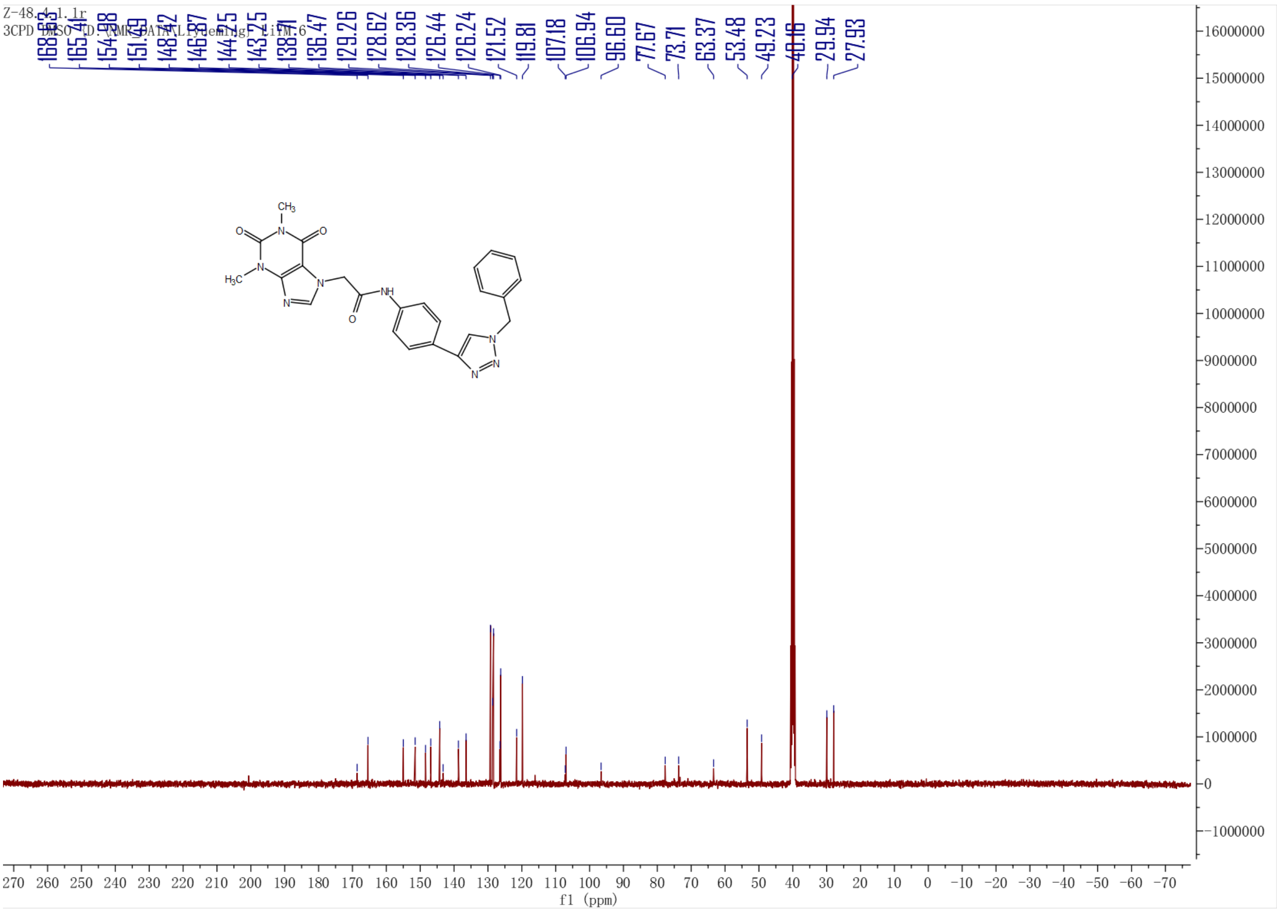

Figure S9-1. ^{1}H NMR spectrum (400MHz, DMSO-d_6) of compound D9

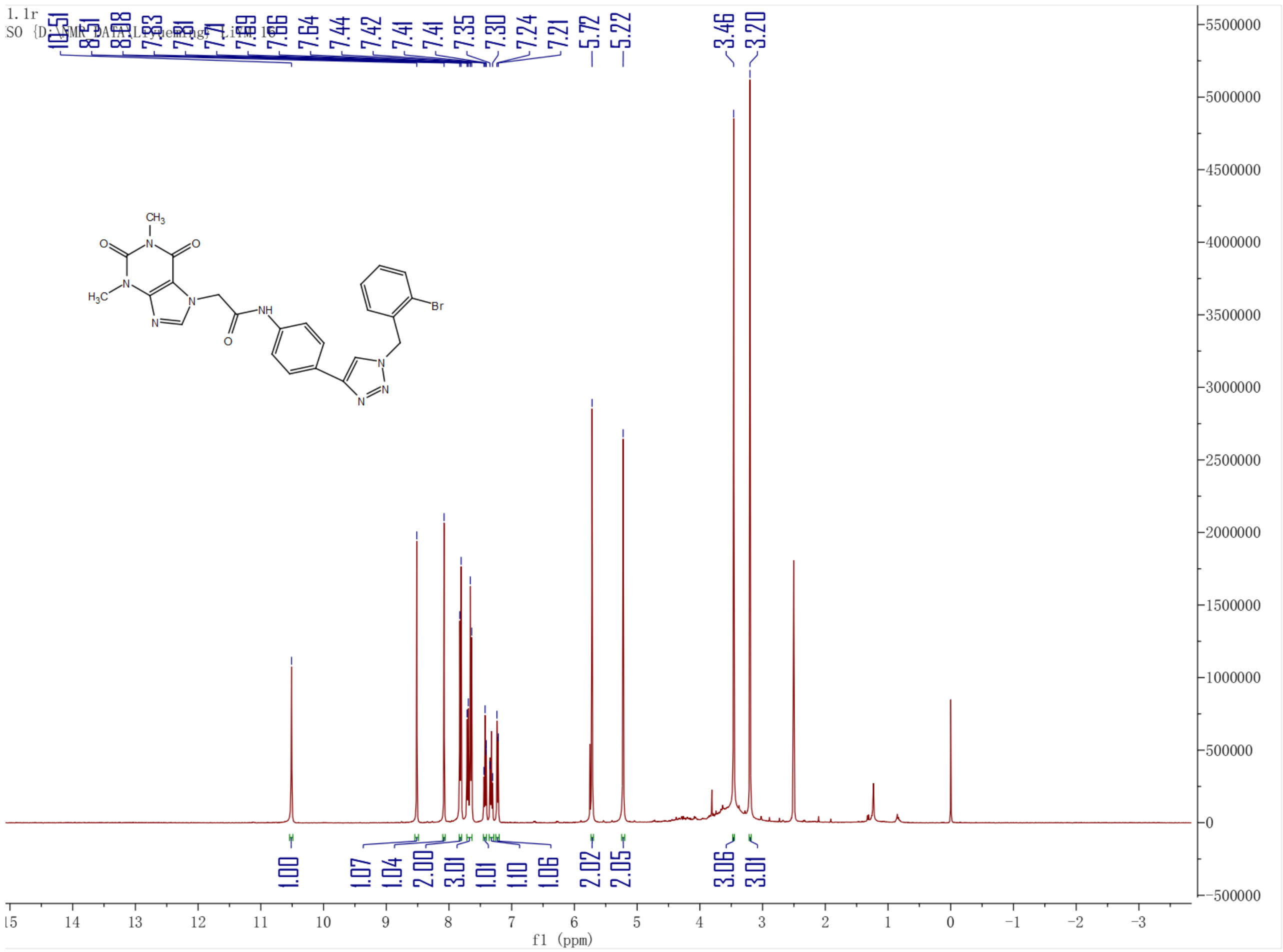

Figure S9-2. ^{13}C NMR spectrum (100MHz, DMSO-d$_6$) of compound D9

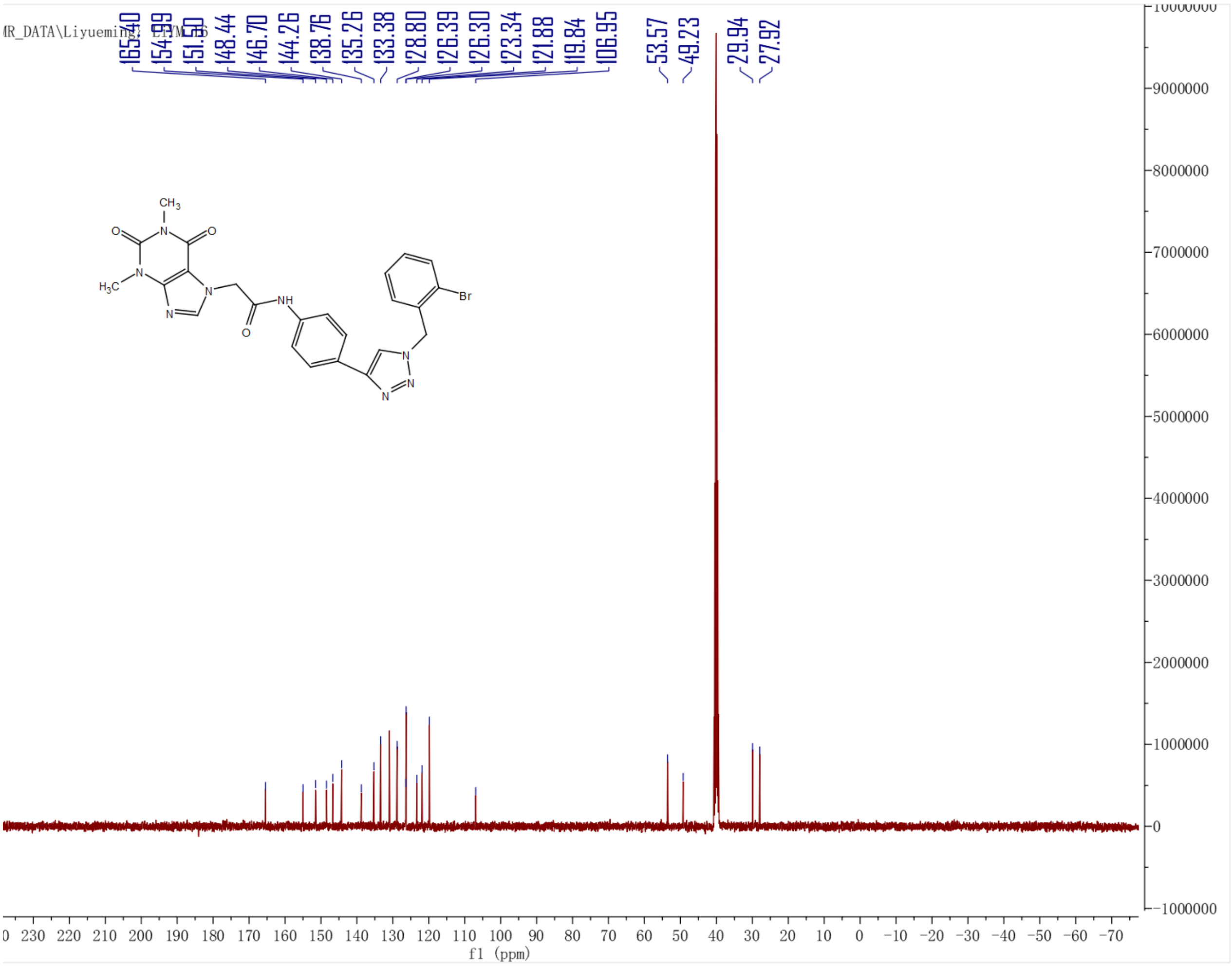

Figure S10-1. ^{1}H NMR spectrum (400MHz, DMSO-d_6) of compound D10

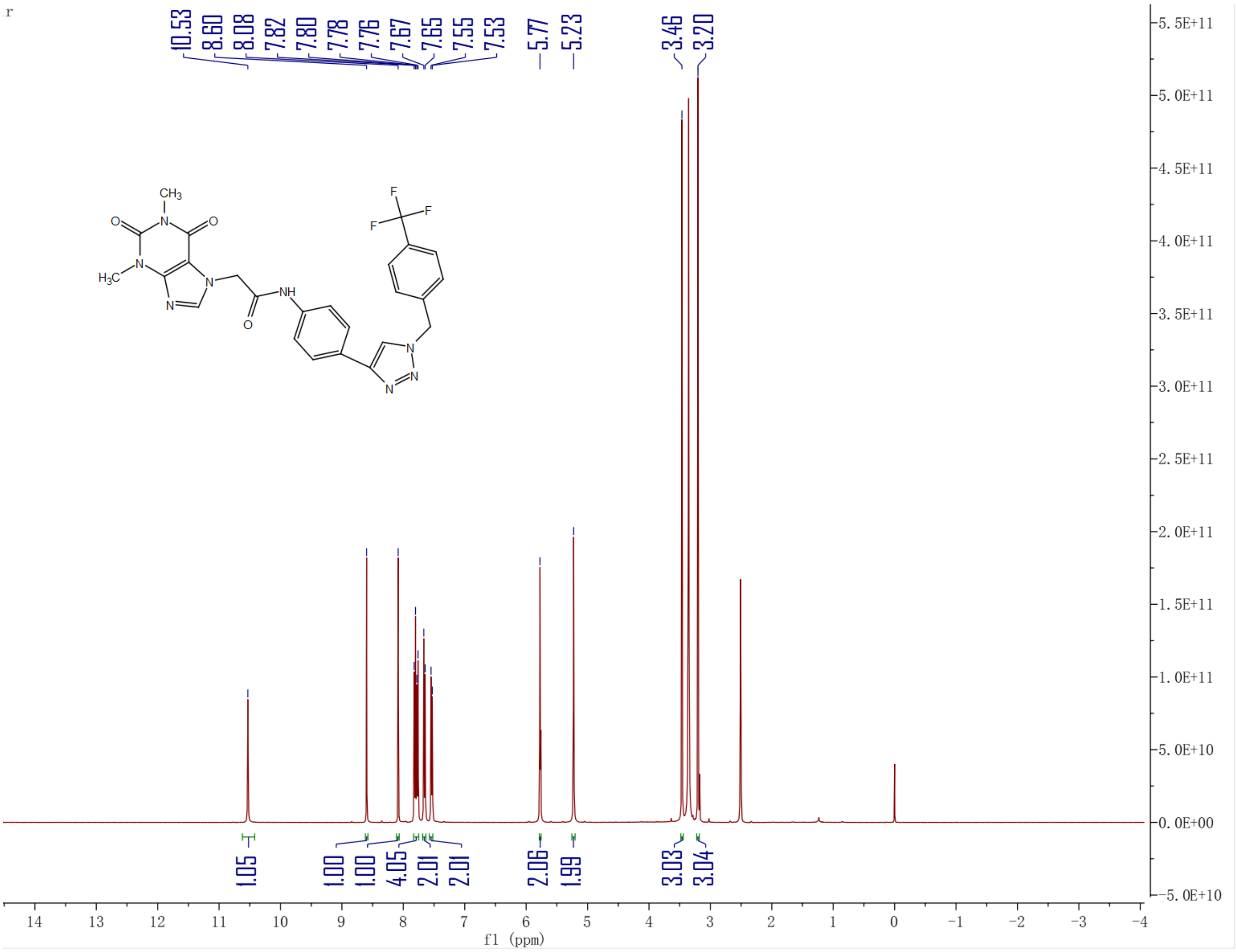

Figure S10-2. ^{13}C NMR spectrum (100MHz, DMSO-d$_6$) of compound D10

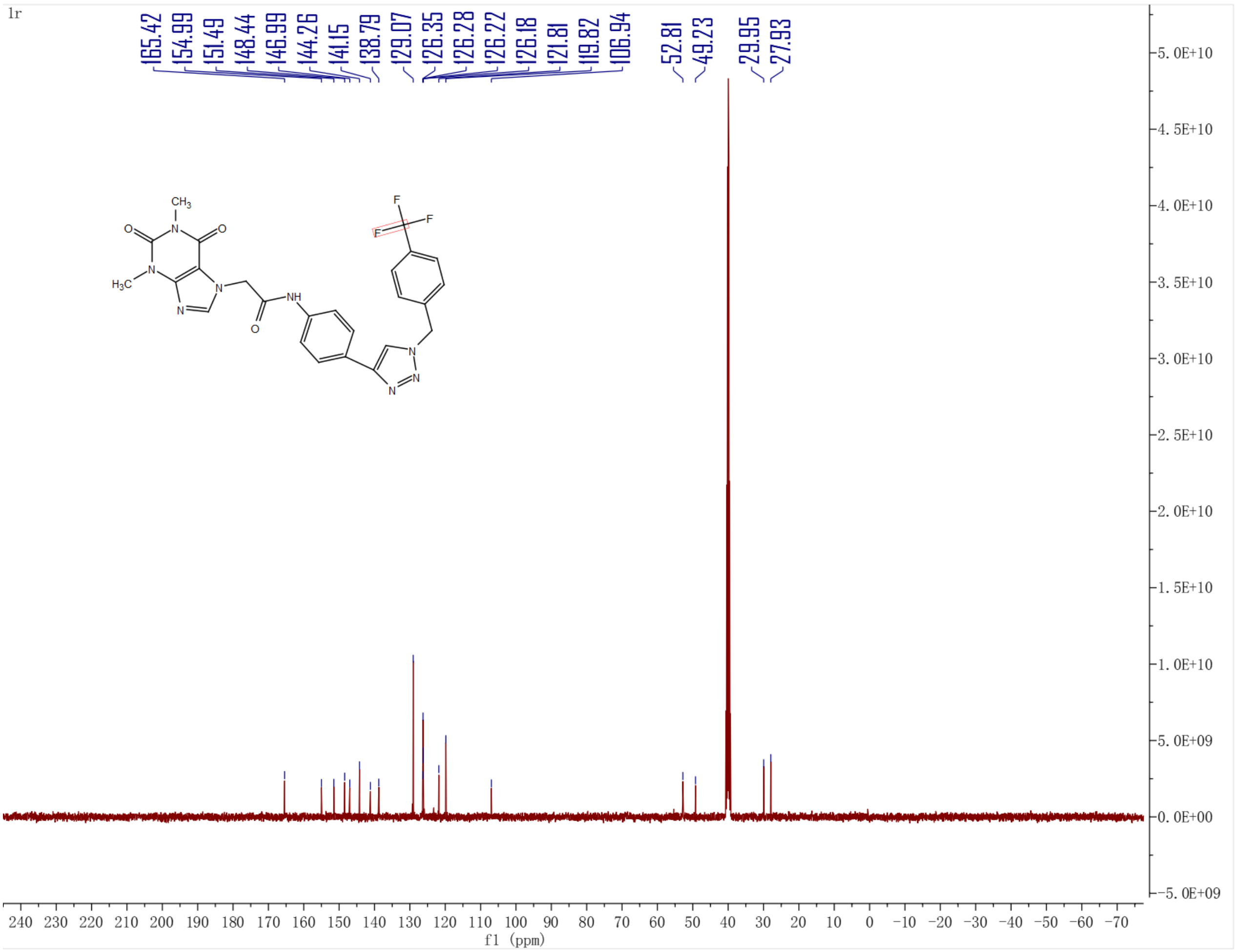

Figure S11-1. ^{1}H NMR spectrum (400MHz, DMSO-d$_6$) of compound D11

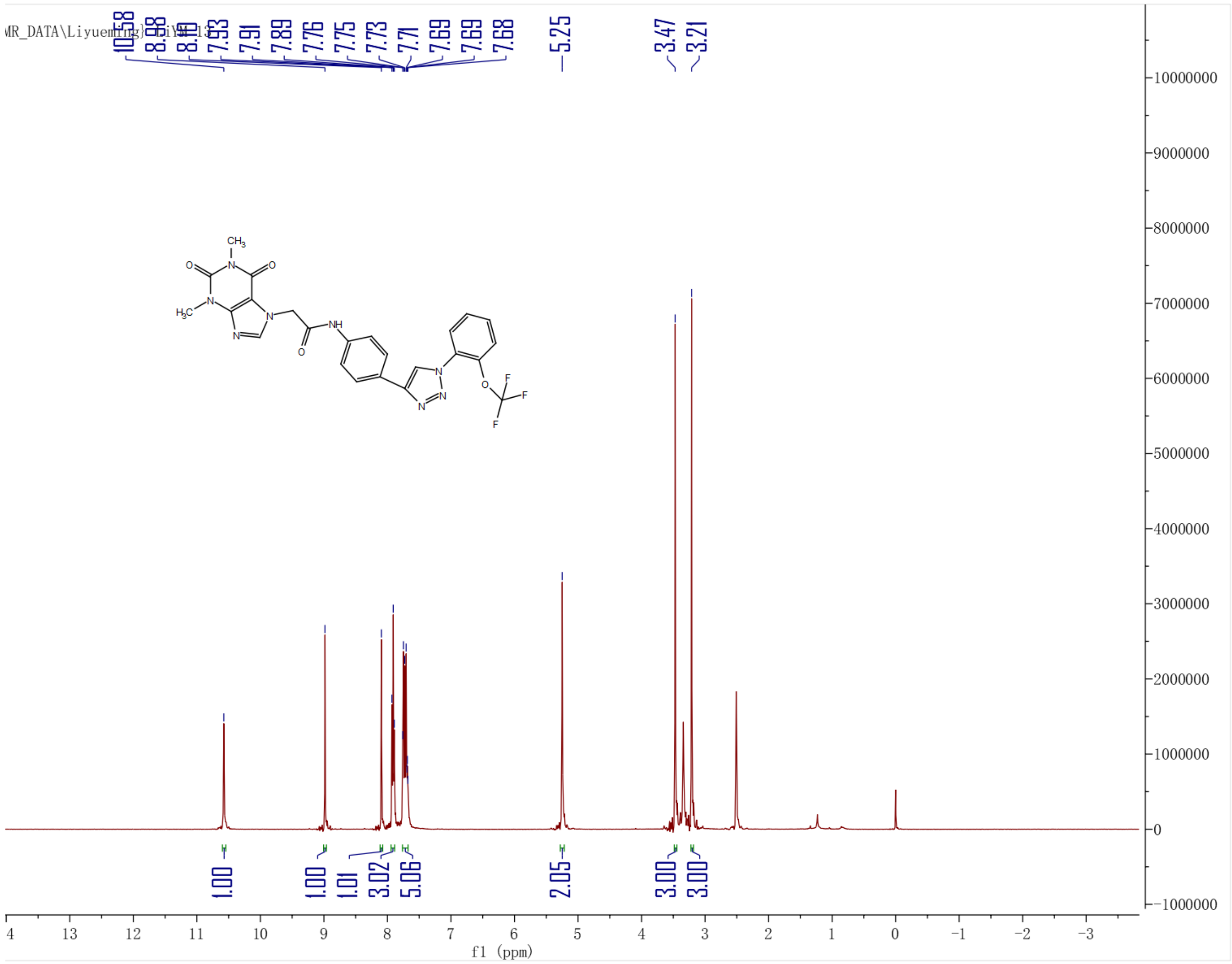

Figure S11-2. ^{13}C NMR spectrum (100MHz, DMSO-d_6) of compound D11

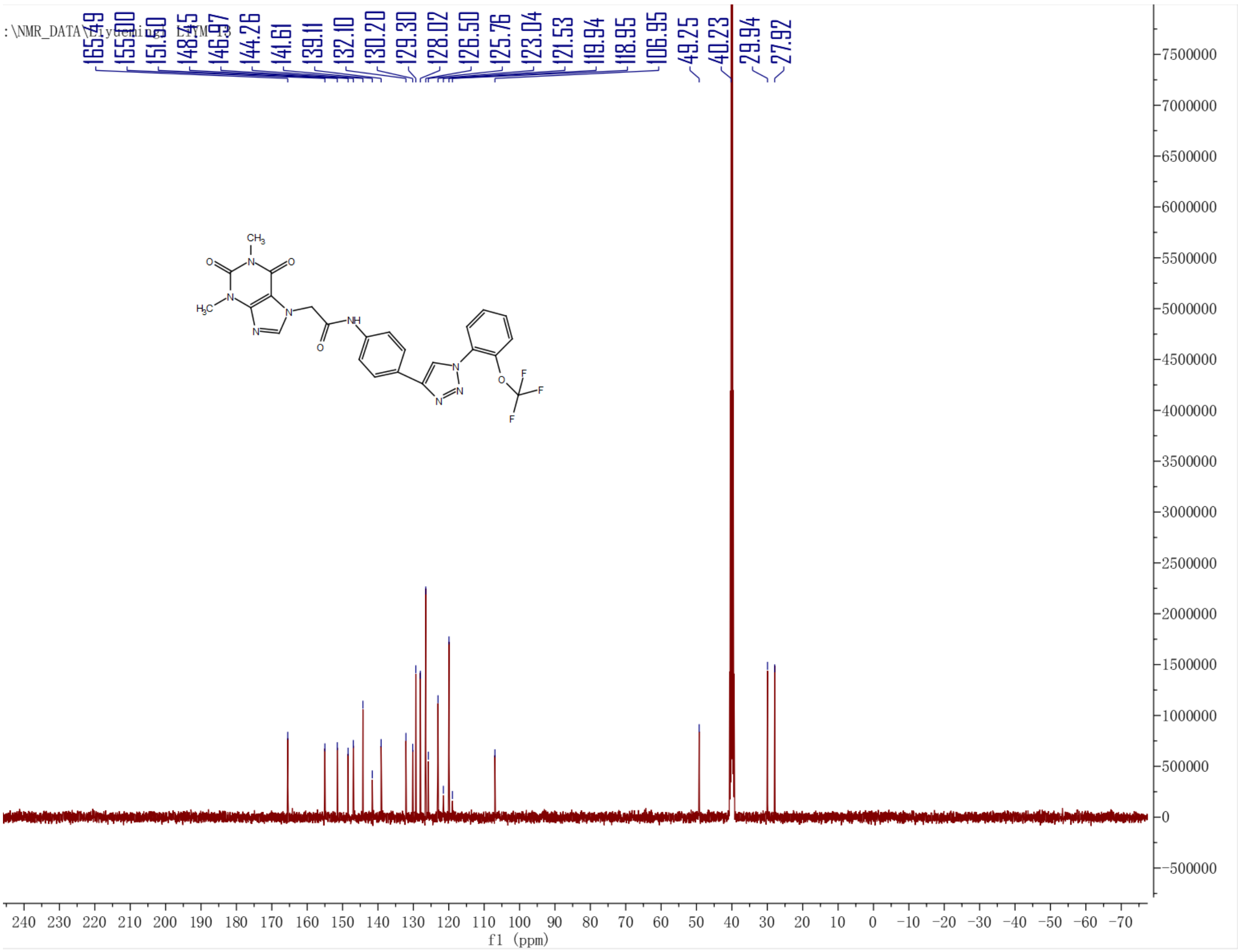

Figure S12-1. ^{1}H NMR spectrum (400MHz, DMSO-d$_6$) of compound D12

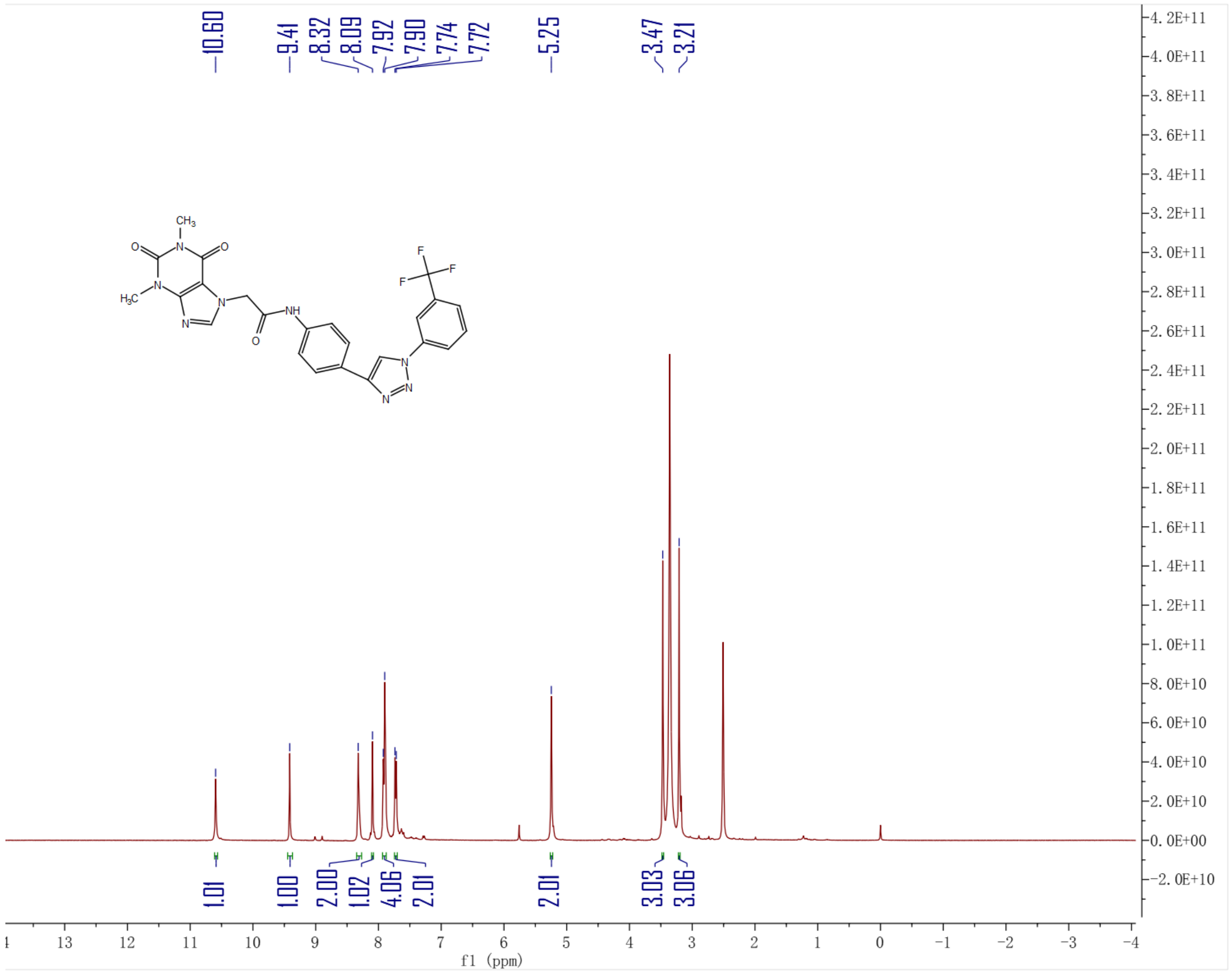

Figure S12-2. ^{12}C NMR spectrum (100MHz, DMSO-d$_6$) of compound D12

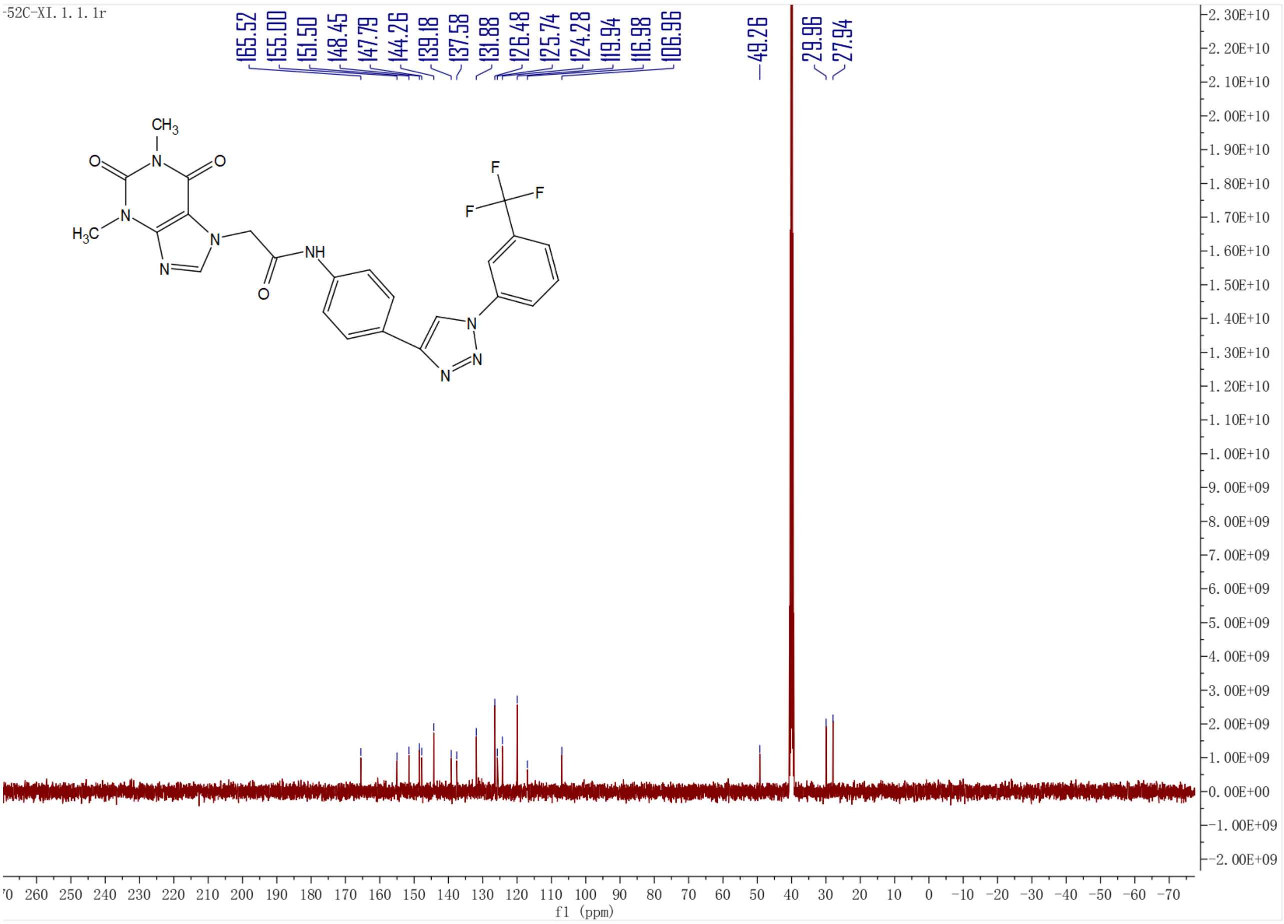

Figure S13-1. ^{1}H NMR spectrum (400MHz, DMSO-d$_6$) of compound D13

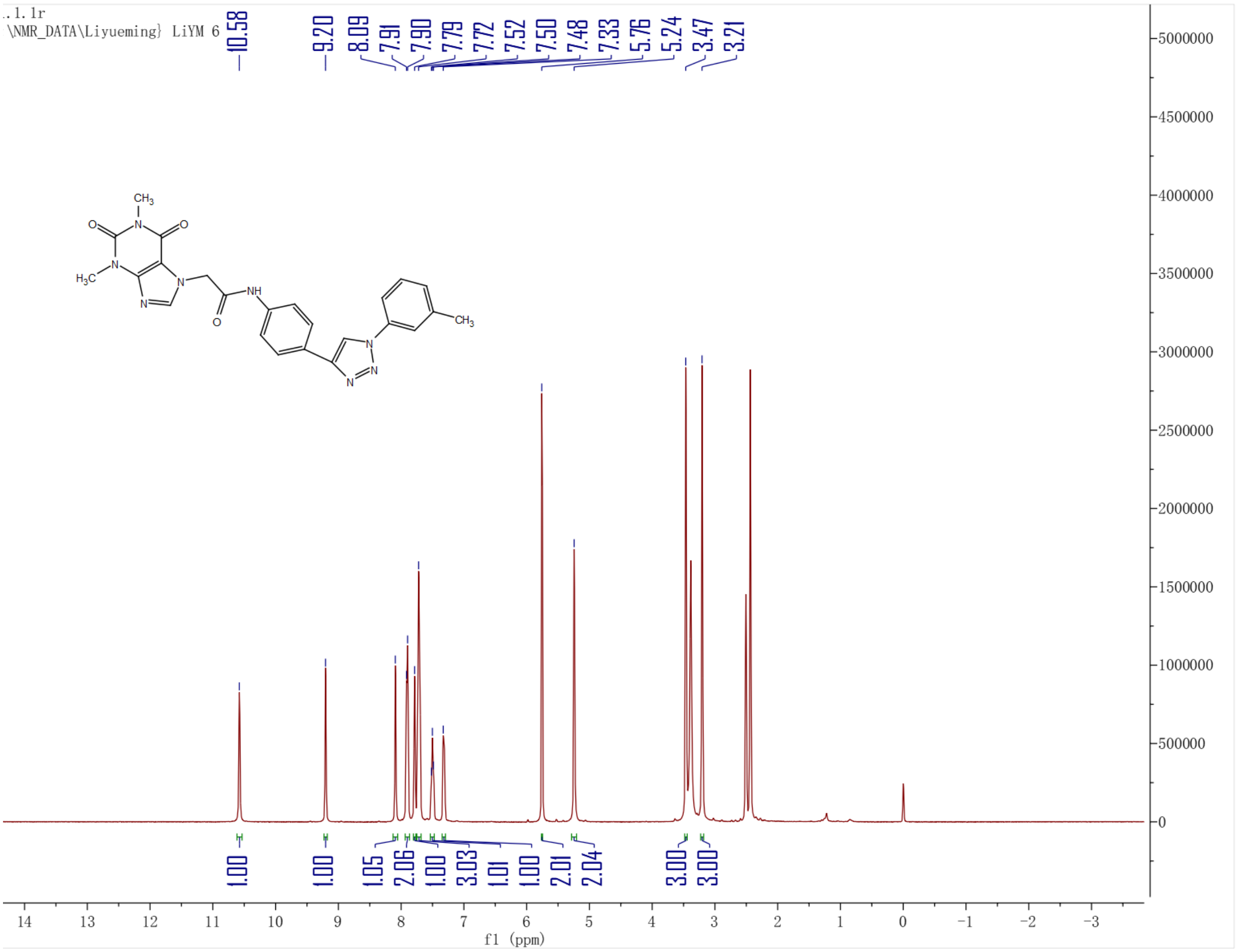

Figure S13-2. ^{13}C NMR spectrum (100MHz, DMSO-d$_6$) of compound D13

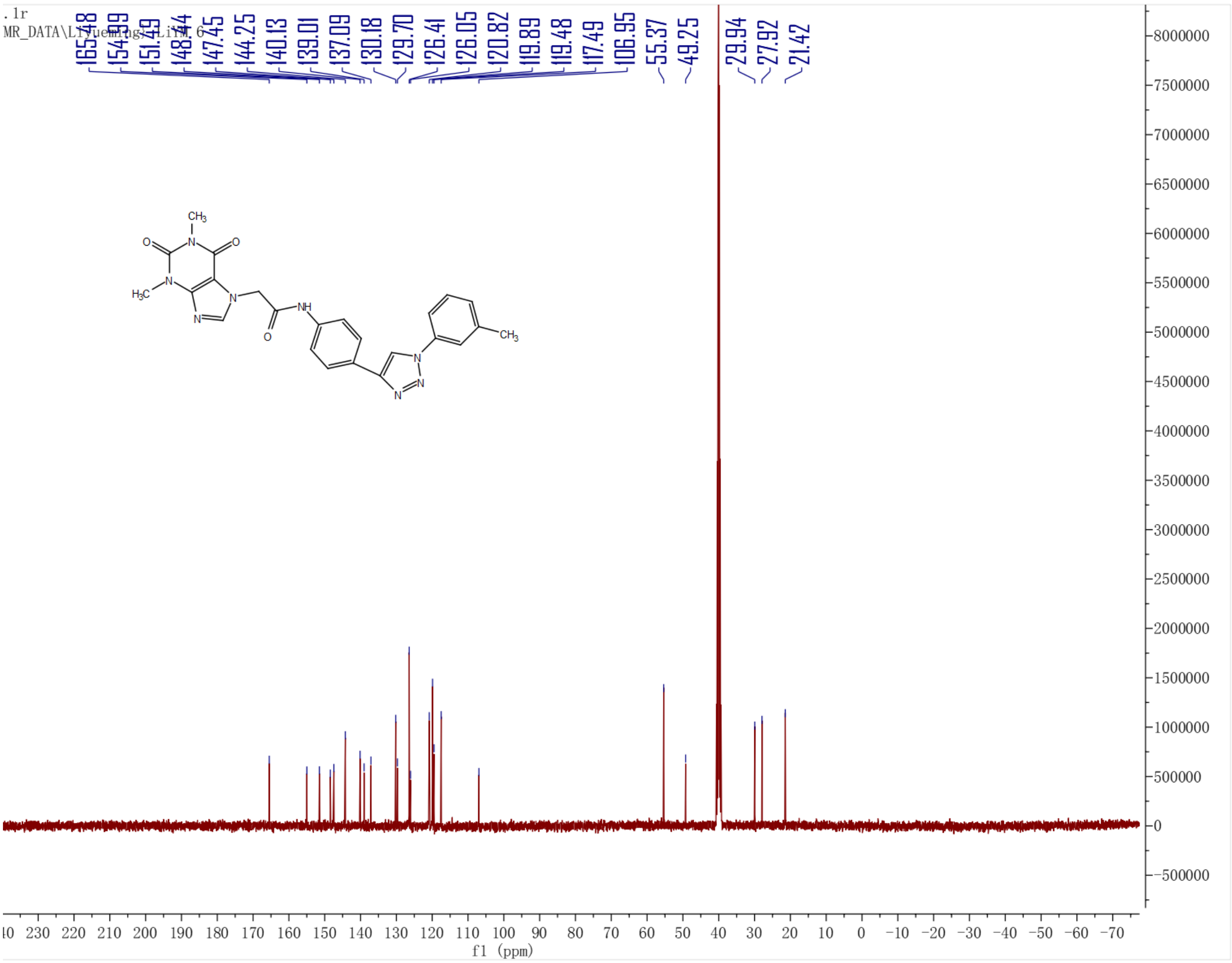

Figure S14-1. ^{1}H NMR spectrum (400MHz, DMSO-d$_6$) of compound D14

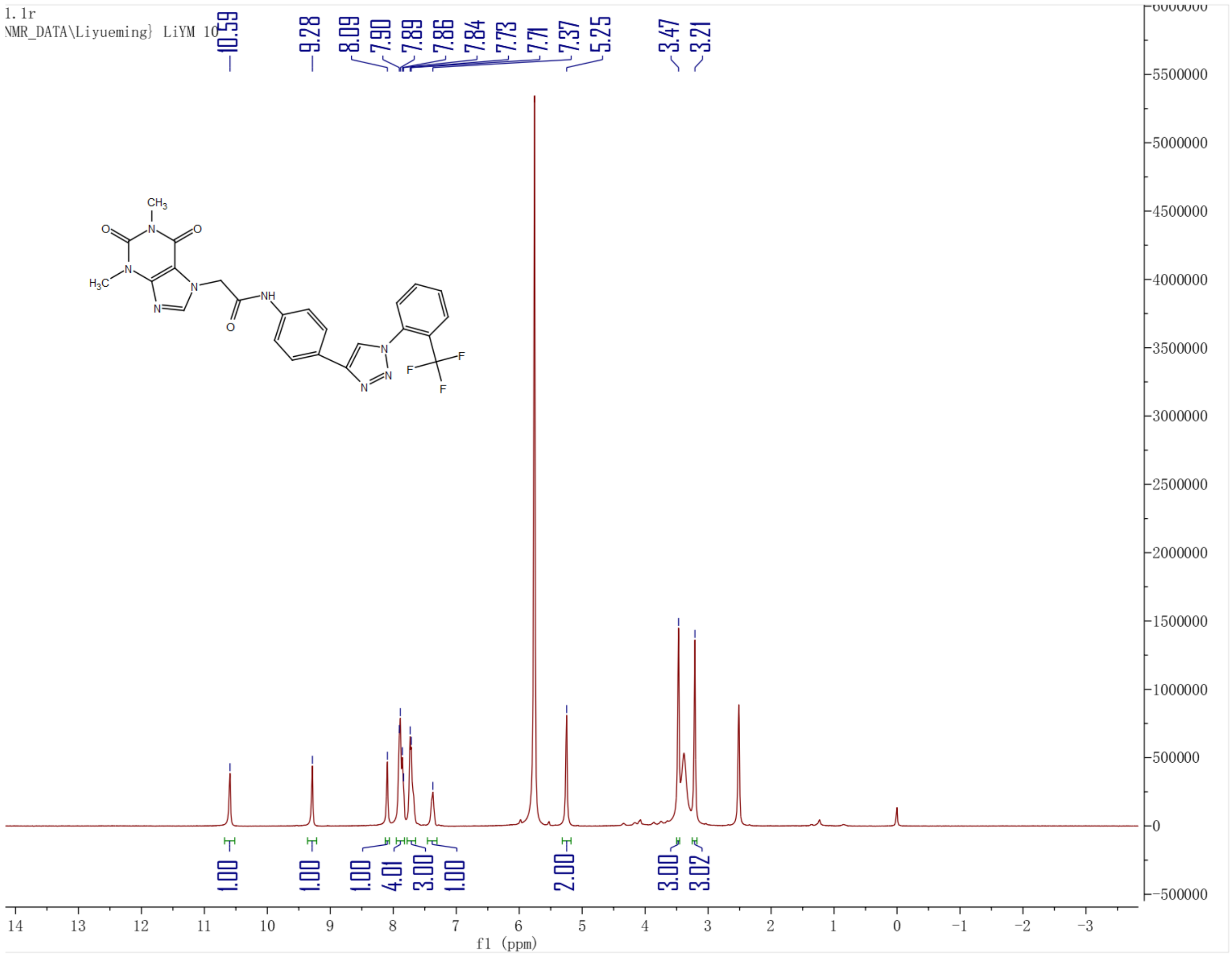

Figure S14-2. ^{13}C NMR spectrum (100MHz, DMSO-d$_6$) of compound D14

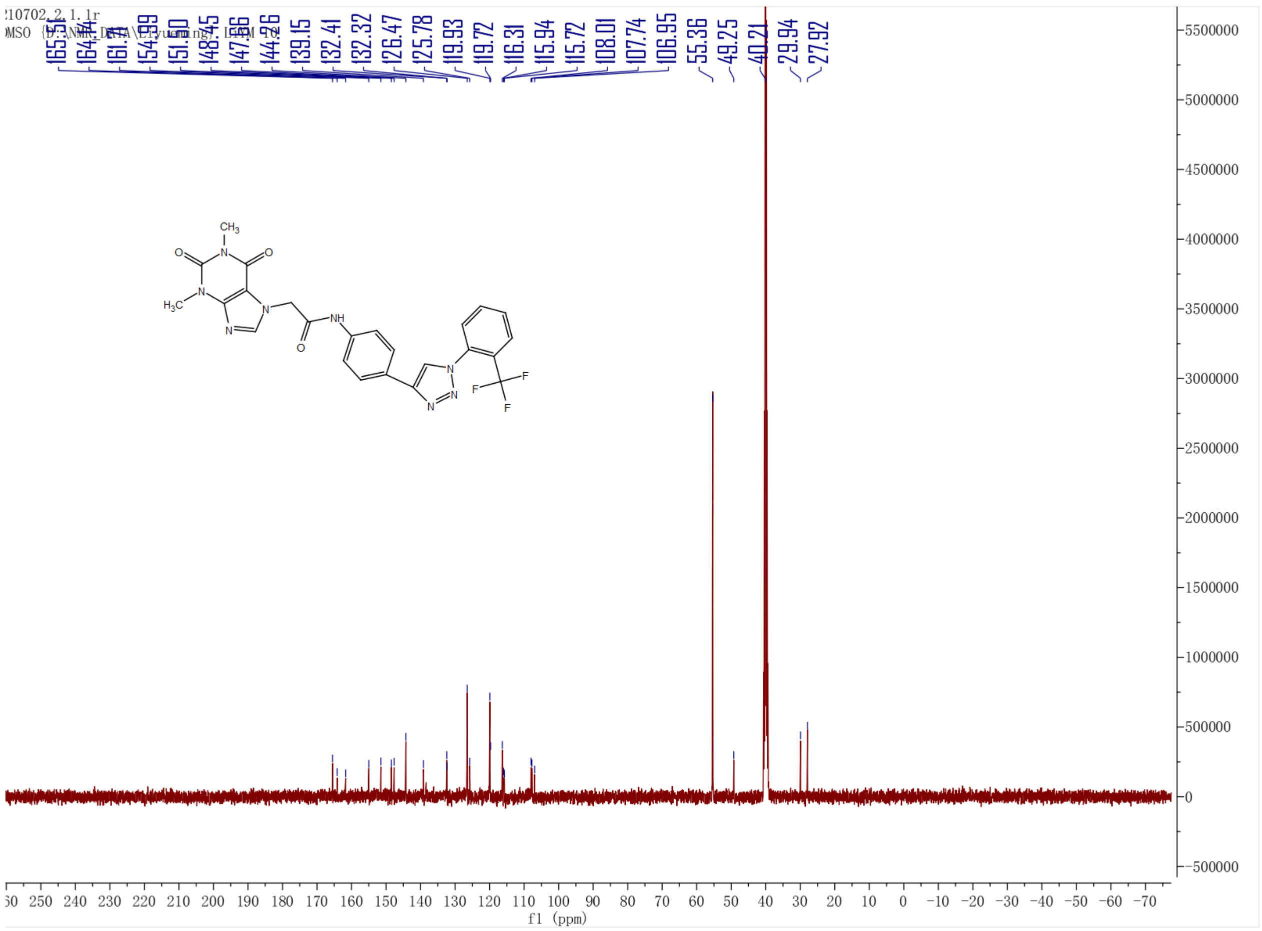

Figure S15-1. ^{1}H NMR spectrum (400MHz, DMSO-d$_6$) of compound D15

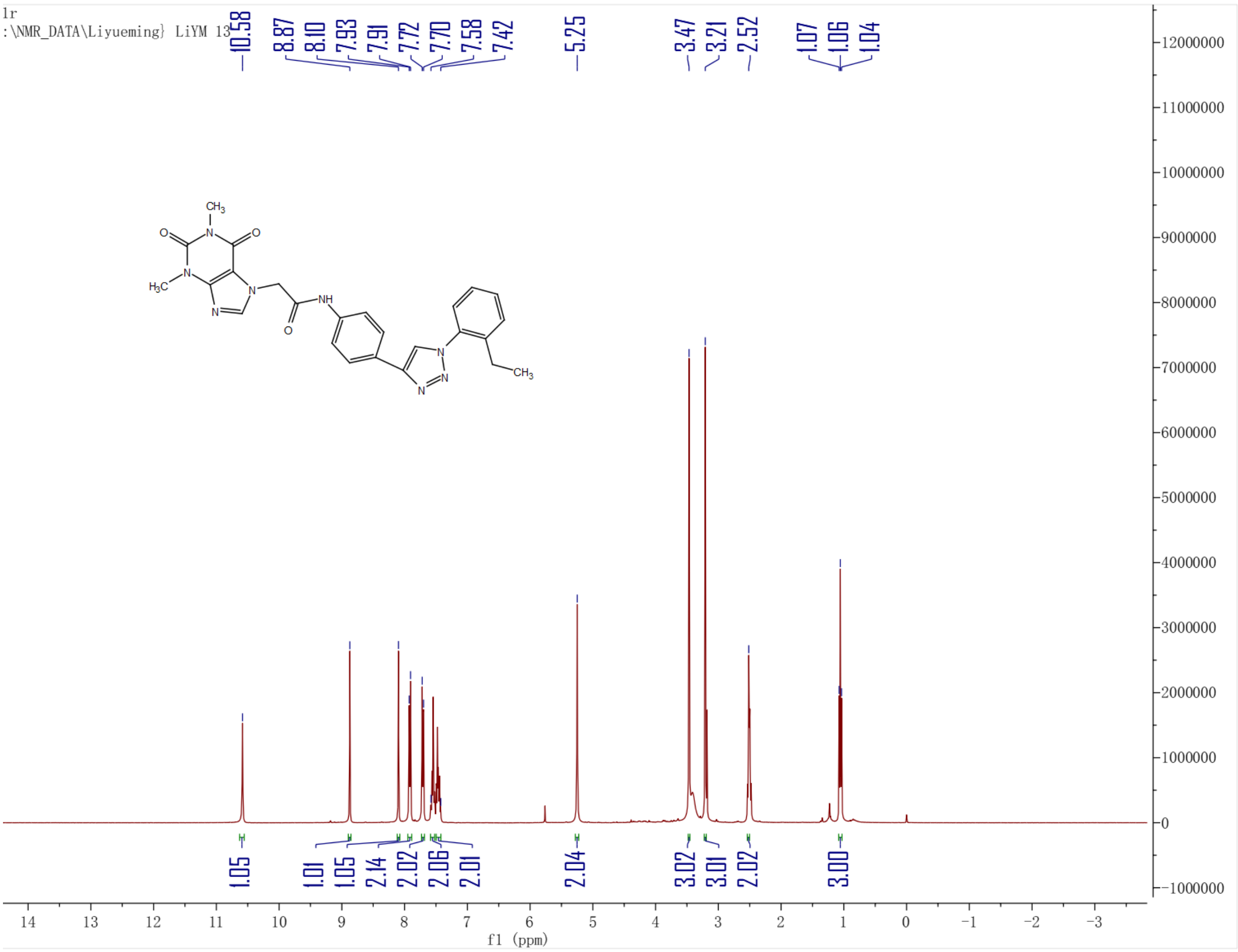

Figure S15-2. ^{13}C NMR spectrum (100MHz, DMSO-d_6) of compound D15

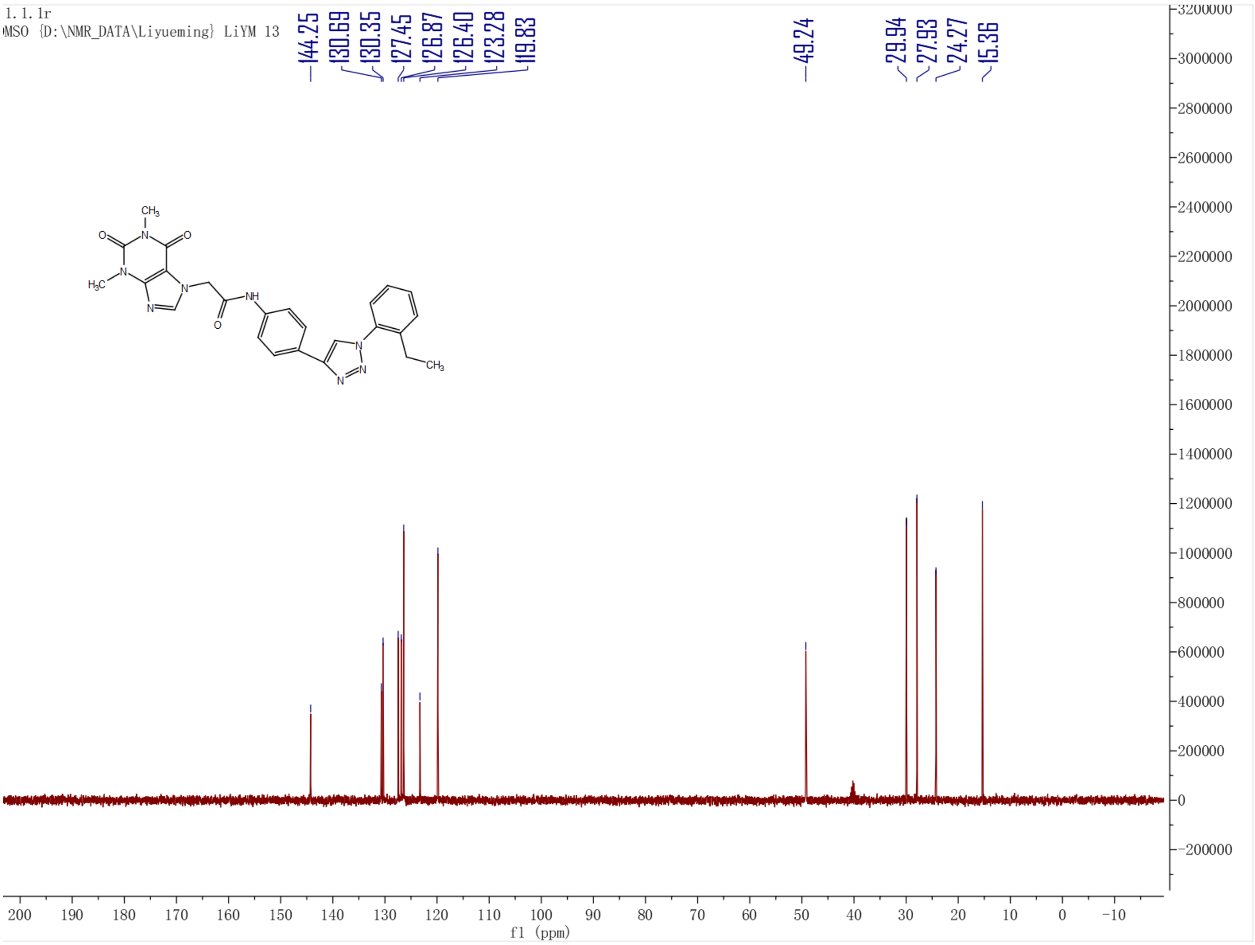

Figure S16-1. ^{1}H NMR spectrum (400MHz, DMSO-d_6) of compound D16

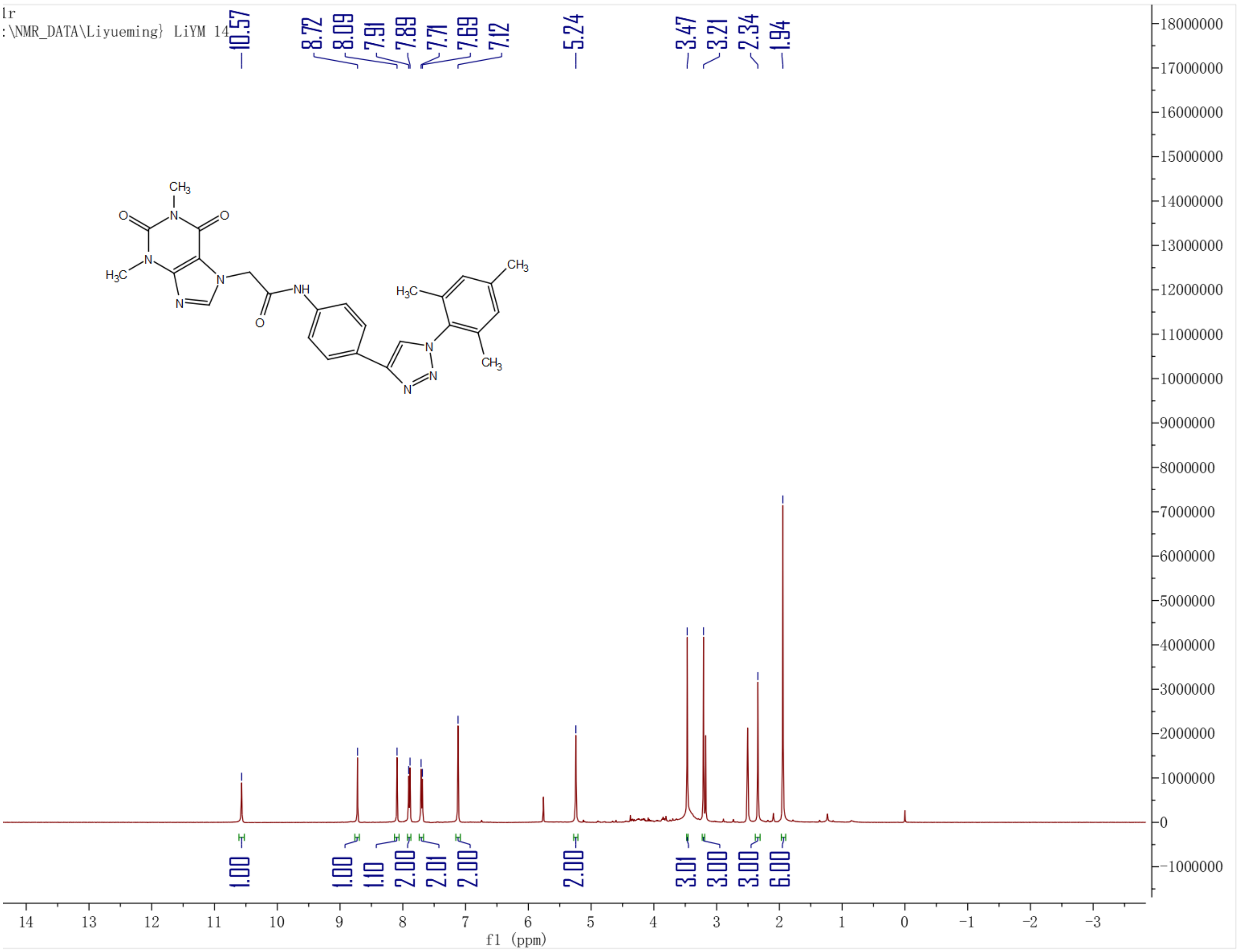

Figure S16-2. ^{13}C NMR spectrum (100MHz, DMSO-d$_6$) of compound D16

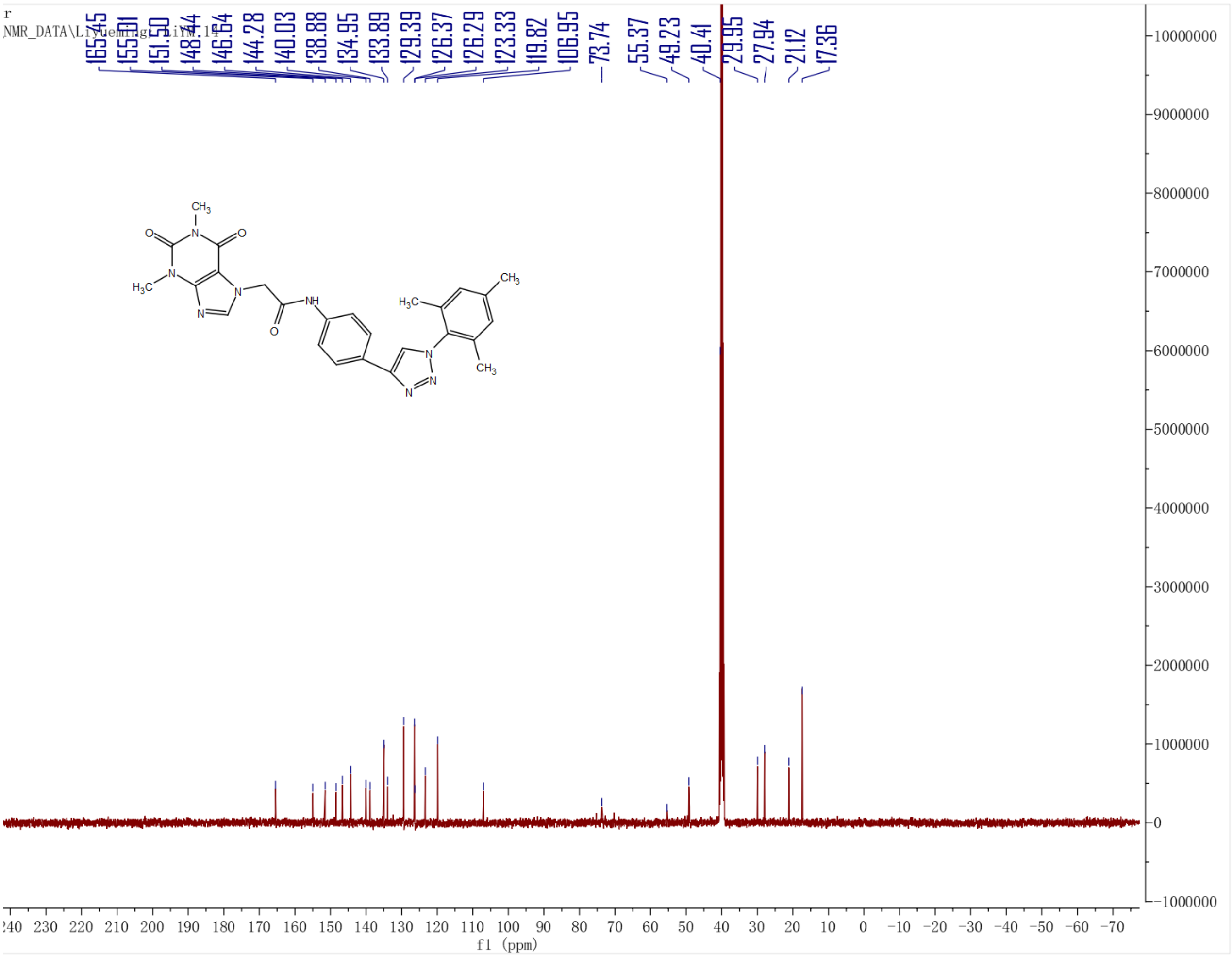

Figure S17-1. ^{1}H NMR spectrum (400MHz, DMSO-d$_6$) of compound D17

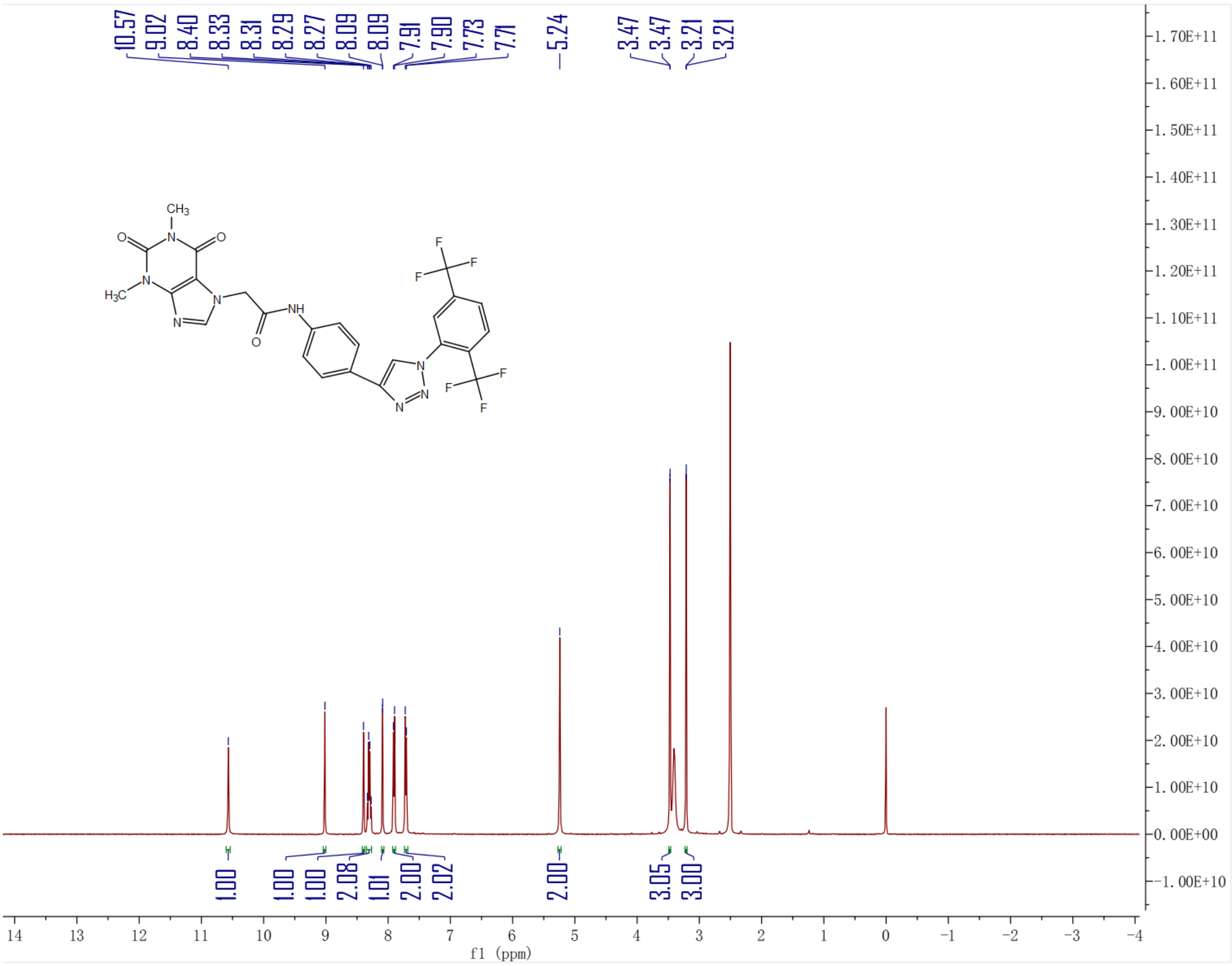

Figure S17-2. ^{13}C NMR spectrum (100MHz, DMSO-d$_6$) of compound D17

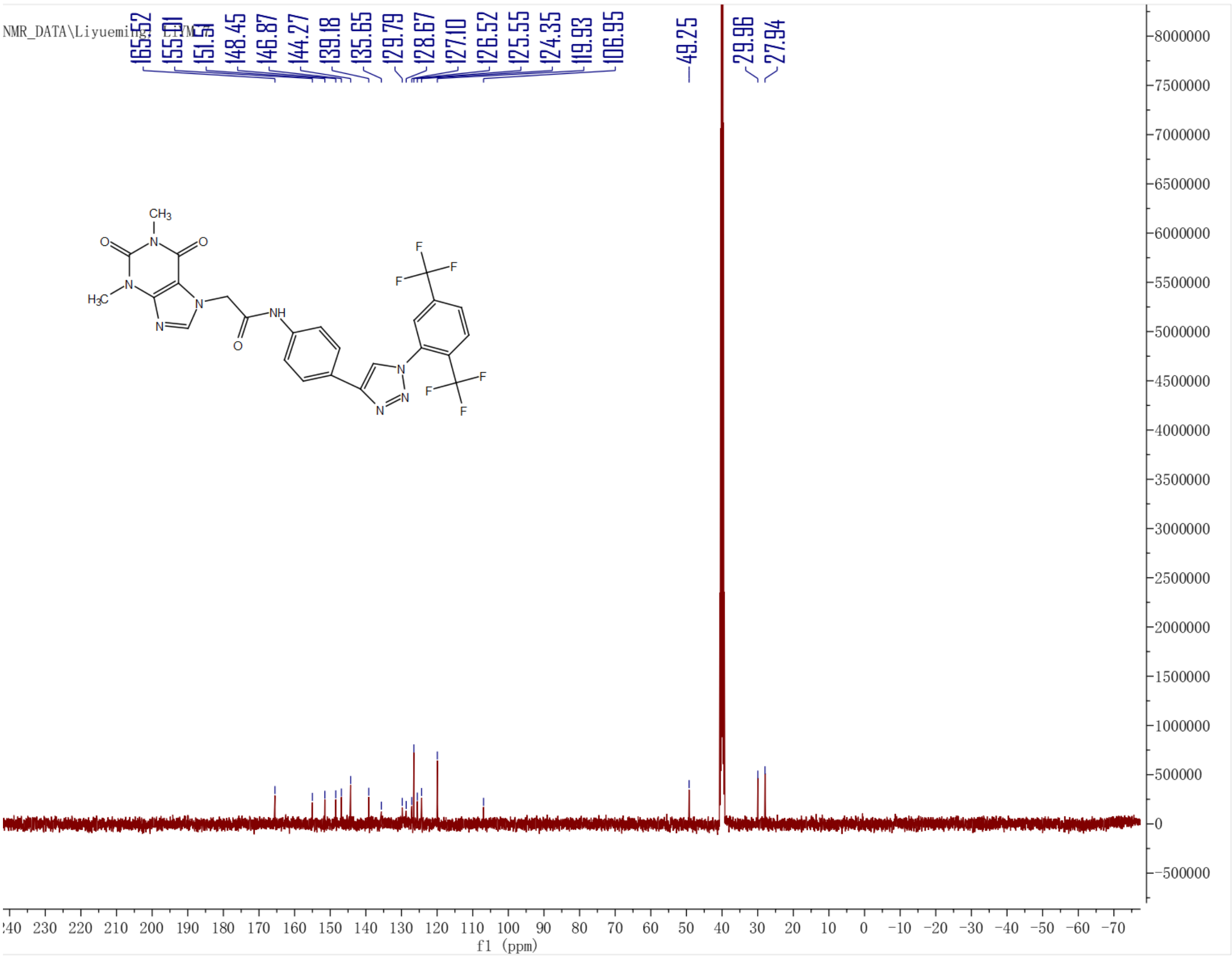

Figure S18-1. [1]**H NMR spectrum (400MHz, DMSO-d$_6$) of compound D18**

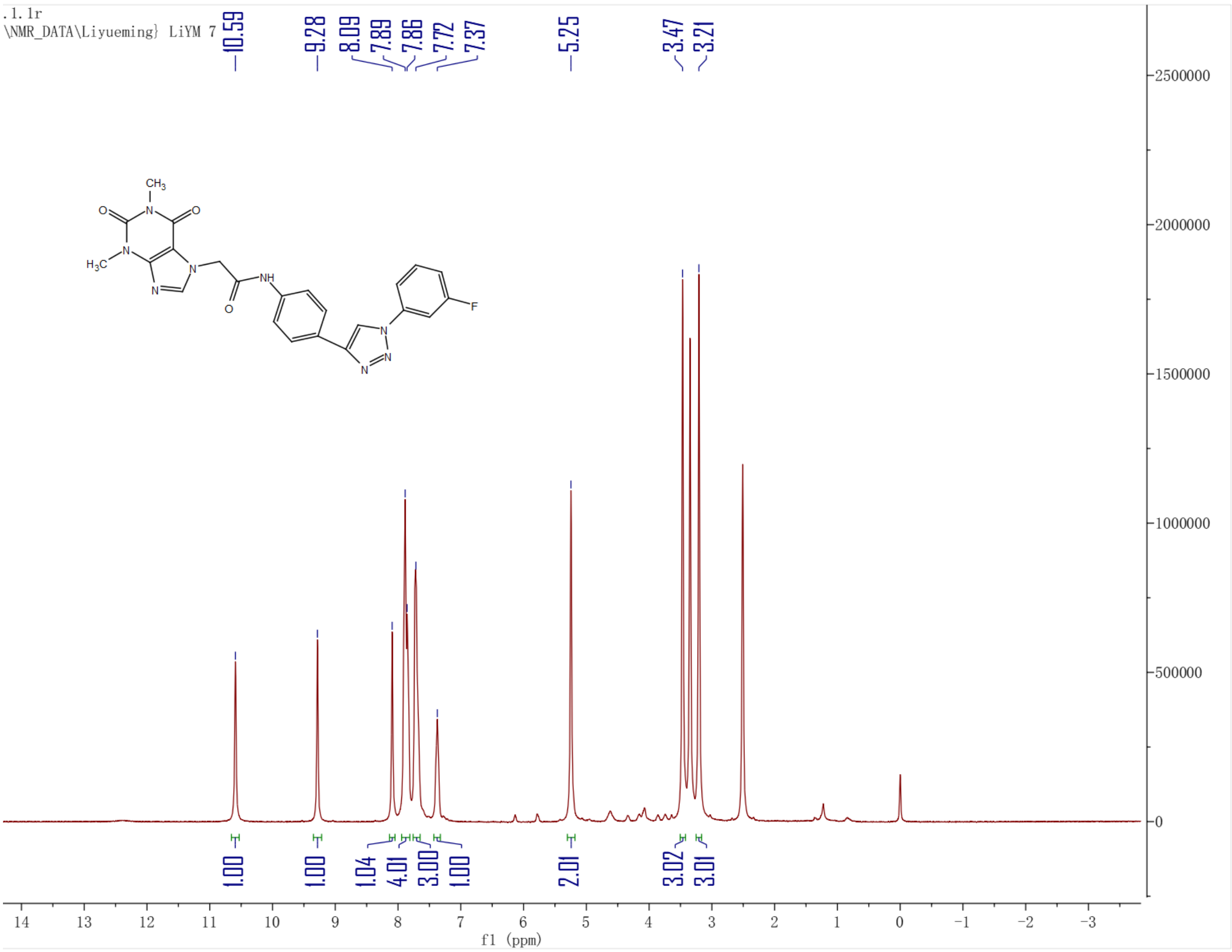

Figure S18-2. ^{13}C NMR spectrum (100MHz, DMSO-d$_6$) of compound D18

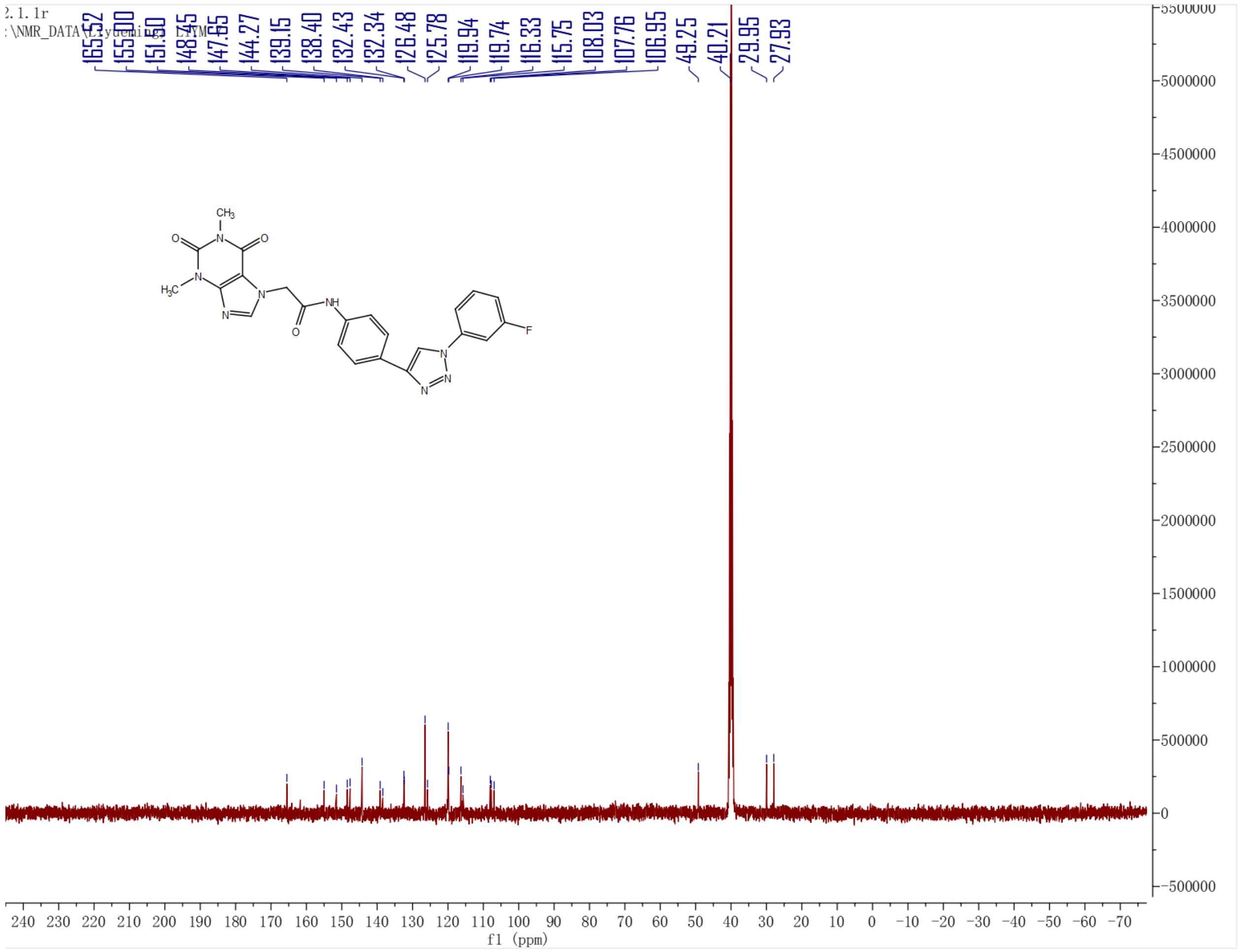

Figure S19-1. ^{1}H NMR spectrum (400MHz, DMSO-d$_6$) of compound D19

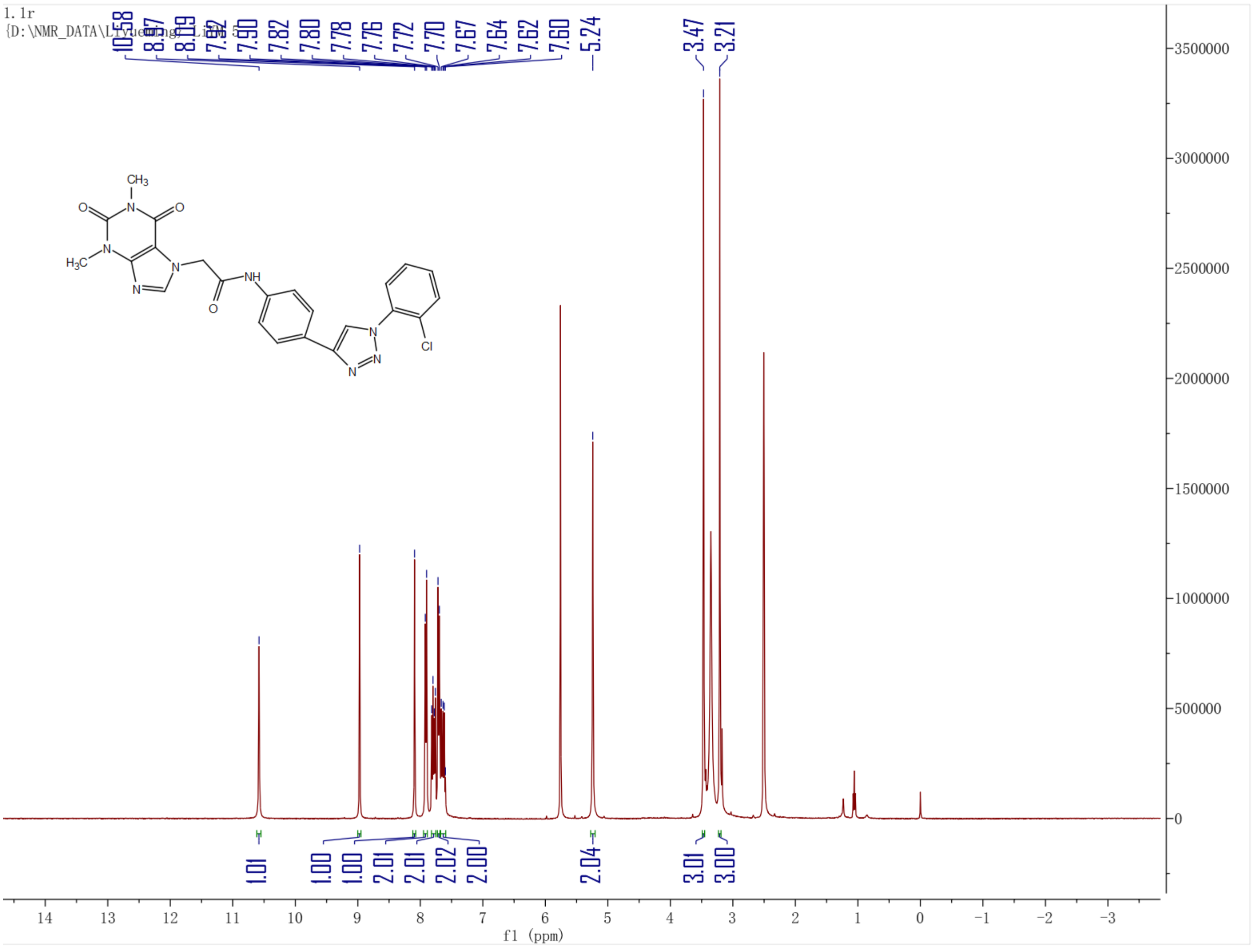

Figure S19-2. ^{13}C NMR spectrum (100MHz, DMSO-d_6) of compound D19

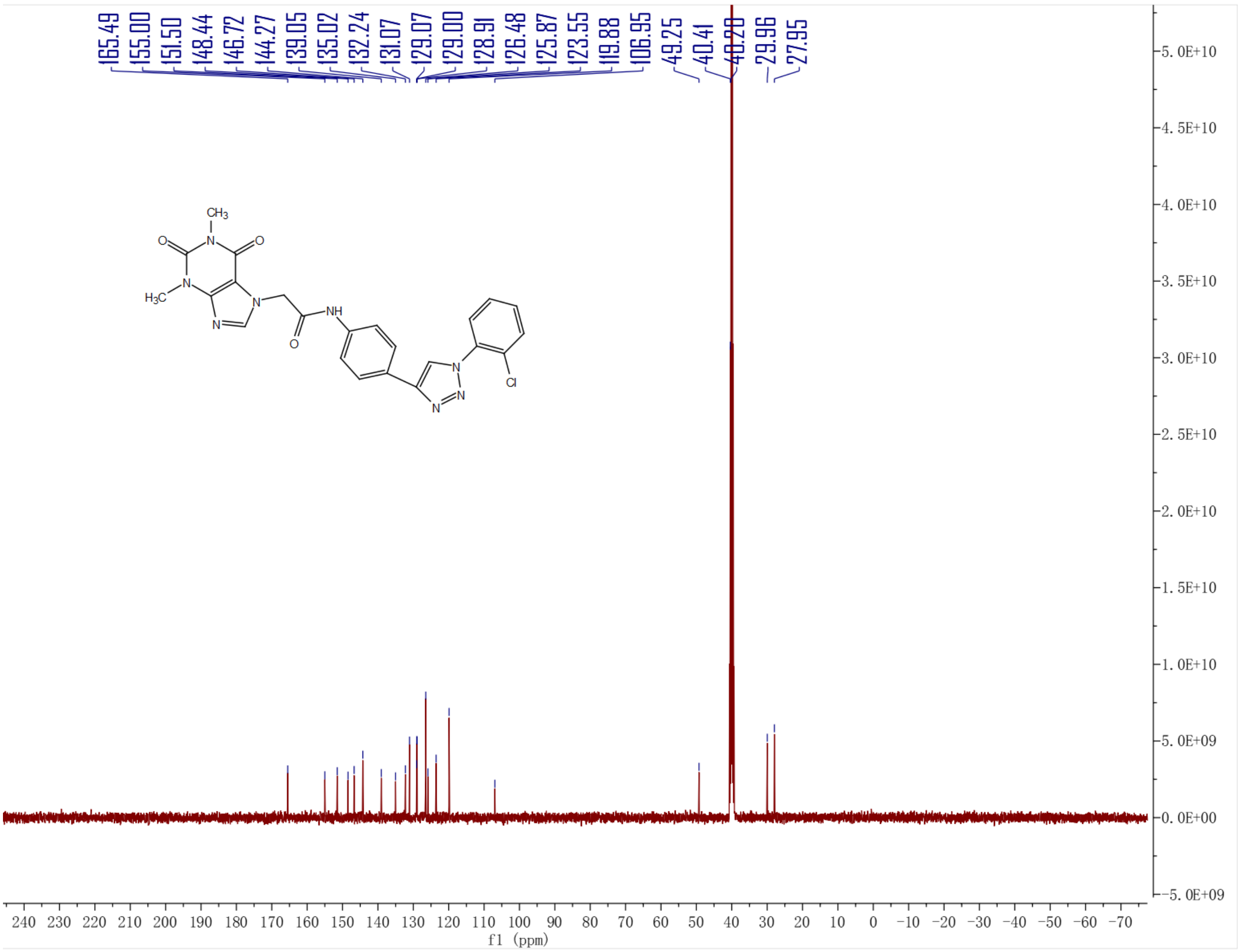

Figure S20-1. ^{1}H NMR spectrum (400MHz, DMSO-d$_6$) of compound D20

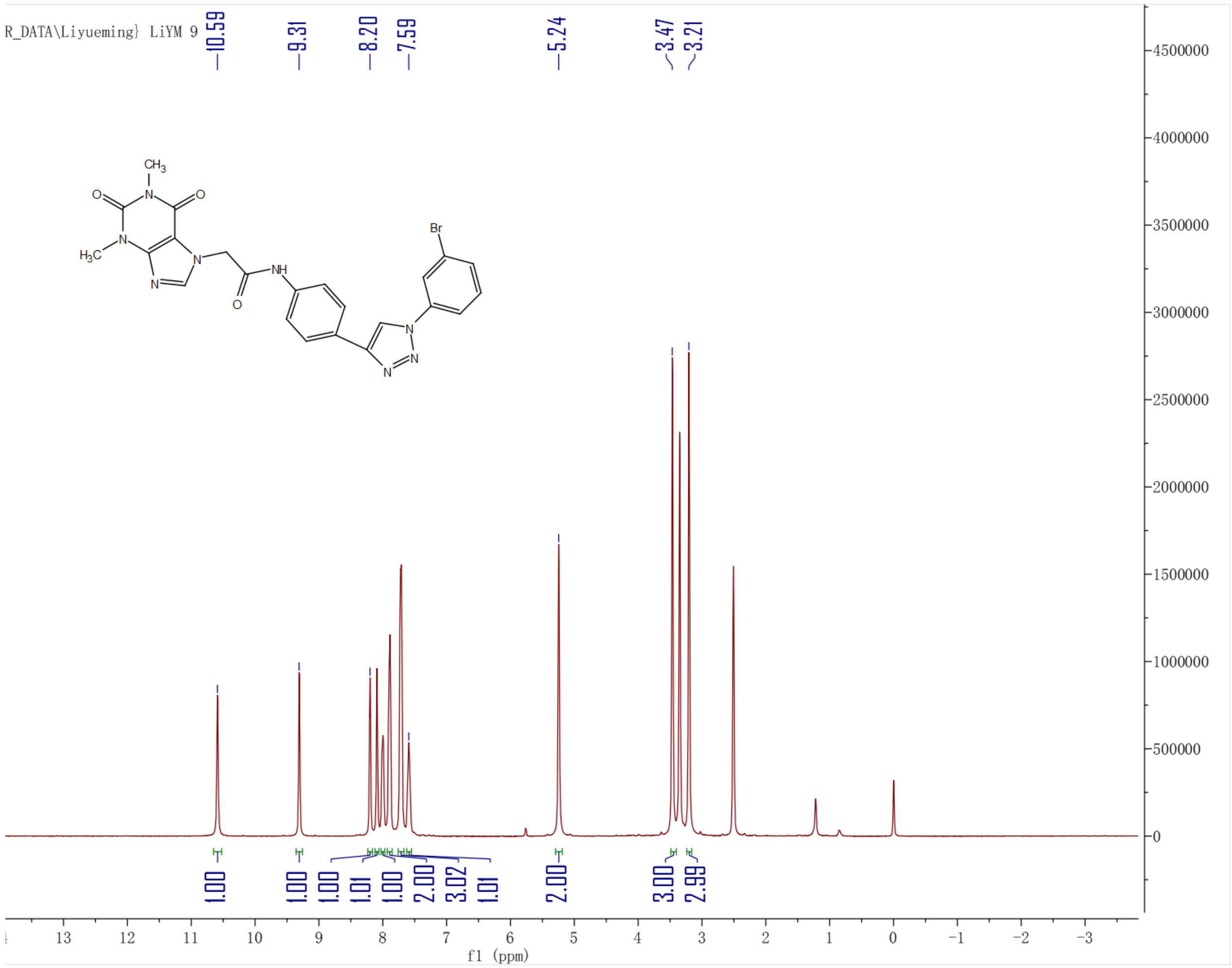

Figure S20-2. ^{13}C NMR spectrum (100MHz, DMSO-d$_6$) of compound D20

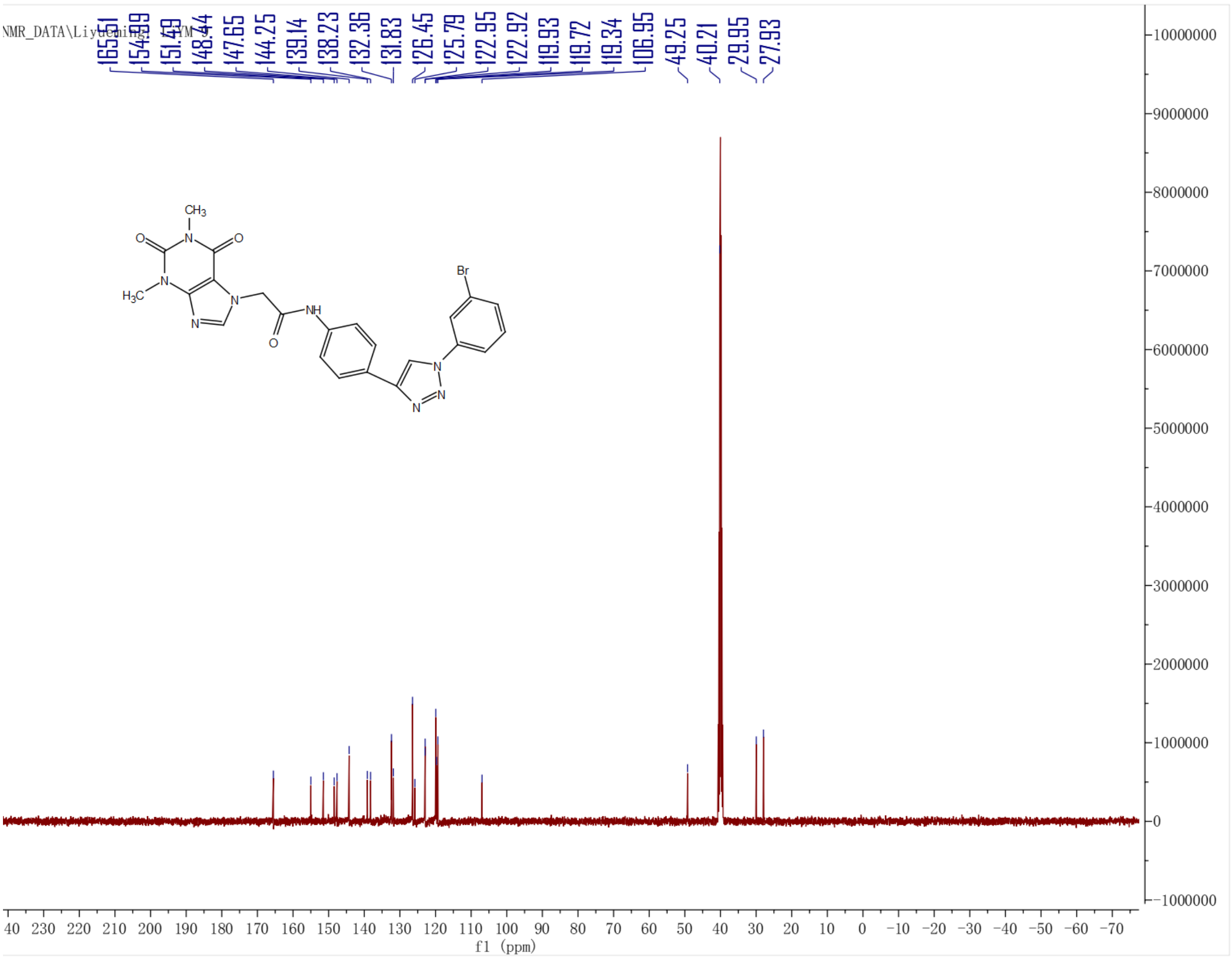

Figure S21-1. ^{1}H NMR spectrum (400MHz, DMSO-d$_6$) of compound D21

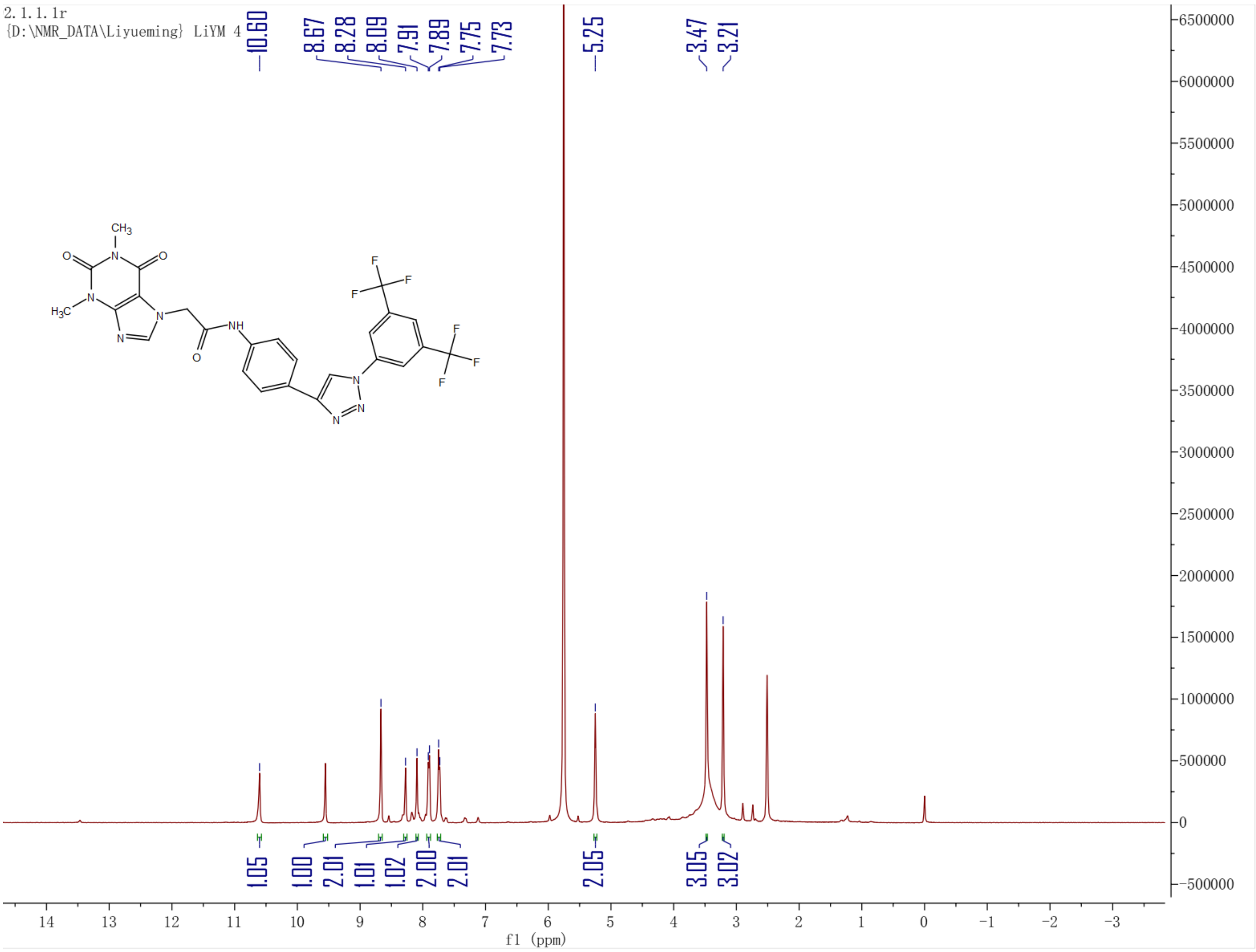

Figure S21-2. ^{13}C NMR spectrum (100MHz, DMSO-d_6) of compound D21

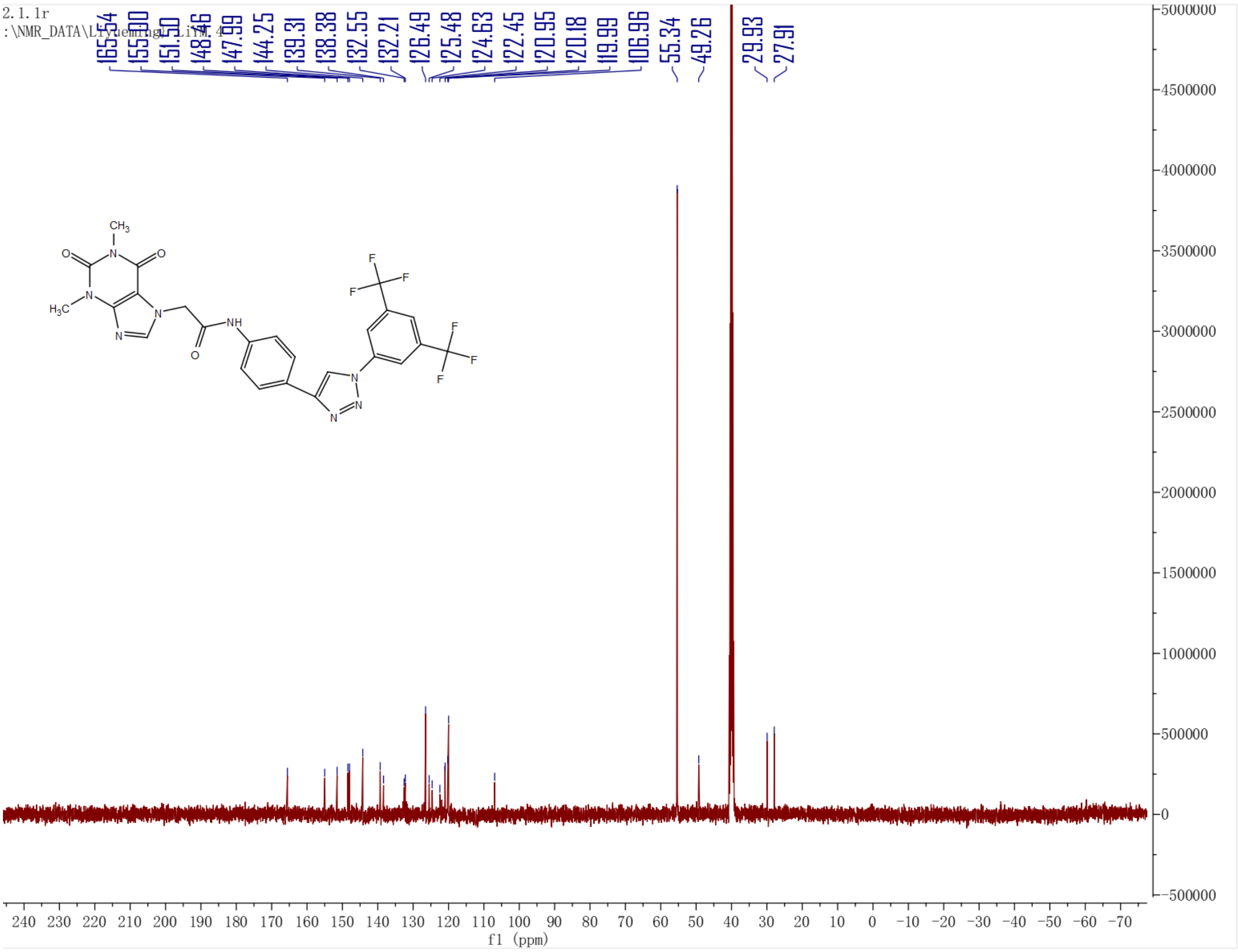

Figure S22-1. ^{1}H NMR spectrum (400MHz, DMSO-d_6) of compound D22

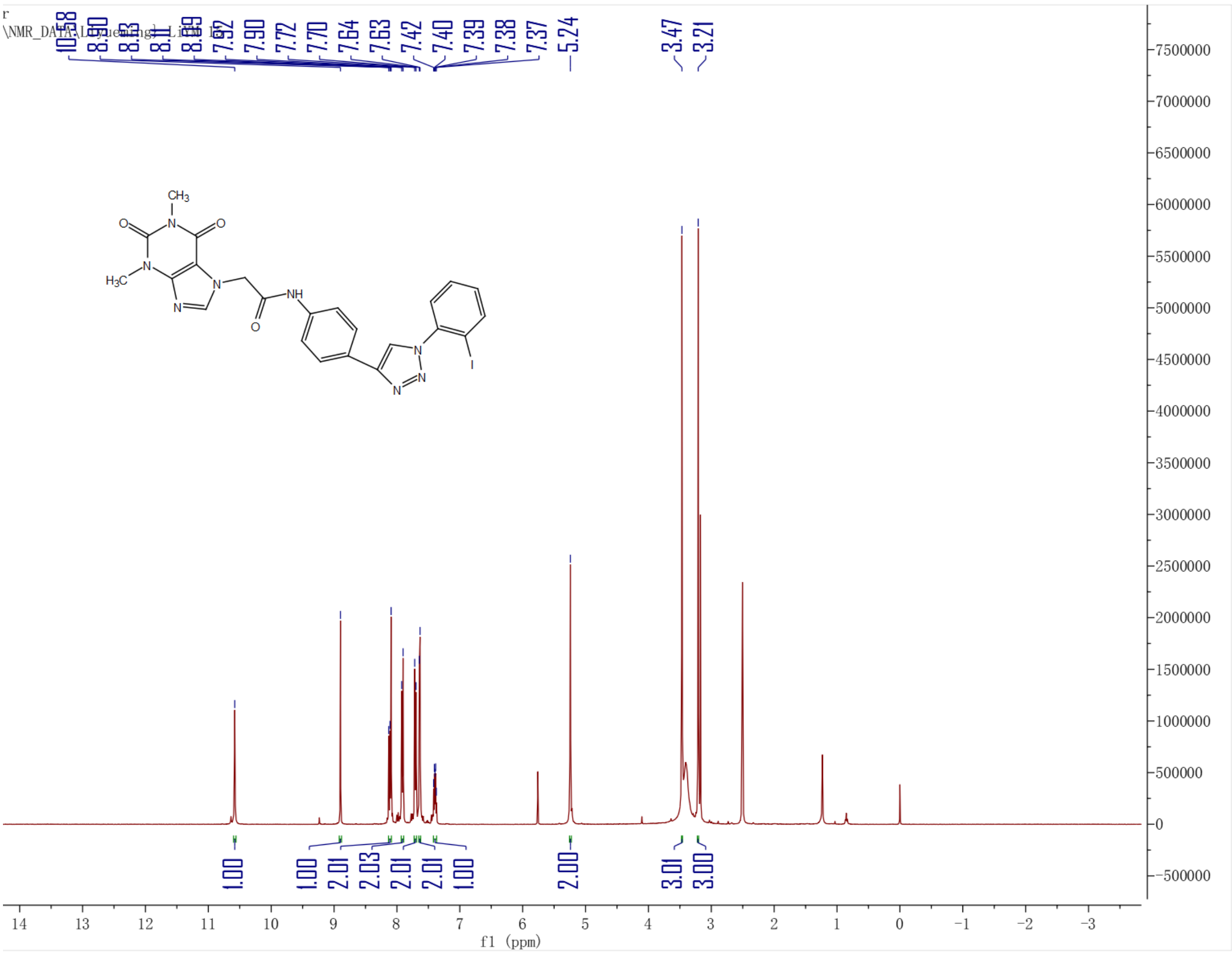

Figure S22-2. ^{13}C NMR spectrum (100MHz, DMSO-d$_6$) of compound D22

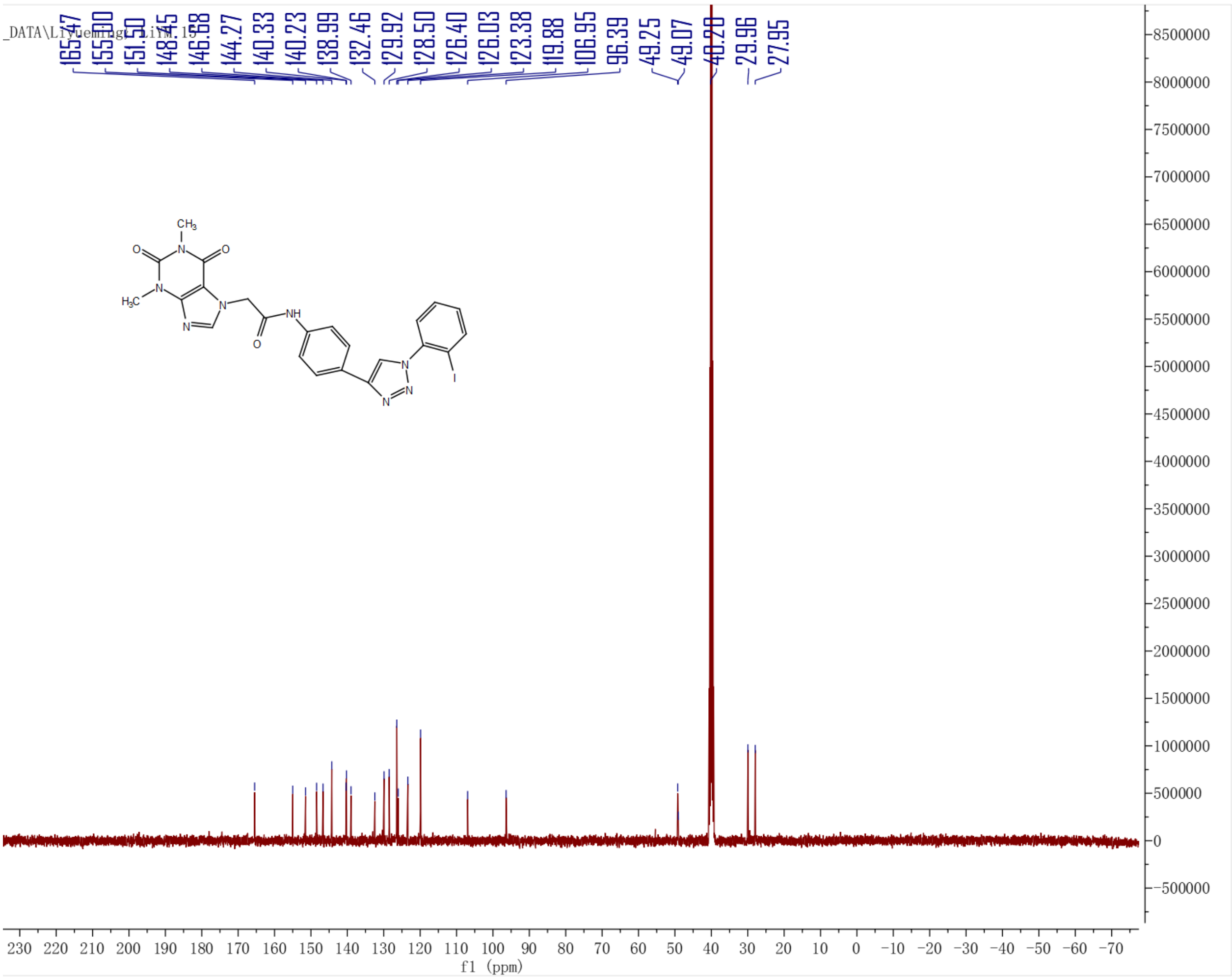

Figure S23-1. ^{1}H NMR spectrum (400MHz, DMSO-d$_6$) of compound D23

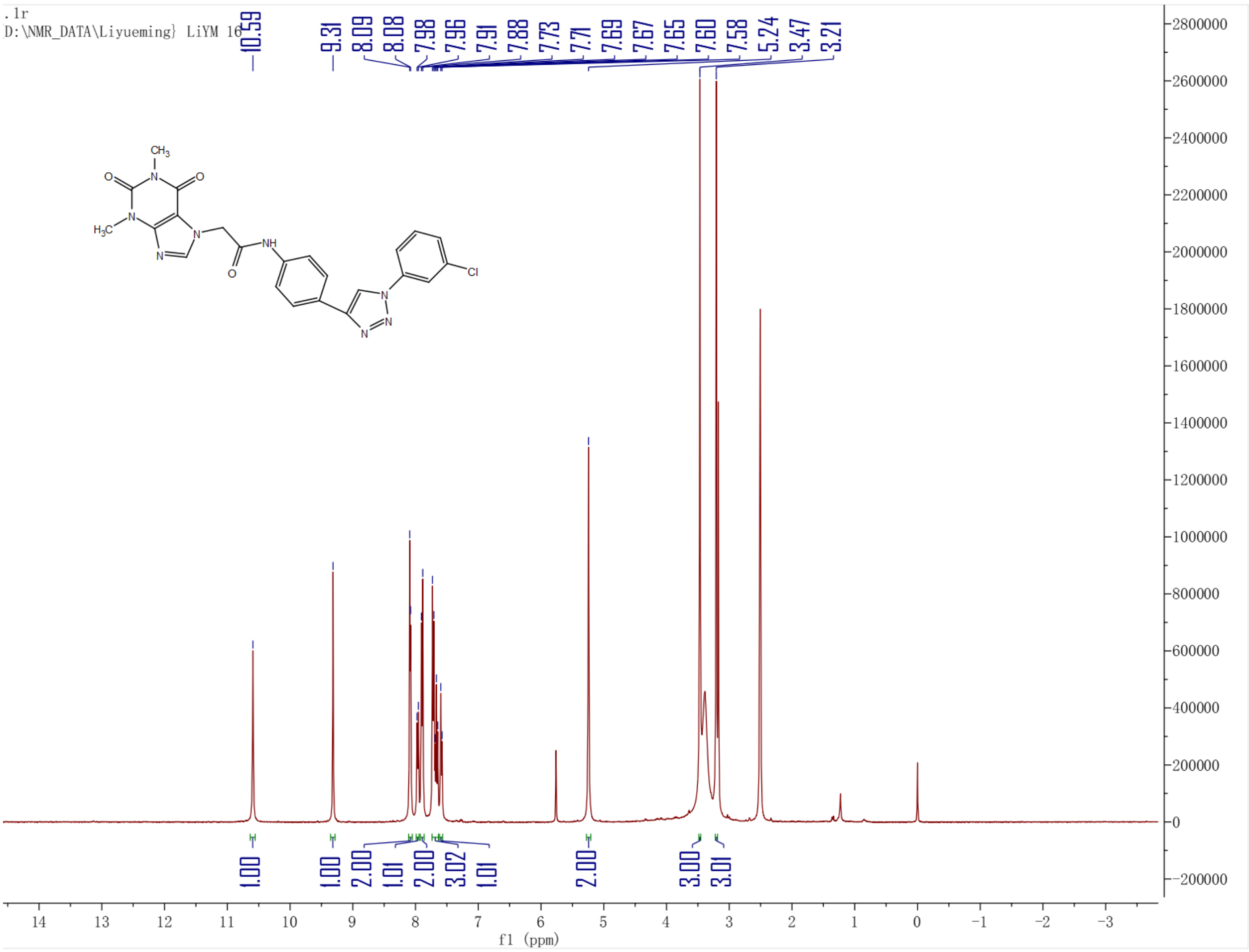

Figure S23-2. ^{13}C NMR spectrum (100MHz, DMSO-d_6) of compound D23

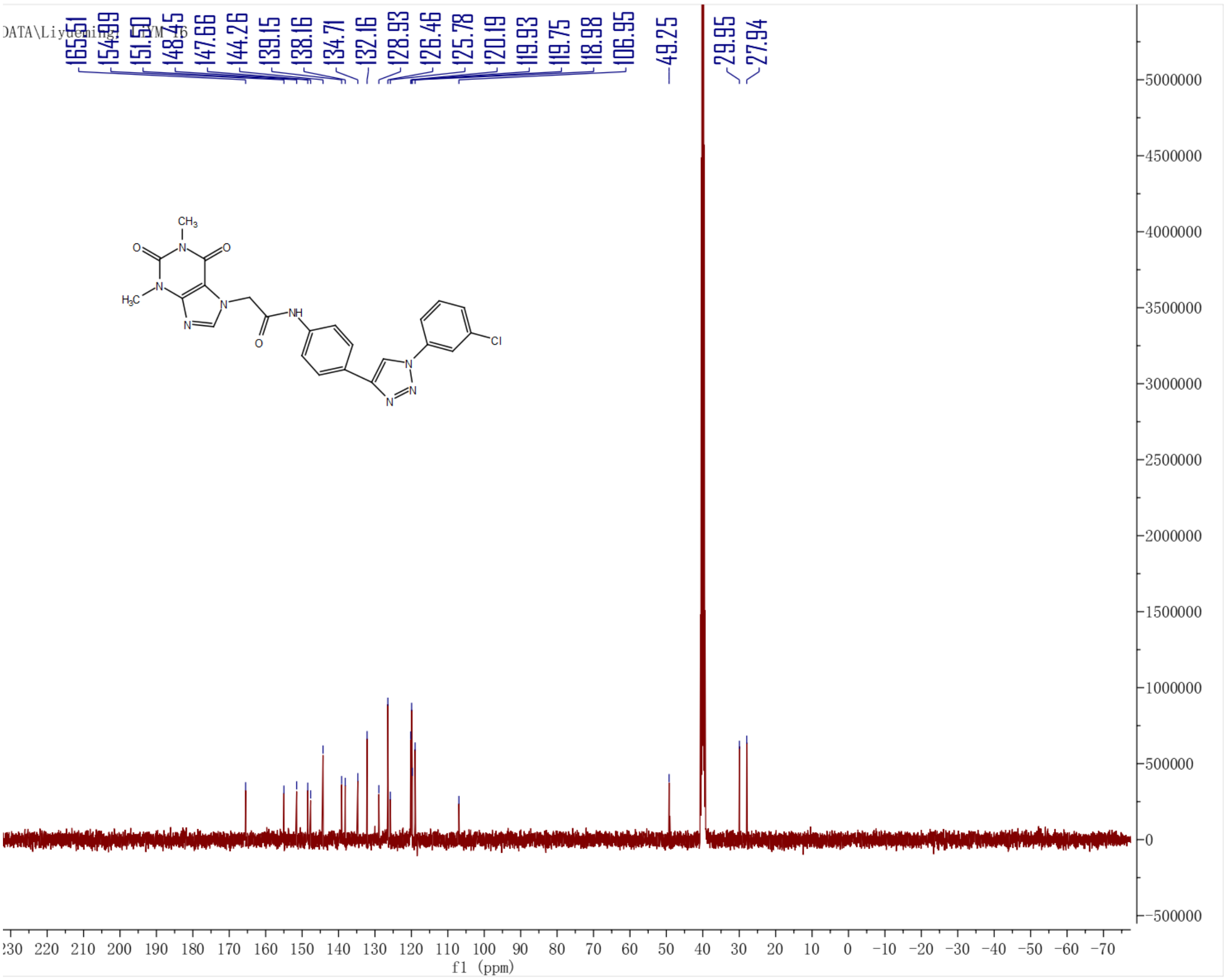

Figure S24-1. ^{1}H NMR spectrum (400MHz, DMSO-d$_6$) of compound D24

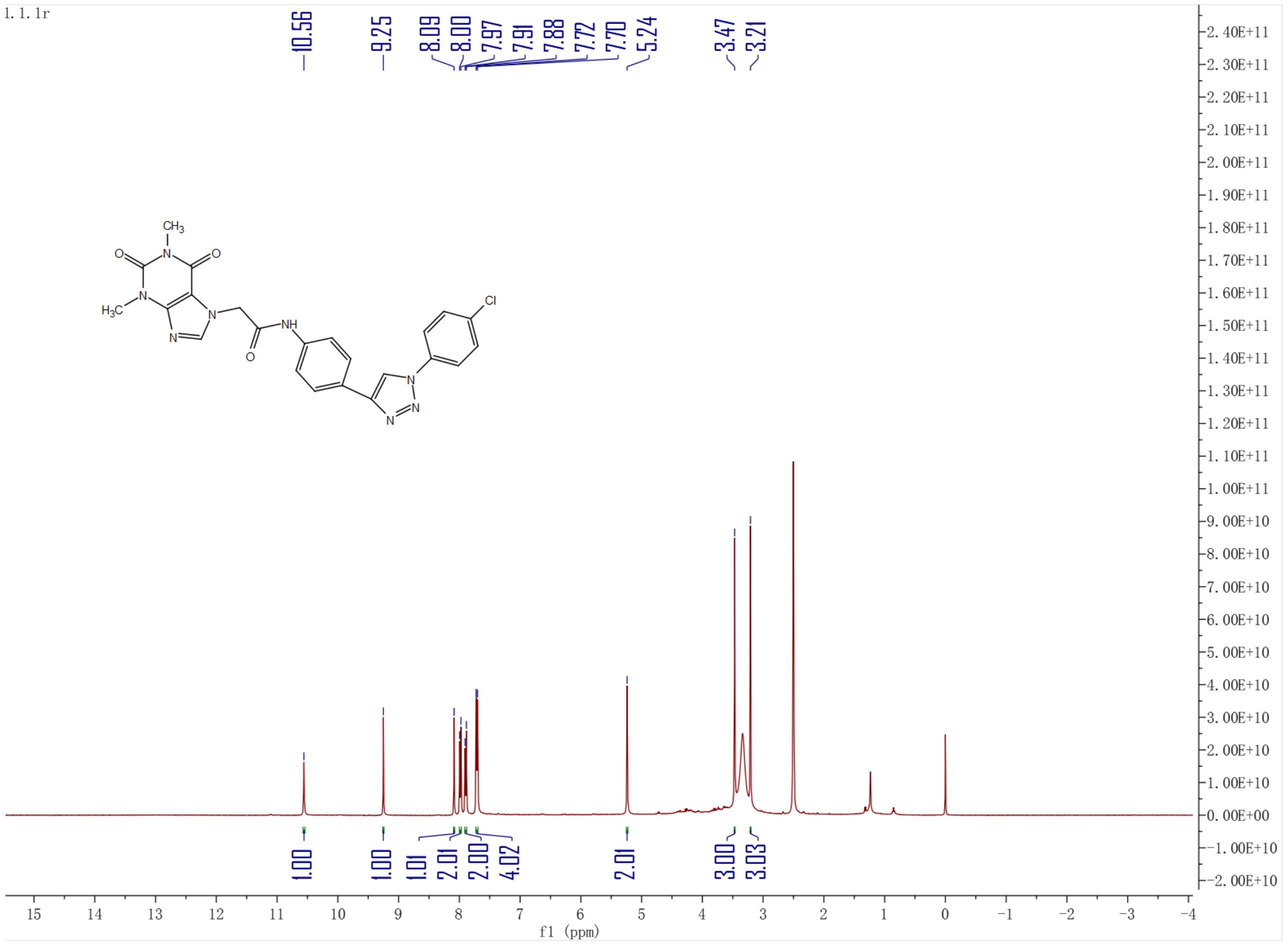

Figure S24-2. ^{13}C NMR spectrum (100MHz, DMSO-d_6) of compound D24

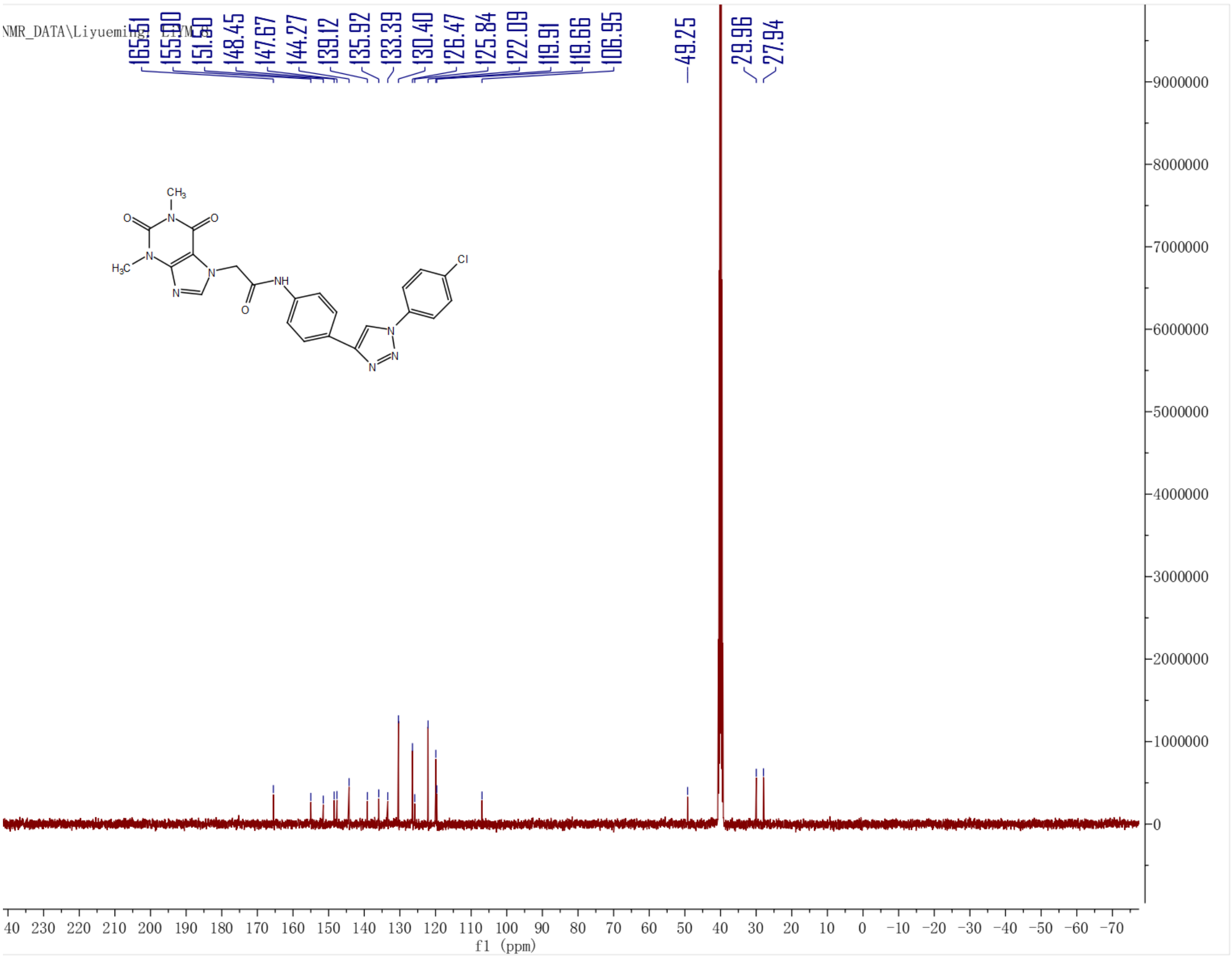

Figure S25-1. ^{1}H NMR spectrum (400MHz, DMSO-d_6) of compound D25

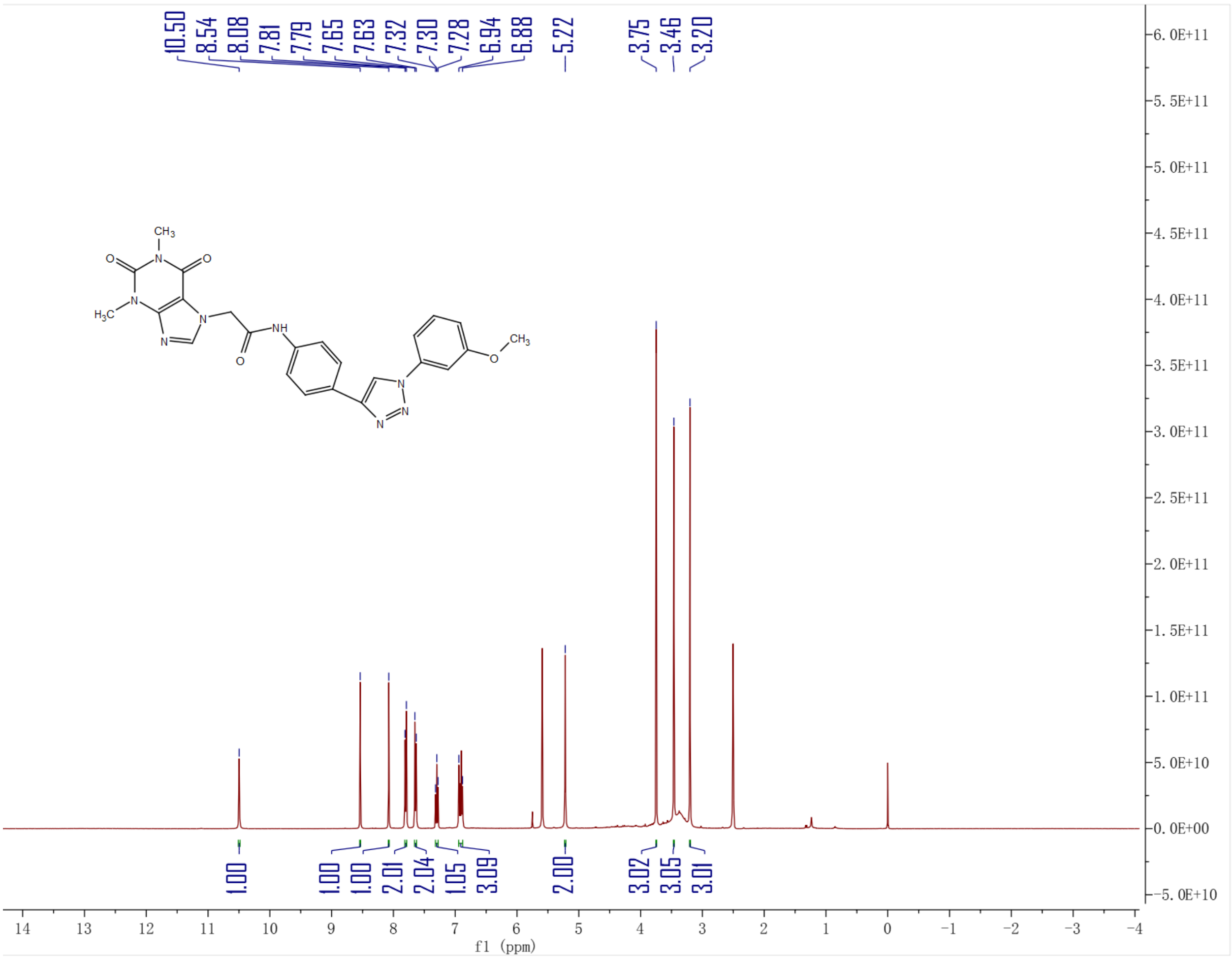

Figure S25-2. ^{13}C NMR spectrum (100MHz, DMSO-d$_6$) of compound D25

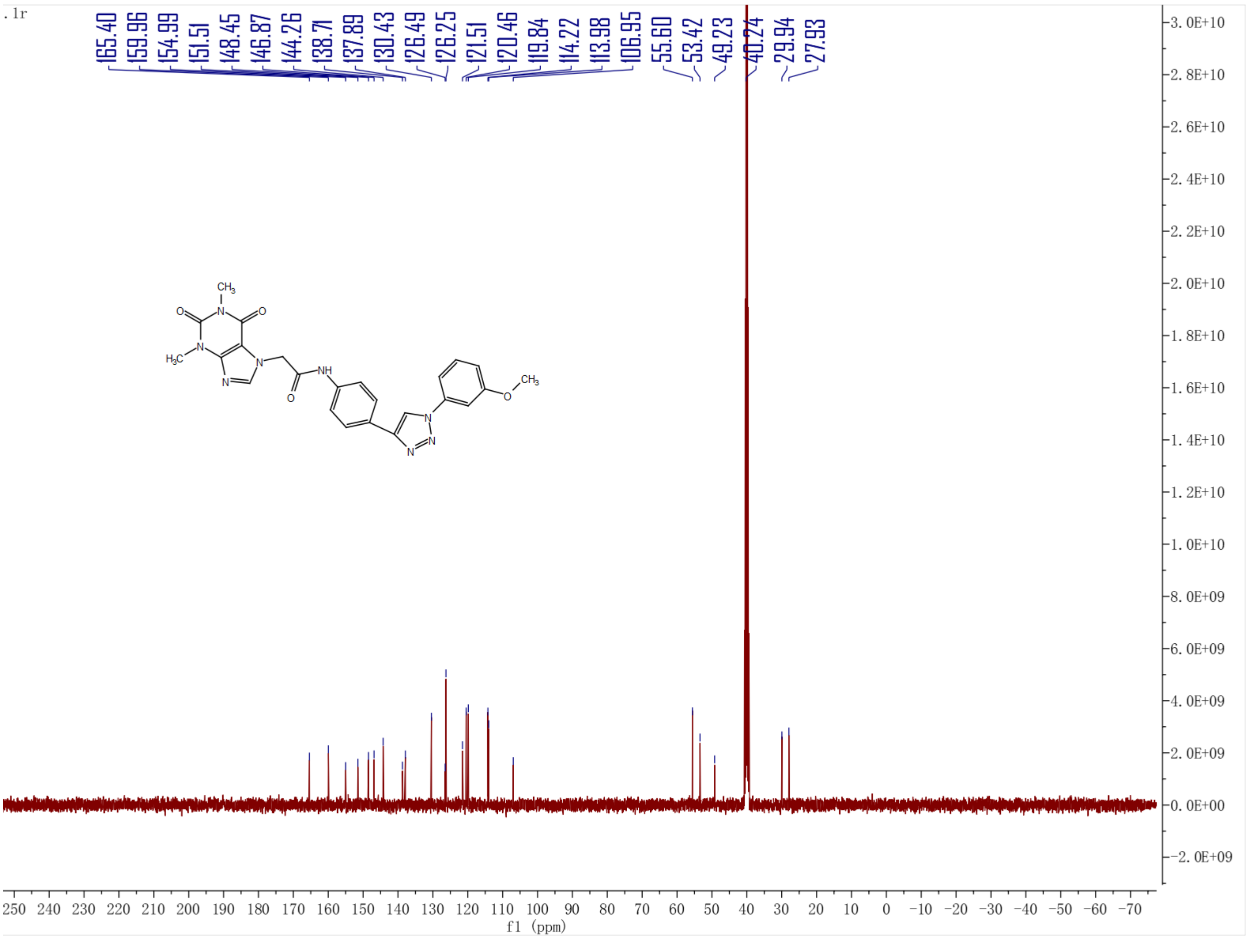

Figure S26-1. ^{1}H NMR spectrum (400MHz, DMSO-d$_6$) of compound D26

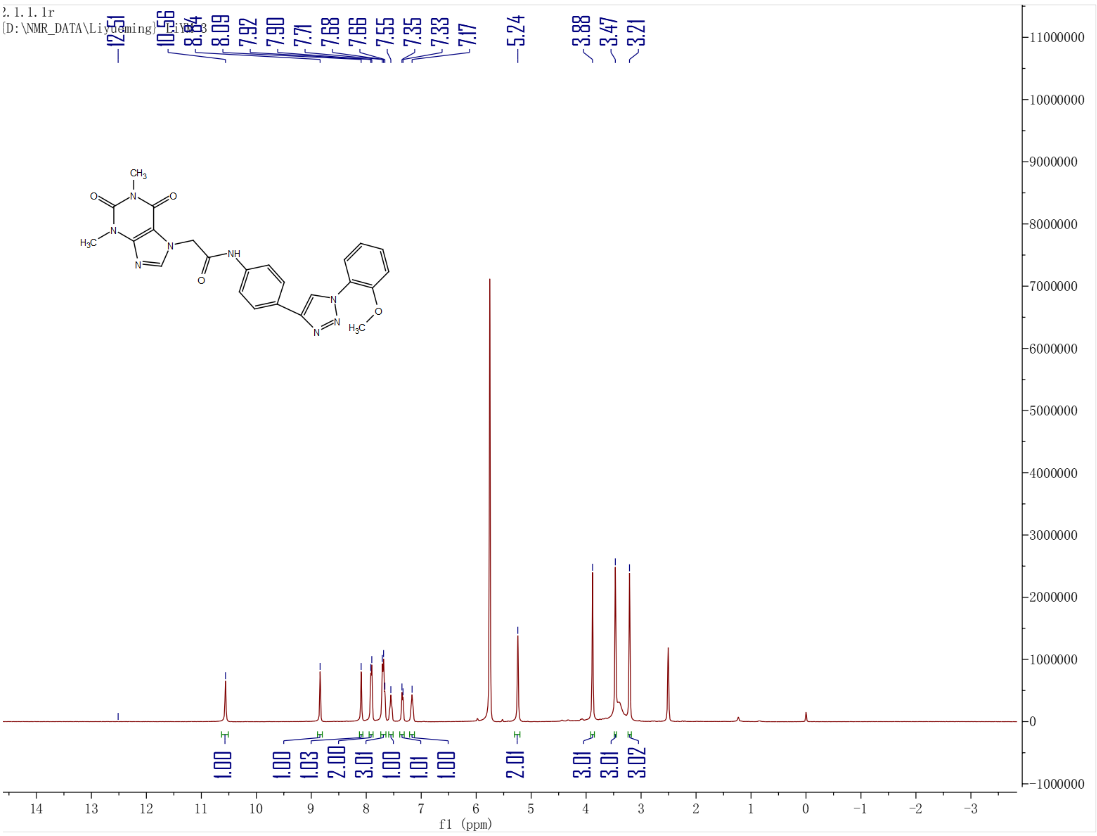

Figure S26-2. ^{13}C NMR spectrum (100MHz, DMSO-d_6) of compound D26

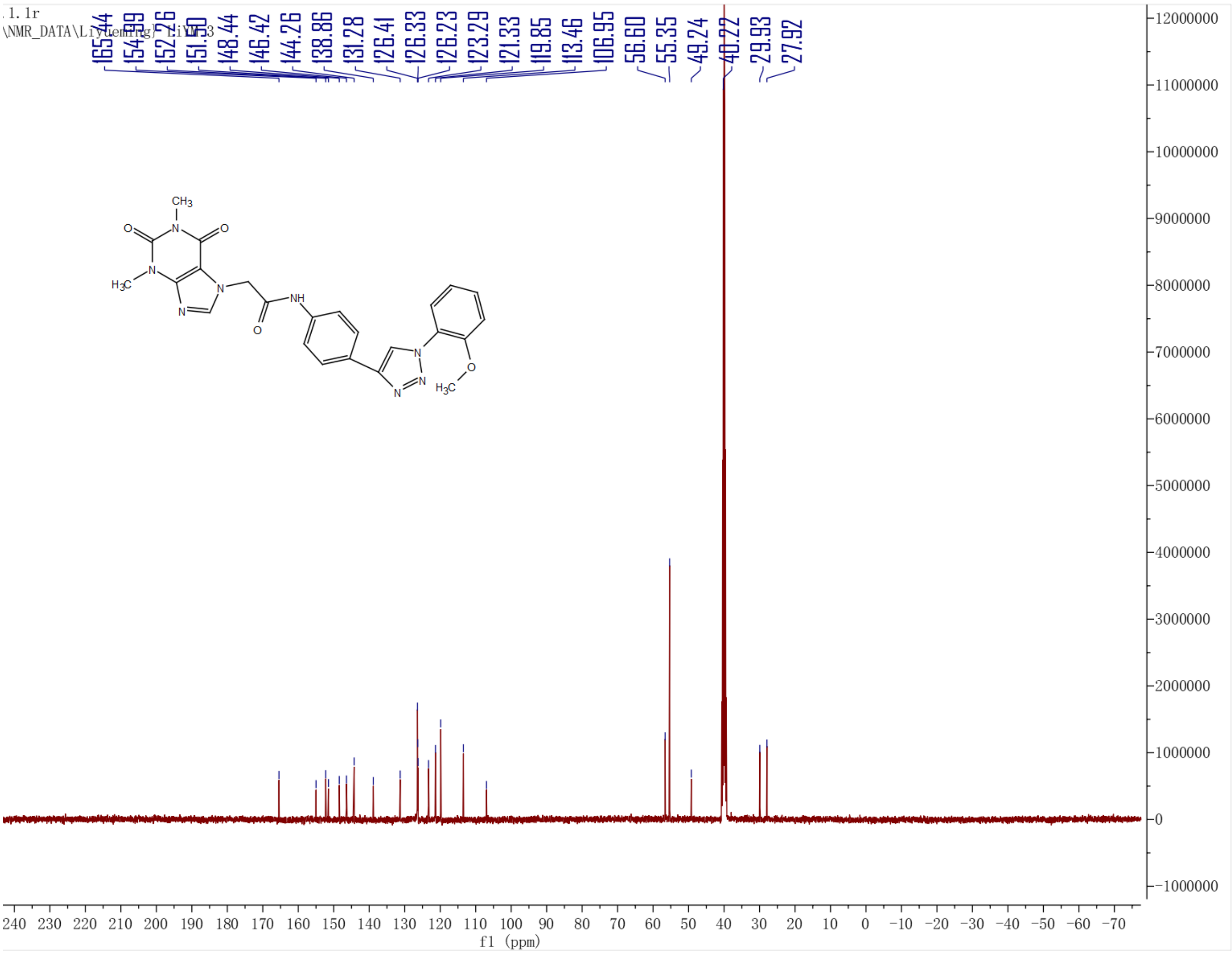

Figure S27-1. ^{1}H NMR spectrum (400MHz, DMSO-d_6) of compound D27

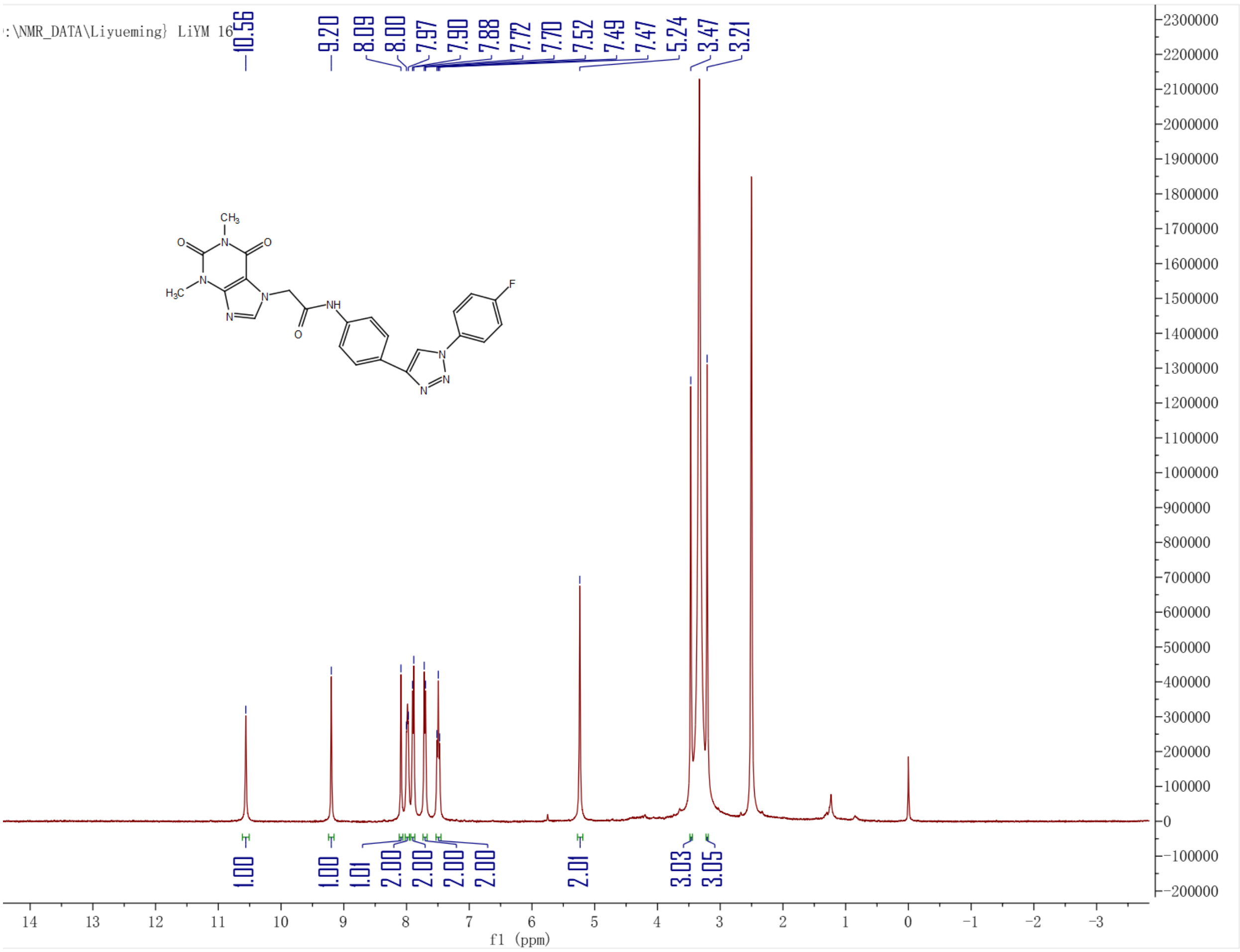

Figure S27-2. ^{13}C NMR spectrum (100MHz, DMSO-d_6) of compound D27

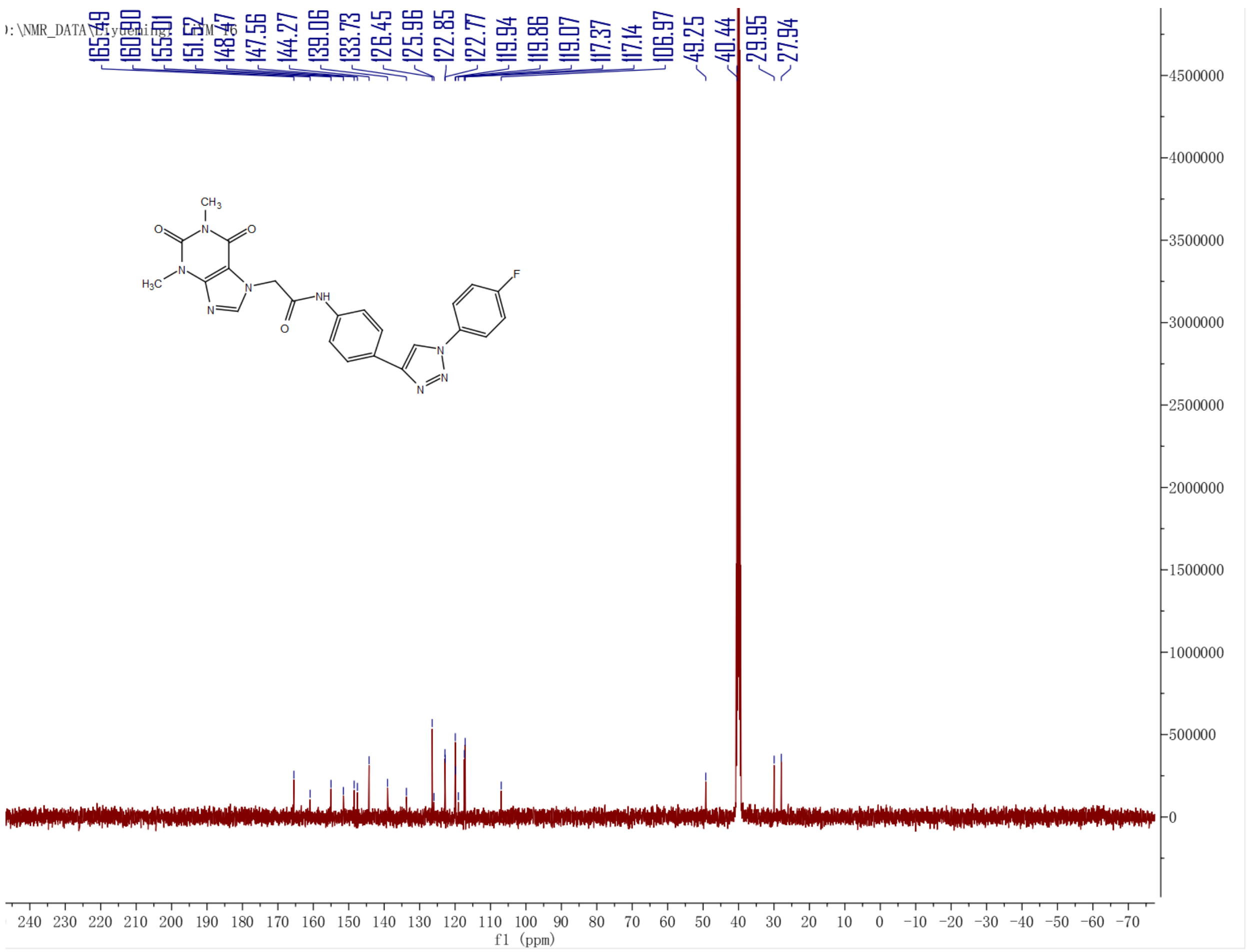

Figure S28-1. ^{1}H NMR spectrum (400MHz, DMSO-d$_6$) of compound D28

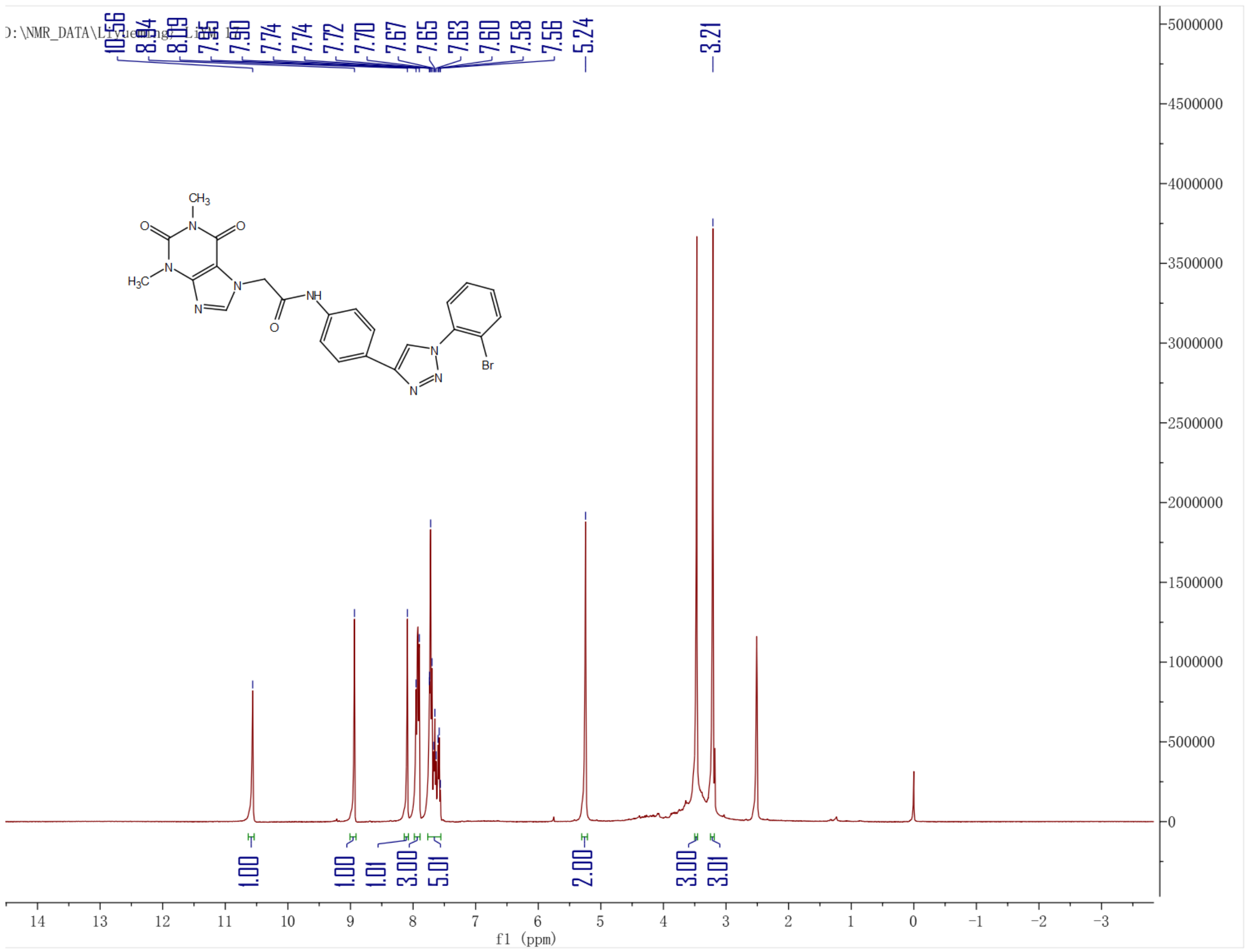

Figure S28-2. ^{13}C NMR spectrum (100MHz, DMSO-d$_6$) of compound D28

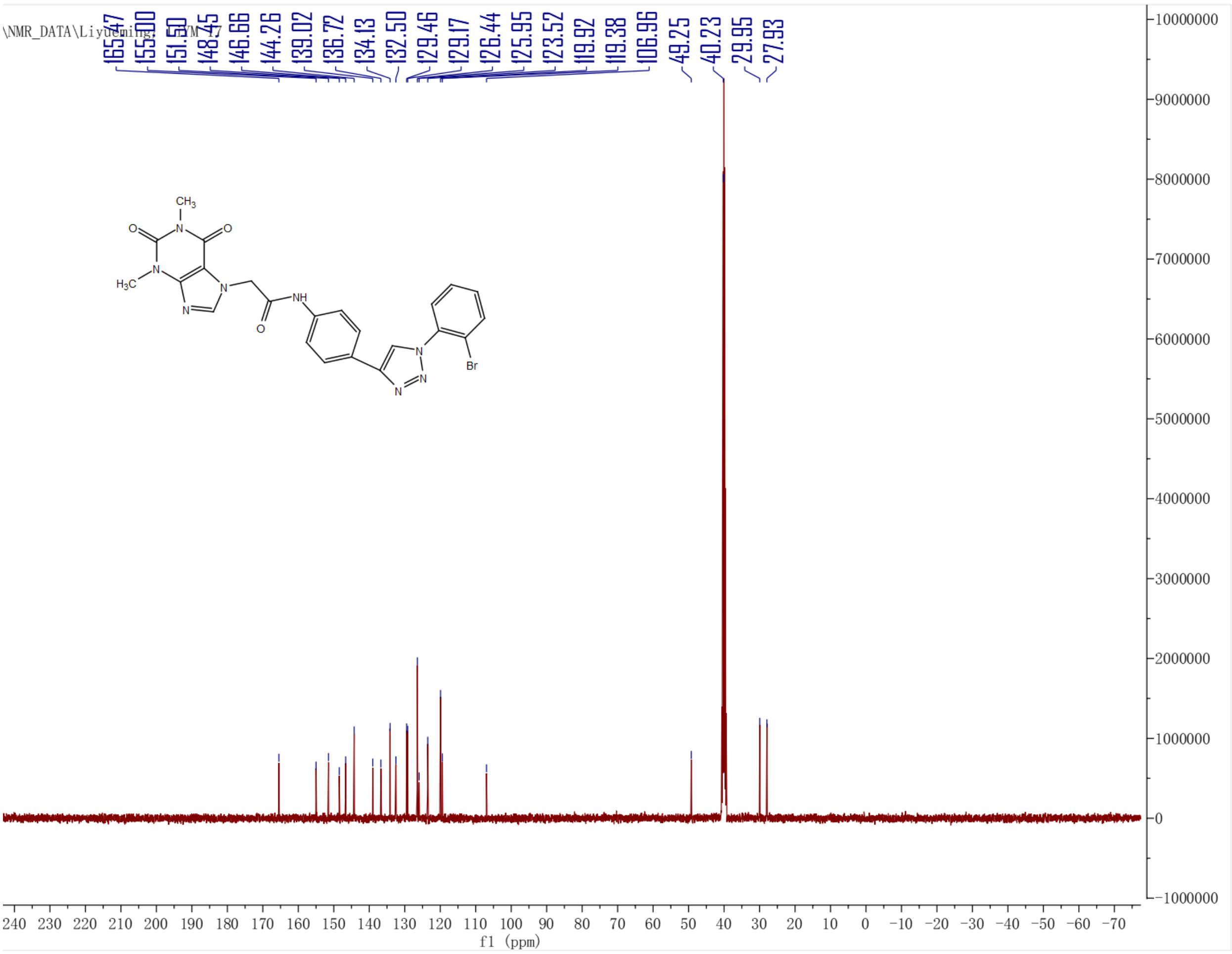

Figure S29-1. ^{1}H NMR spectrum (400MHz, DMSO-d$_6$) of compound D29

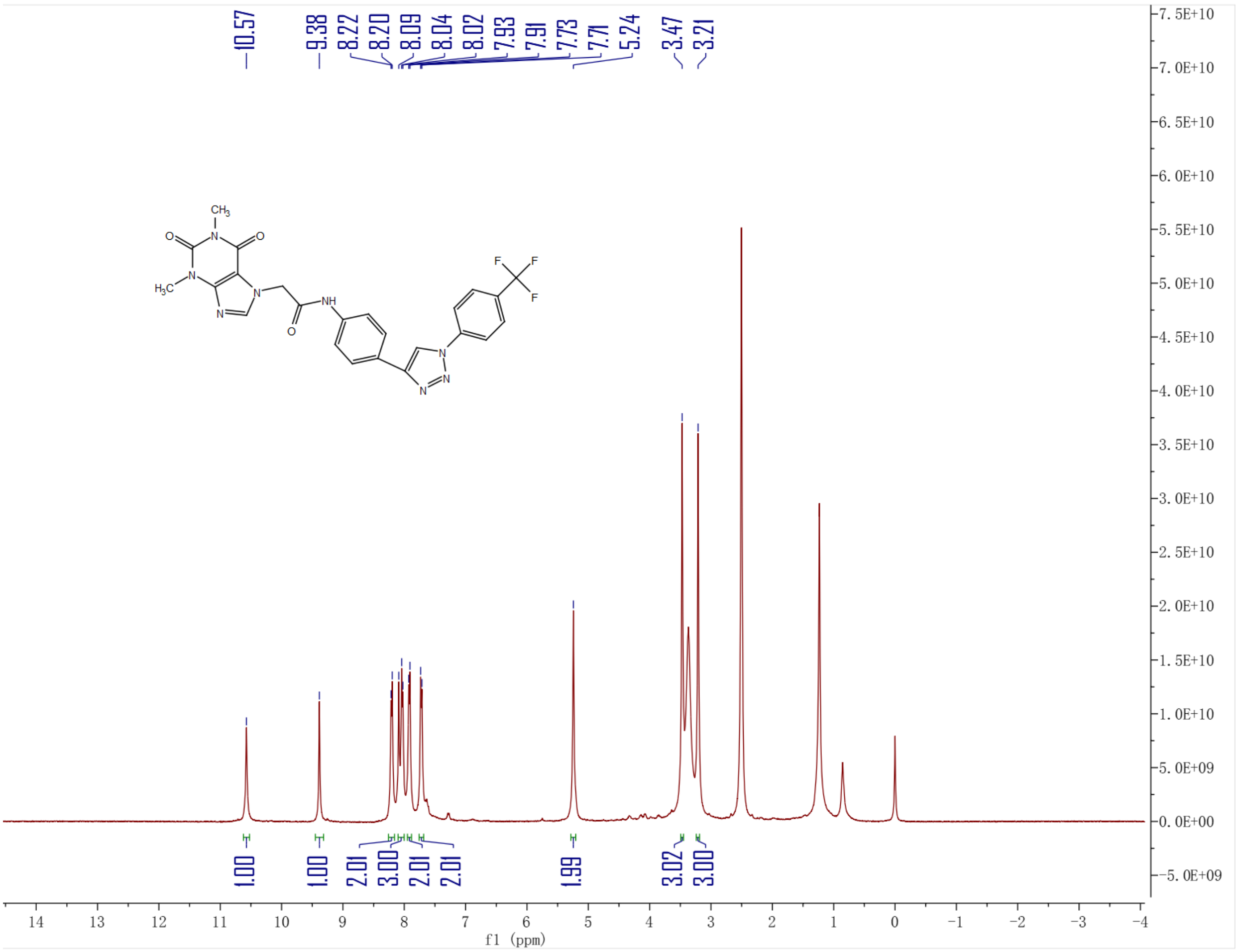

Figure S29-2. ^{13}C NMR spectrum (100MHz, DMSO-d$_6$) of compound D29

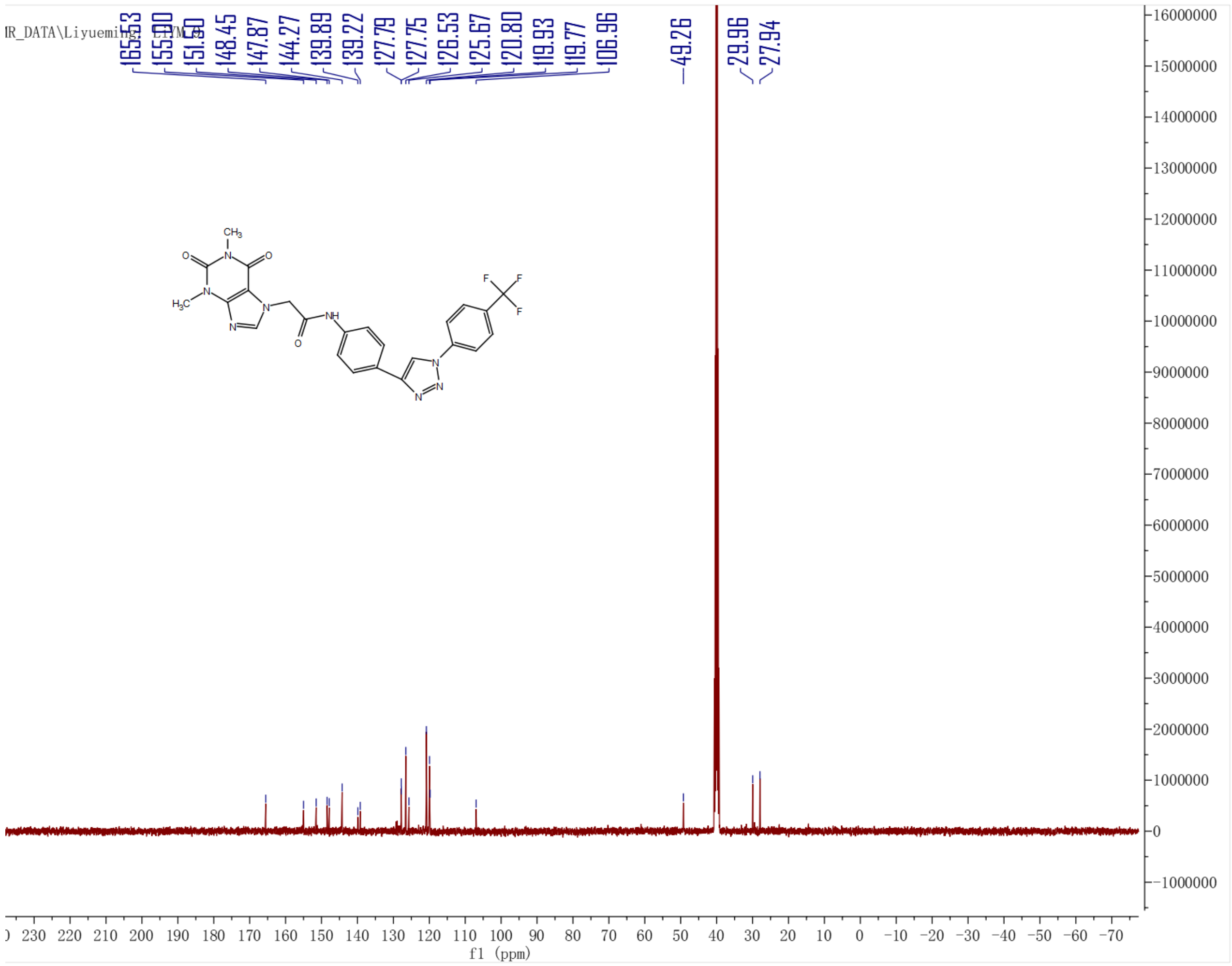

Supplement: Supplementary file 2 [file Presentation1.pdf]
